# Policy Analysis of CO_2_ Capture and Sequestration with Anaerobic Digestion for Transportation Fuel Production

**DOI:** 10.1021/acs.est.3c02727

**Published:** 2023-07-26

**Authors:** Branden E. Leonhardt, Ryan J. Tyson, Eric Taw, Marjorie S. Went, Daniel L. Sanchez

**Affiliations:** † Department of Chemical and Biomolecular Engineering, University of California, Berkeley 94720, United States; ‡ Thermo Fisher Scientific, Middleton, Wisconsin 53562, United States; § Department of Chemical and Biomolecular Engineering, University of California, Berkeley 94720, United States; ∥ Department of Environmental Science, Policy, and Management, University of California, Berkeley 94720, United States; ⊥ Department of Environmental Science, Policy, and Management, University of California, Berkeley 94720, United States

**Keywords:** renewable natural gas, electricity, transportation fuel, electric vehicles, anaerobic digestion, carbon capture and sequestration, bioenergy, climate policy

## Abstract

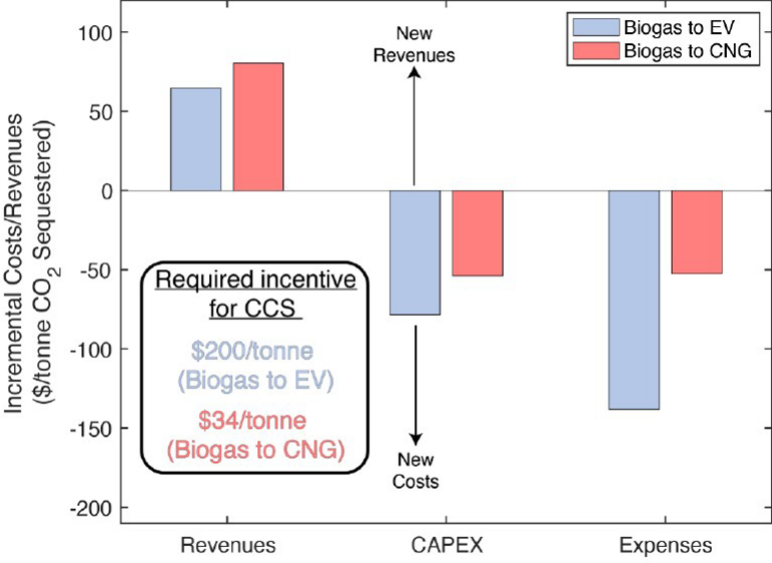

Low carbon fuel and waste management policies at the federal and state levels have catalyzed the construction of California’s wet anaerobic digestion (AD) facilities. Wet ADs can digest food waste and dairy manure to produce compressed natural gas (CNG) for natural gas vehicles or electricity for electric vehicles (EVs). Carbon capture and sequestration (CCS) of CO_2_ generated from AD reduces the fuel carbon intensity by carbon removal in addition to avoided methane emissions. Using a combined lifecycle and techno-economic analysis, we determine the most cost-effective design under current and forthcoming federal and state low carbon fuel policies. Under many scenarios, designs that convert biogas to electricity for EVs (Biogas to EV) are favored; however, CCS is only cost-effective in these systems with policy incentives that exceed $200/tonne of CO_2_ captured. Adding CCS to CNG-producing systems (**Biogas to CNG**) only requires a single unit operation to prepare the CO_2_ for sequestration, with a sequestration cost of $34/tonne. When maximizing negative emissions is the goal, incentives are needed to either (1) fund CCS with Biogas to EV designs or (2) favor CNG over electricity production from wet AD facilities.

## Abstract 2

This study considers transportation fuel policies in CA to evaluate whether producing electricity or natural gas for vehicles is more profitable at wet AD facilities and recommends facility designs to implement carbon capture and sequestration most effectively.

## Introduction

Anaerobic digestion (AD) of food and dairy waste with carbon capture and sequestration (CCS) can generate carbon-negative heat, electricity, and fuels while simultaneously reducing greenhouse gas emissions from waste decomposition.^1^ The state of California’s ambitious climate and waste management policies support new markets for these processes.^1−4^ The state’s low-carbon fuels standard (LCFS) drives the production of low-carbon and carbon-negative fuels, including those that deploy CCS.^5,6^ Furthermore, existing federal policies such as the Renewable Fuel Standard (RFS), which operates through the generation of tradeable RIN credits, and policies in the recently passed Inflation Reduction Act of 2022^7^ augment valorization of AD-CCS processes through investment tax credits (ITCs) and an expanded 45Q tax credit for carbon sequestration.

In a recently proposed rule, the Environmental Protection Agency (EPA) has provided guidance on the generation of RIN credits for electric vehicles (eRINS for EVs).^8^ If this new generation scheme were implemented, then AD facilities that burn biogas for electricity to charge EVs may flourish. Other EV infrastructure developments are already underway.^9−11^ Producing electricity does not require biogas upgrading because biogas can be combusted directly.^12^ However, implementing CCS with electricity production would require investment in biogas upgrading technology *and* CO_2_ preparation for sequestration. Although studies concerning the production of RNG and electricity from AD in CA exist,^13−15^ none have examined the impact of eRINs. We also note that several techno-economic analyses exist outside of California. Still, conclusions are sensitive to the local context, including whether the facility is under specific incentive schemes, geographical location, facility scale, or proposed product.^16−22^

Only a few existing techno-economic analyses of AD facilities include biogas upgrading *and* sequestration of the captured CO_2_.^1,23^ Only one study considered CO_2_ sequestration with AD in CA,^1^ but electricity generation for EVs was not examined, and an energy balance was not performed. Moreover, generalized cost correlations and constant performance were assumed for CCS without considering power requirements.^1^ For other process components, power needs were assumed to be met by purchasing grid power. Realistically, facility developers would examine alternatives, such as alternate utility configurations and product forms, and make comparisons to a facility without CCS. These decisions are not always straightforward, because they all impact the carbon intensity (CI) score, LCFS, RFS, and 45Q credit revenues. However, an integrated facility-scale LCA-TEA examining these options has not been developed.

To this end, we present integrated designs of wet anaerobic digestion (AD) facilities to produce transportation CNG or electricity with and without CO_2_ preparation for sequestration (CCS). While this analysis examines a case study in CA, the engineering detail presented in the Supporting Information (SI) could allow its application in many domestic and international contexts. Our results inform the optimal product form (i.e., CNG or electricity) given the potential introduction of eRINs and determine the policy conditions necessary to implement CO_2_ sequestration for all product forms. In sensitivity analyses, we consider the effects of codigestion, in which dairy manure (DM) and food waste (FW) are processed simultaneously, alternate utility configurations, and final vehicle/fuel combination effects. We conclude with recommendations to maximize the environmental benefits of AD-CCS processes.

## Methods

Our workflow is managed from a centralized Excel spreadsheet utilizing data regression against validated simulations for carbon capture, CH_4_ compression, CO_2_ liquefaction, and other units that we developed in Aspen Plus and MATLAB. Our integrated LCA-TEA model accepts specific user inputs, such as waste flow rate, weather patterns, and waste composition, and outputs detailed technical and economic information. The Excel model compiles all relevant mass and energy flow rates to perform a cash-flow analysis and compute the design’s Net Present Value (NPV). The full details of our model can be found in the SI.

Our model facility is near Fresno, CA, a major urban region approximately 30 miles from a proposed Class VI CO_2_ sequestration well in Mendota, CA.^24^ About seven renewable natural gas fueling stations are within a 30-mile radius of Fresno.^25^ We consider our facility a merchant CNG (****Biogas to CNG****) producer with its own fleet of CNG trucks.^26^ We pay an electric grid interconnection fee for **Biogas to EV** designs, as reported in previous work.^13^ We transport the liquefied CO_2_ for sequestration using another fleet of purchased trucks.^27^ Our base case facility is sized to accept 40 000 tons/year on a dry basis (SI Section 1, equivalent to 36.3 ktonnes/yr), somewhat smaller than the feed rate at existing clustered dairy AD facilities in the CA Central Valley.^28−30^ We assume tipping fees for FW are equal to local landfilling fees, while our facility does not receive tipping revenue from DM.

**Figure 1:**
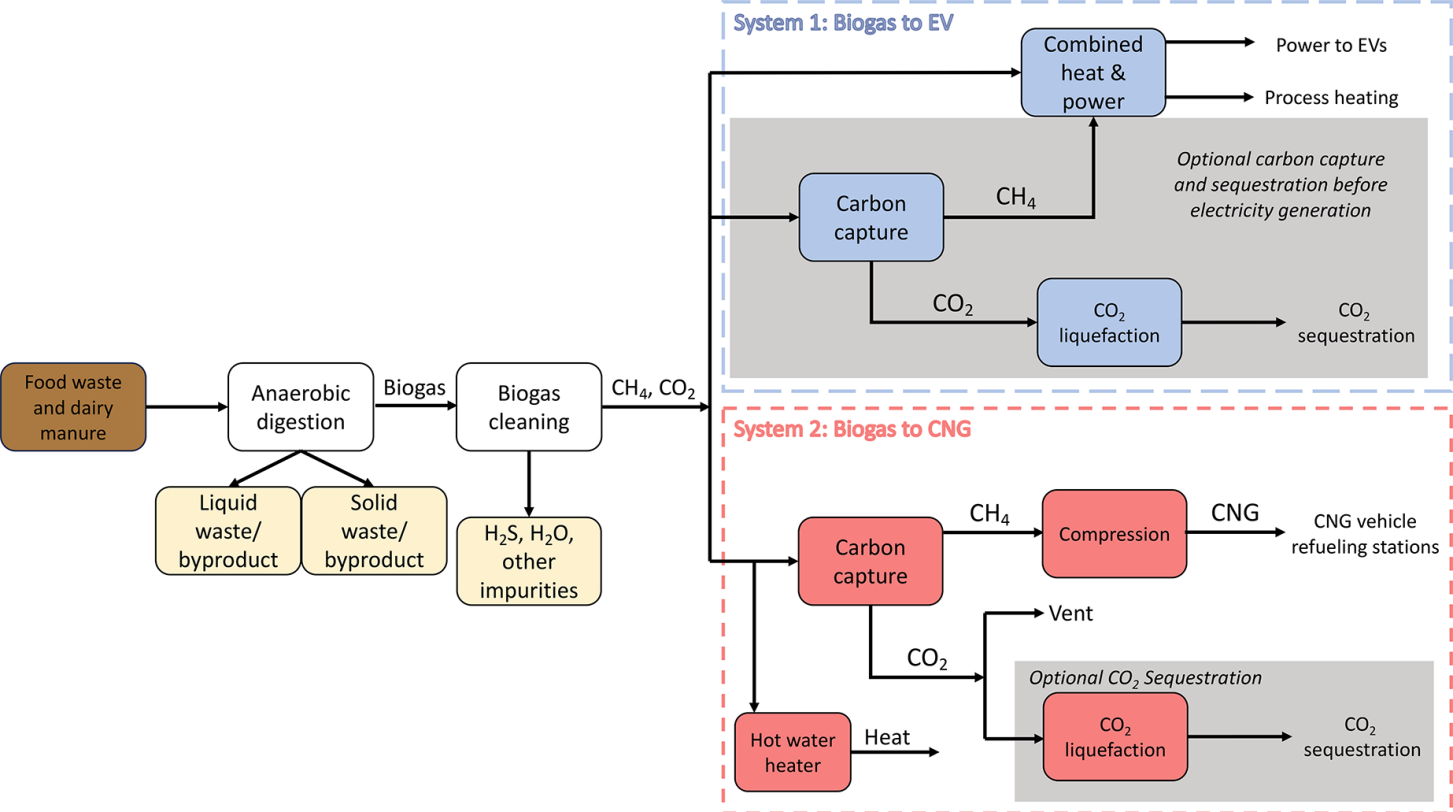
Block flow diagram of the designs compared in this work. In system 1, biogas is combusted directly in a CHP unit to produce power and heat. If CCS is implemented, then the biogas is upgraded to biomethane and then burned in a CHP unit for power and heat while the CO_2_ is trucked and sequestered. In system 2, biogas is upgraded to biomethane, which is compressed to CNG, and the CO_2_-rich stream is either vented or sequestered.

In all cases, FW and DM are shredded,^31^ blended, and pumped^32^ into mesophilic (37 °C) anaerobic digestion tanks where biogas is produced (Figure 1). Waste composition details used for the mass balance are in SI Section 2. A full process flow diagram (PFD) and process description for the AD section are in SI Section 3. In brief, we determine the mass flows (waste inlet, solid and liquid waste effluent, recycle rates, water purge rates, biogas production rates, and vapor–liquid equilibrium compositions exiting the ADs) and the power (all pumps, stirrers, and gas blowers) and heating requirements (from a 4-zone heat balance on the tanks) as a function of waste composition and scale. In this study, we conservatively assume that both liquid and solid wastes (digestates) are landfilled. However, digestates could be used as soil amendments in agriculture which can have economic and environmental benefits.^33^ More details for AD gas production, vapor–liquid equilibrium, the heat balance, and pump power are in SI Sections 4–7.

The raw biogas contains CH_4_, CO_2_, and trace undesired components, including volatile organic compounds, hydrogen sulfide, siloxanes, ammonia, and water vapor.^34^ Gas blowers move the biogas to a cleaning stage (SI Sections 8–11) consisting of a chiller, activated carbon bed, and silica gel temperature swing adsorber (TSA) to produce a CH_4_/CO_2_ mixture.

In a **Biogas to EV** design, all the biogas is sent to a spark-ignited reciprocating engine combined heat and power (CHP) system^12^ to produce electricity for export to the CA grid specifically for charging EVs (System 1 of Figure 1). Hot water is generated from CHP heat recovery to satisfy the heating requirements of the AD and silica gel bed regeneration cycle. In a design with CCS, the biogas is “upgraded” to 95% pure CH_4_ in a 3-stage membrane separation train, described in SI Section 12. The CH_4_ is burned in the engine for electricity and heat while the CO_2_-rich stream is liquefied to −20 °C at 17 bar for storage, transport in tube trucks, and injection in a nearby Class VI well.

Alternate Biogas to EV configurations are possible, such as postcombustion CCS and oxyfuel combustion, in which pure O_2_ is utilized (by separating N_2_ from air) to avoid the requirement of N_2_ separation after combustion.^23^ In principle, CCS could also be employed on the biogas and postcombustion (two separate CCS units). Postcombustion CCS would benefit from economies of scale for the larger CCS and CO_2_ liquefaction units, and it would receive greater LCFS 45Q credits. Still, it would suffer high specific energy requirements as the concentration of CO_2_ in postcombustion flue gas is smaller (∼5–15%) than in biogas (∼40%). Ultimately, for a more direct comparison with ****Biogas to CNG**** (described below), we have selected CCS to be performed before combustion (as in Figure 1). Future studies focused on Biogas to EV designs could examine all potential configurations (including detailed CI-score calculations).

Higher power/heat supply ratios, greater total efficiencies, low capital cost, and scale agreement favor using reciprocating engines as the CHP unit over other technologies;^12^ however, other CHP technologies (such as gas turbines or fuels cells) may result in higher electrical efficiencies and/or lower CI scores. We predict the electrical efficiency, heat recovered, capital costs, and operating expenses as independent functions of the capacity (kW of electricity generated) using data provided by the EPA.^12^ We linearly interpolate each dependent variable between the closest two points in the data set. Combusting biogas instead of natural gas affords changes in the efficiency of the engine.^35^ Therefore, for all biogas-fired engines in this work, we derate the electrical efficiency by 1%, as seen experimentally.^35^

In a ****Biogas to CNG**** facility (System 2 of Figure 1), biogas is upgraded to 95% CH_4_.^36^ The purified CH_4_ (RNG) is compressed to 283 bar to become CNG, stored in tube truck trailers, and distributed to fueling stations^37,26^ (SI Section 13). The CO_2_-rich stream (purity is >96%^38^) is sequestered or vented. In this design, a portion of biogas is diverted to a fired heater to generate hot water (thermal efficiency = 80%) to meet facility heat demands.^39^ All power requirements for unit operations occurring downstream of biogas diversion were decreased proportionally. Full PFDs and additional details for modeling cooling water, CO_2_ liquefaction, and CH_4_ compression, along with validations of our calculations using reputable vendors and literature, are in SI Sections 14, 15, and 16, respectively.

All wet AD designs in this work produce >1000 N m^3^/h of biogas. At these scales, it has been shown that biogas upgrading technologies exhibit similar economics.^40^ Here, we require high methane recoveries to meet the purity requirements of natural gas vehicles (95% CH_4_) and CO_2_ sequestration (96% CO_2_). Therefore, we utilize a high-recovery three-stage membrane separation train as the CC technology (see SI Section 12 for full development). Before implementation, we validated our MATLAB simulations to ensure accurate predictions of separation performance, power requirements, and capital costs (SI Section 12). We then utilized regressions on MATLAB simulation results in our Excel TEA model to predict the unit’s performance at other process conditions within the range simulated.

CI scores, necessary to calculate carbon credits for the LCFS,^41^ vary across the fuel types and wastes considered in this work.^42^ More details concerning initial CI scores and CI score adjustments are discussed in SI Section 17. LCFS credit generation is calculated from Section 95486.1 of the LCFS regulation using gasoline as the reference fuel.^41^ The market value is assumed to be $110/tonne in the base case. We consider a range of LCFS values when exploring the economics of CCS. For Biogas to EV designs that produce power for battery electric vehicles (BEVs), or plug-in hybrid electric vehicles (PHEVs), we use 3.4 as the appropriate energy economy ratio (EER).^41^ For the RFS, we compute the fraction of biogas generated by FW and DM and apply D5 and D3 RIN credit generation independently.^8^ A “RIN” is 22.6 kWh for CNG. An “eRIN” corresponds to 6.5 kWh of electrical energy.^8^ D3 and D5 RIN credits are assumed to be $3.11/RIN and $1.55/RIN, respectively, based on 2022 trading values.^43^ eRIN credits are only generated by 80.5% of the power produced at the biogas facility to account for line-loss (5.3% losses) and EV charging station efficiency (85%).^8^ The 45Q tax credit is direct-pay (i.e., revenue) at $85/tonne with a minimum sequestration threshold of 12,500 tonnes per year.^7^ The Inflation Reduction Act (IRA) includes new investment tax credits (ITCs) to biogas facilities with “qualified biogas property” of up to 50% of capital expenses.^7^ This work assumes that our facility meets the requirements to receive a 30% ITC. We assume qualified biogas property includes all property except CO_2_ liquefaction and trucking units. These benefits are implemented by directly reducing the initial capital investment by 30% for qualified property. Table S18.1 compiles many key economic, policy, and process variables and defines the “base case” used throughout the paper.

## Results and Discussion

### Mass Balances and Transportation Fuel Production

The AD facilities in our case study process between 148 and 182 ktonnes/yr of wet waste and produce 1100 to 3000 N m^3^/h of biogas, depending on the waste processed (Table 1). The biogas composition depends on the waste, with ∼6% higher CH_4_ contents available from FW biogas vs DM biogas. Biogas to EV facilities produce 2–7 MW of power depending on the waste, equivalent to 3%–10% of EVs’ total 2021 electricity consumption in CA.^44^****Biogas to CNG**** facilities produce 5–18 MW of CNG, satisfying 0.3%–1% of the 2021 CA natural gas vehicle consumption.^44^ With CO_2_ sequestration (CCS), they sequester 6–16 ktonnes of CO_2_ per year, equivalent to the annual emissions of 1500–3500 typical gasoline-powered passenger vehicles. As a result, CI scores are lowered by an additional 15–30 gCO_2e_/MJ. Accounting for biogas diversion, CO_2_ sequestration, trucking operations, engine efficiency, and EER adjustment (see Methods), CI scores range from −50 to −110 gCO_2e_/MJ and −190 to −365 for FW and DM, respectively. These values are within ranges reported elsewhere.^42^ With combined CO_2_ sequestration and negative emissions (from avoided landfill and agricultural emissions associated with FW and DM), the facilities each remove 41–65 ktonnes of CO_2e_ annually. A 100%FW facility digests 20% less waste on a wet-weight basis but produces three times as much biogas owing to its higher biomethane potential. This indicates that DM facilities in operation today can greatly enhance biogas production rates through codigestion with FW, but downstream equipment sizes must also be increased. CO_2_ sequestration rates are slightly lower for ****Biogas to CNG**** because a portion of biogas must be diverted to the fired heater to generate hot water for AD. In contrast, hot water is a byproduct of electricity generation at Biogas to EV facilities.

**Table 1:** CNG or Electricity Produced, CO_2_ Sequestered, CI Scores, and Negative Emissions for All AD Designs in This Work^a^,^b^,^c^

	**Biogas to EV**	**Biogas to EV (CCS)**	****Biogas to CNG****	****Biogas to CNG** (CCS)**
electricity production rate (MW)	2.04/4.89/7.19	1.59/4.08/6.08	n/a	n/a
CNG production rate^d^ (MW)	n/a	n/a	5.62/12.5/18.1	5.62/12.5/18.1
CO_2_ sequestration rate (ktonnes/yr)	n/a	6.36/11.1/14.9	n/a	5.84/10.6/14.3
CI score FW/DM (gCO_2e_/MJ)	–50.1/–190	–65.2/–210	–85.6/–334	–110/–365
negative emissions rate^e^ (ktonnes CO_2e_/yr)	46.7/44.4/40.9	51.8/53.5/53.2	61.0/54.3/48.8	66.6/64.5/62.6

a (CCS) indicates CO_2_ capture and sequestration is performed,

b All table entries are given for 0%FW/50%FW/100%FW AD designs except for the CI score.

c Waste processed, biogas flow rate exiting the AD, percentage of biogas from FW, and methane mole% leaving the AD is 182/164/148 wet ktonnes/yr, 1080/2150/3015 N m^3^/h, 0%/78%/100%, and 55.7%/60%/61.3%, respectively.

d 95% CH_4_ purity.

e This flow multiplies the CI score (with the added contribution from CO_2_ sequestration) of the transportation fuel by the annual energy produced, all EER adjusted. n/a = not applicable

### Facility Energy Supply and Demand

**Figure 2:**
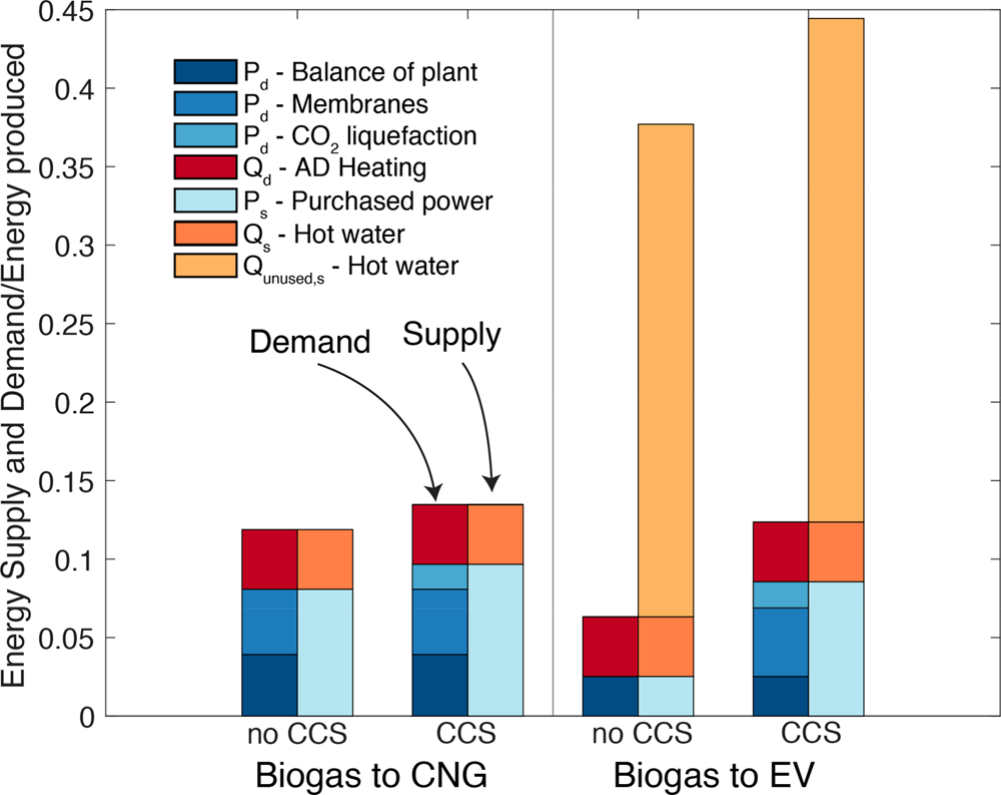
Power and Heat supply/demand for wet AD facilities (50% FW). Each pair of bars displays the energy demand (left) and supply (right) relative to the energy of the prediverted biogas as a common reference. A subscript “d” in the legend indicates an energy demand, while a subscript “s” indicates a supply.

An integrated energy balance ensures that heat and power demands for the facility are met efficiently for each scenario (Figure 2). For simplicity, we focus on the results for the 50%FW scenario, but we discuss values for 100%DM and 100%FW when relevant. The 3-stage membrane carbon capture system (when utilized) consumes 50% of power demands, with the balance from CH_4_ compression to CNG (15.5%), AD stirring (14.2%), blowers (5.9%), pumps (1.7%), chillers (3.6%), FW shredders (5.3%), and blenders (0.4%). Figure 2 shows that the CO_2_ liquefaction unit adds about 100 kWh/tonne of CO_2_ liquefied to the power demand (18% of the total power).

Because we maximize the engine capacity to produce electricity in Biogas to EV facilities, nearly 90% of the hot water generated from heat recovery goes unused (Figure 2). A **Biogas to EV (CCS)** (vs **Biogas to EV**) facility includes carbon capture and CO_2_ liquefaction. These unit operations increase power demands relative to the base case by over 300%, but power demands remain lower than for the corresponding ****Biogas to CNG**** designs. The fraction of unused hot water ranges from 83 to 91% for 100%DM and 100%FW **Biogas to EV** scenarios, respectively, regardless of CCS implementation. The overabundance of hot water would make **Biogas to EV** facility heat supplies robust during winter when heating requirements for the AD increase. In contrast, ****Biogas to CNG**** facilities with fired heaters must adjust diversion rates seasonally. Using the weather ranges observed near Fresno, we calculate that biogas diversion rates range from 2% in the summer to 7.5% in the winter. All power generated is exported to EVs, and all power demands are purchased because this configuration is the most economical (see section NPV).

### Capital Costs (CAPEX), Operating Expenses (OPEX), and Revenues

To establish the overall and relative economic viability of the scenarios we considered, we calculate each design’s CAPEX, OPEX, and revenues. We report all monetary values in 2021 USD.^37^ Capital expenses across all designs range from 70 to 100MM$ and depend on the product (electricity or CNG) and waste composition digested (Table S18.3), in agreement with other academic sources.^13^ The CAPEX of the AD section is 70–80% of the total CAPEX (Figure S18). 100%DM designs are $13MM and $18MM less expensive than 100%FW designs for **Biogas to EV** and ****Biogas to CNG****, respectively, because they produce less biogas (Table 1) and require less capital equipment to handle the gas (Table S18.3). **Biogas to EV** designs are more capital-intensive than ****Biogas to CNG**** of the same type because the cost of a high-capacity reciprocating engine eclipses the sum of carbon capture, CH_4_ compression, and trucking equipment at these scales. When we incorporate CCS into the design of **Biogas to EV** facilities, a 3-stage membrane carbon capture unit is now required along with CO_2_ liquefaction and a tube truck (Figure 1). Thus, when CCS is included, the incremental CAPEX is more significant for **Biogas to EV** than ****Biogas to CNG**** (Table S18.3).

The sale of transportation fuel credits dominates revenues (85% of the total) for all designs (Figure S19.1). This indicates that AD facilities should maximize biogas production and credit generation, which occurs when digesting high fractions of FW (Table 1). In other AD studies that did not target the low carbon transportation fuel market, tipping fee revenues dominate.^13,14^ While LCFS credit revenues are comparable for both **Biogas to EV** and ****Biogas to CNG**** designs ($10.5MM/yr and $10.2MM/yr, respectively), eRIN credit revenues for **Biogas to EV** are nearly 20% greater than RIN revenues for ****Biogas to CNG**** ($10.6MM/yr and $9MM/yr, Figure S19.1). eRIN credit revenues for **Biogas to EV** are still 10.3% greater than for ****Biogas to CNG**** when **Biogas to EV** power demands are satisfied parasitically (Figure S20.1). The details behind the calculated revenues are nuanced because the LCFS and RFS policies adjust credit generation for distinct fuel types differently,^8,41^.^45^ In brief, eRIN revenues will be larger than RIN revenues for the same quantity of biogas if the electrical efficiency of the engine is greater than 28.8%. The apparent similarity of LCFS revenues is mainly fortuitous. We present more details in SI Section 21 for the interested reader.

Operating expenses range from $10–$13MM per year (Figure S19.1), corresponding to 12.5%–14% of capital expenses; this result is slightly conservative relative to industrial sources.^46^ Going from **Biogas to EV** to ****Biogas to CNG**** without CCS, the most significant changes in OPEX are increased purchased power expenses and eliminated CHP maintenance.

### NPV

**Figure 3:**
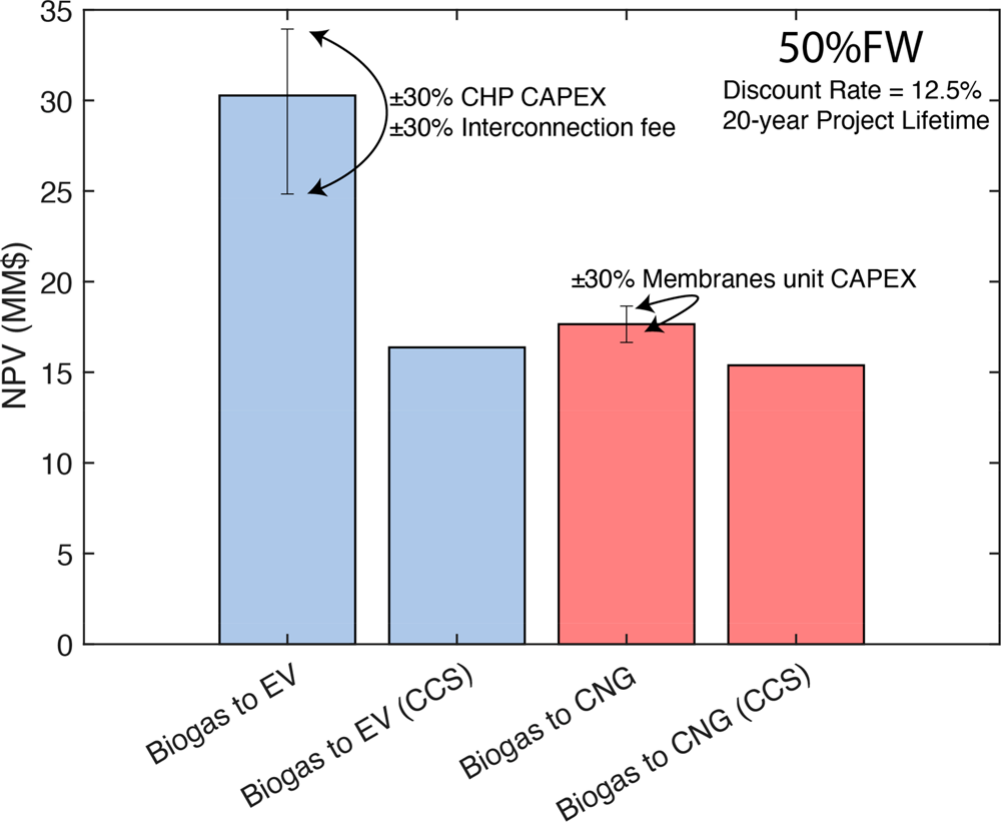
Net present values (MM$) of wet AD designs for multiple energy products and with or without CCS (50%FW) under base policy landscape. Table S22.1 displays the relevant cash-flow assumptions used to calculate NPV. The error bars display the range of NPVs calculated when the adjacent variables were modified by ±30%. We did not alter the CNG compressor or CNG tube truck CAPEX costs because vendors verified these.^26^ The NPV change from the grid interconnection fee range was negligible (±$0.04MM).

Using NPV as a metric, **Biogas to EV** facilities are economically favored relative to ****Biogas to CNG**** facilities (Figure 3). While CAPEX is higher for **Biogas to EV** (Table S18.3), and OPEX is comparable (Figure S19.1), enhanced eRIN revenues are more significant. This trend holds whether the facility digests 100%FW or 100%DM (SI Section 19). Due to low biogas production rates and significant capital costs for stainless steel tanks, 100%DM facilities exhibit negative NPVs under the base policy landscape. Without eRINs, **Biogas to EV** NPVs are all negative, with all else held equal, demonstrating the significance of this new revenue stream. We estimated the effects of uncertainty in various parameters such as CHP CAPEX, interconnection fee, and membranes unit CAPEX, but the trends endured.

A utility configuration where power is supplied parasitically decreases the NPV for **Biogas to EV** by $3.7MM (Figure S20.2). Since transportation fuel credits dominate revenues, we do not recommend diverting biogas for on-site energy generation (see discussion in SI Section 20). We also investigated the effect of the LCFS Energy Economy Ratio (EER), which changes with vehicle/fuel combination, on the NPV for both facilities. When EERs are 2.8 or greater, encompassing nearly all vehicle/fuel combinations, **Biogas to EV** designs remain more profitable than ****Biogas to CNG**** designs within our estimated uncertainties (Figure S23). Without the IRA ITCs, NPVs change by −$25MM and −$23MM for **Biogas to EV** and ****Biogas to CNG**** designs, respectively. We note that this NPV change is larger than EER or CAPEX uncertainties, highlighting the significance of the IRA on wet AD economics. Adding CCS to a **Biogas to EV** design changes the NPV by −$14MM instead of only −$2.3MM for ****Biogas to CNG****.

Volatility in AD tank costs, LCFS credits, RFS credits, waste tipping fees, electricity prices, and biogas production rates (from heterogeneous waste compositions) would impact all facilities almost equivalently regardless of fuel type. Therefore, the trends predicted here are significant. We note that CHP electrical efficiency (**Biogas to EV**) and carbon capture unit CH_4_ recovery (****Biogas to CNG****) would substantially affect these trends, given the strong dependence on transportation fuel credits.

### Effects of CCS on NPV

**Figure 4:**
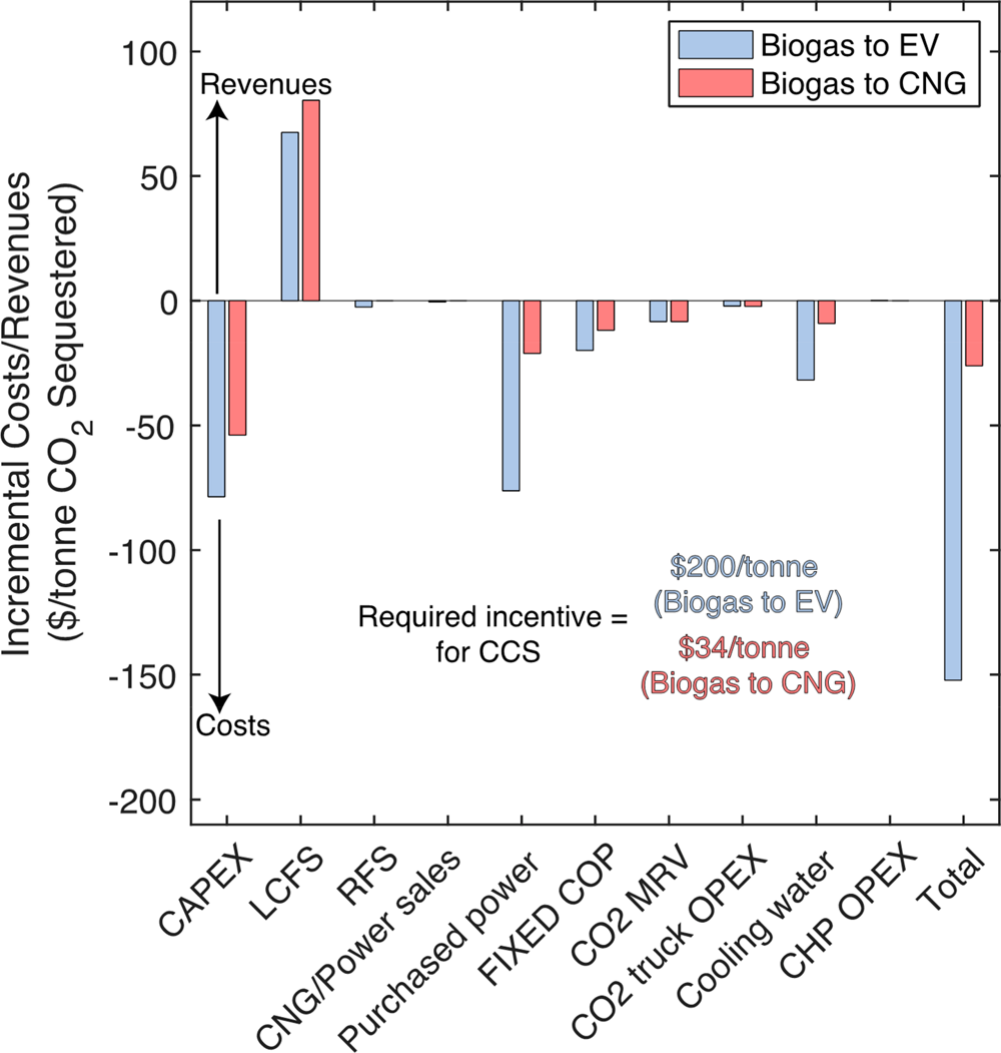
Incremental costs and revenues incurred by adding CCS to wet AD designs. Positive values indicate new savings or revenues, while negative values are new costs. Values are expressed as annualized costs that provide the same net-present values (costs/revenues) as actual cash flows across the project lifetime. “FIXED COP” are fixed costs of production, including labor, supervision, overhead, maintenance, land, and insurance (Figure S19.1). The sum of costs and revenues gives the “total,″ but we note that some manipulation is needed to convert this value into a required incentive that follows the actual cash-flow schedule. This manipulated value is reported on the bottom, middle of the chart.

To understand the factors governing the NPV differences between designs with and without CCS, we calculated the incremental costs and revenues associated with including CCS for **Biogas to EV** and ****Biogas to CNG**** facilities (Figure 4). Cash-flow schedules vary for each cost and revenue component (SI Section 22). We calculated each component’s net-present cost or revenue and normalized them by the project lifetime to allow for direct comparison. The sum of these annualized cash-flows furnishes the same net-present cost/revenue as the cash-flows following the actual schedule. Surprisingly, adding CCS to wet AD designs results in many incremental parameter changes. This fact demonstrates the importance of considering a fully integrated LCA-TEA because this level of detail is missed from studies examining CCS in isolation^40^ or using oversimplified regressions predicting cost and performance.^1,13^

For the **Biogas to EV (CCS)** design, CCS includes a 3-stage membrane separation unit, a CO_2_ liquefaction unit, and tube trucks for transporting liquefied CO_2_. For the **Biogas to CNG (CCS)** design, a 3-stage membrane separation train is already included, so CCS includes only liquefaction and trucks.

The highest additional costs for the **Biogas to EV** design with CCS are the purchased power required for the 3-stage membrane separation and CO_2_ liquefaction units (−$76/tonne, 35% of costs) and the annualized CAPEX of those units plus tube trucks (−$79/tonne, 36% of costs). The CAPEX can be further broken down into contributions from the membrane system (44%), CO_2_ liquefaction unit (34%), and CO_2_ trucks (22%). CO_2_ sequestration directly lowers the CI score of the product electricity (Table 1) and enhances LCFS revenues ($68/tonne incrementally). RFS credit generation is largely unchanged by adding CCS since it is not CI score-dependent. The sum of incremental costs and revenues is −$152/tonne. Converting this value into an incentive based on our assumed cash-flow schedule yields $200/tonne. This incentive is much larger than federal incentives (45Q at $85/tonne). Therefore, a CO_2_ sequestration package is not economically attractive for a **Biogas to EV** facility under the base policy landscape.

Alternatively, the ****Biogas to CNG**** design requires only $34/tonne, well within the federal 45Q incentive. The largest incremental expense is CO_2_ liquefaction and trucking CAPEX (−$54/tonne), followed by purchased grid power (−$21/tonne). This analysis shows that a CO_2_ sequestration package would be profitable for a **Biogas to CNG** facility that meets the 45Q sequestration threshold (12,500 tonnes/year).^47^

Figure 4 was generated at a fixed scale (50%FW). Modifying the facility scale only influences the incremental CAPEX and fixed costs of production (FIXED COP) components (Figure S24 for **Biogas to CNG**). Interestingly, all other components remain constant as scales change.

### Effects of Policy Landscape on CCS Feasibility

**Figure 5:**
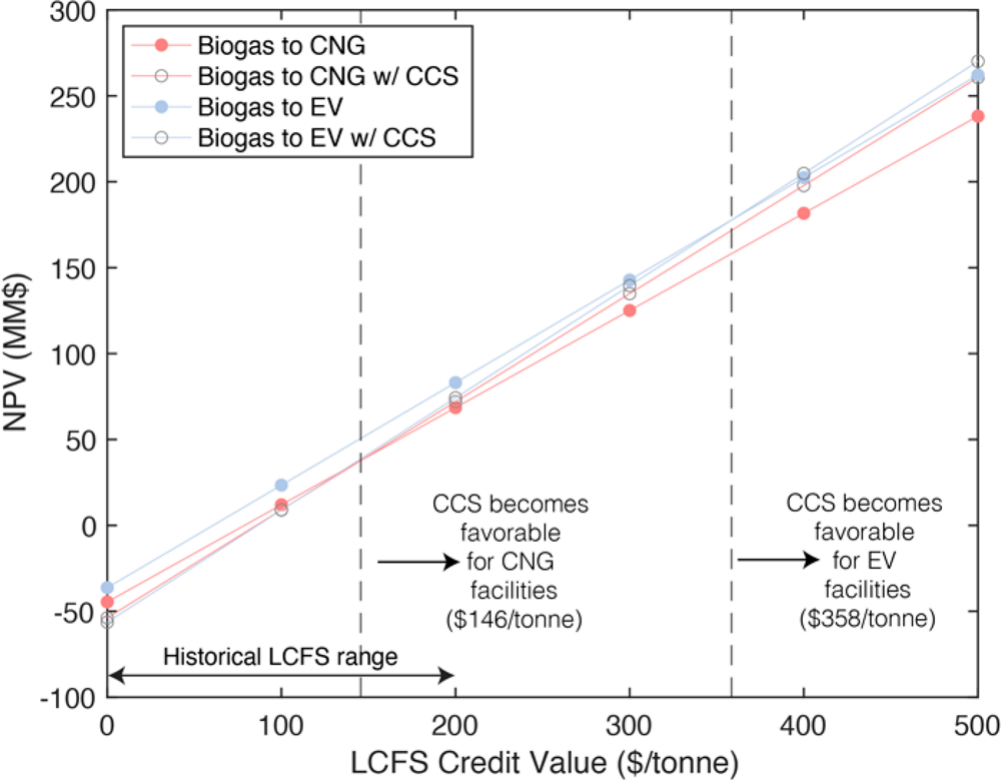
Sensitivity analysis of LCFS credit value on project NPV for 50%FW feed. Crossover points where the NPV of a facility with CCS eclipses the facility without CCS are marked with dashed lines. The designs in the figure receive RFS credits (RINs and eRINS) at base values (Table S18.1), but they are not receiving the 45Q, which has the effect of shifting both dashed lines to the left by approximately $85/tonne. The dashed line position is a weak function of the initial CI score of the fuel, RFS credit values (both D3 and D5), waste tipping and disposal fees, waste composition (for a fixed CO_2_ flow rate), CO_2_ mole fraction in the biogas, CNG and electricity sale prices, CHP engine electrical efficiency, and vehicle/fuel combination (EER value). For additional sensitivity analyses, see SI Section 25.

As expected, the NPVs for all facilities increase monotonically as LCFS credit values increase (Figure 5). Without the LCFS, all NPVs are negative, indicating the LCFS is needed for profitability, with all else held equal. Because LCFS credit generation is CI score-dependent, transportation fuels with low CI scores become more attractive as credit values increase. As a result, facilities that perform CCS become more attractive than designs without CCS when the LCFS trades above a critical value. For a **Biogas to CNG** design, this value occurs at $145/tonne, well within the range seen historically.^48^ For **Biogas to EV** facilities, more considerable incremental capital and purchased power expenditures (Figure 4) require the LCFS to be $358/tonne; historically, the LCFS has never traded above $210/tonne.

Additional credits are required for CO_2_ sequestration to be profitable below those critical LCFS values. If the **Biogas to CNG** design were to earn the federal 45Q CO_2_ sequestration credit (at $85/tonne), then the LCFS would only need to trade above $57/tonne to motivate CCS. The LCFS has traded above $48/tonne within the last five years.^48^ For the **Biogas to EV** design, the LCFS must be at least $253/tonne when receiving the 45Q at $85/tonne, which is out of its historical range.^48^ We performed a sensitivity analysis (SI Section 25) on the minimum required CO_2_ sequestration credit with respect to facility scale and uncertainties in our estimates of purchased power costs and CCS CAPEX since these were found to be significant (Figure 4) and came to similar conclusions.

### Environmental Implications and Recommendations

With eRINs, the EPA is partly attempting to reduce the carbon intensity of the transportation sector.^8^ Our study highlights an unintended consequence of this policy, if implemented, in the context of wet AD designs. With the inclusion of eRINs, the most profitable design produces electricity for EVs from biogas combustion (**Biogas to EV**), but CCS is not economically compatible under current policy conditions (Figure 3). This is due to significant incremental capital and purchased power expenses exclusive to the **Biogas to EV** design (Figure 4). We examined the influence of scale (Figure S25.1) and potential uncertainties (Figure S25.2) on our calculations; however, they did not change our conclusions.

Carbon dioxide removal (CDR), such as the designs with CCS presented here, is necessary to achieve emissions reduction goals set forth by lawmakers in CA.^2^ CCS, when added to **Biogas to EV** or **Biogas to CNG**, would sequester an additional 6–16 ktonnes of CO_2_ per year depending on the waste digested (Table 1). However, the **Biogas to EV** design without CCS simultaneously exhibits the highest NPV (Figure 3) and lowest negative emissions rate (Table 1). Thus, when maximizing negative emissions is the goal, additional incentives are needed to favor CNG production from biogas over electricity production. Once **Biogas to CNG** designs become adequately incentivized over **Biogas to EV** (as they are without eRIN implementation), CCS can be adopted readily with currently existing CO_2_ sequestration credits (45Q and LCFS combinations, Figure 5). Our study also illuminates the environmental benefits of lowering the 45Q sequestration thresholds (Figure S25.1). In SI Section 27, we discuss opportunities for CCS with wet AD designs in regions outside CA.

If additional incentives for **Biogas to CNG** are not possible, then CCS may be performed with **Biogas to EV** another way. Instead of investing in carbon capture and liquefaction of the CO_2_ contained in biogas, **Biogas to EV** facilities could use excess hot water (Figure 2) and grid power to provide heat and power for a Direct Air Capture (DAC) unit adjacent to the facility. Recent studies concerning DAC have surmised that heat and power requirements are about 6 GJ/tonne and 1.5 GJ/tonne, respectively.^49^ If the **Biogas to EV** study in this work were to employ DAC by using all available unused hot water (Figure 2) and CA grid power, it would be able to remove nearly 20 000 tonnes of CO_2_ per year from the atmosphere. This value is *double* the CO_2_ sequestered from biogas separation. Assessing the economic feasibility of a DAC scenario is outside the scope of this work, but we note that the 45Q credit is $180/tonne for DAC instead of the $85/tonne for source capture.^47^ On top of this, LCFS credits would be boosted from the lower CI score resulting from the doubled sequestration volume.

In the future, we suggest exploring alternative **Biogas to EV** CCS configurations, such as postcombustion, oxyfuel, or DAC. It would also be worthwhile to examine various electricity generation equipment (such as gas turbines or fuel cells), along with their associated emissions factors and their effect on negative emissions rates and NPV. Finally, we suggest considering other types of AD reactors, like covered lagoons, which could be a more cost-effective option compared to the stainless steel tanks used here.

## Supporting Information Available

Additional process descriptions and diagrams, simulations and data regression for processes, additional economic details, additional sensitivity analyses, and discussion (PDF)
