# Supplementary material for: Policy Analysis of CO2 Capture and Sequestration with Anaerobic Digestion for Transportation Fuel Production
Source: Environ Sci Technol. 2023 Jul 26;57(31):11401–9. doi: 10.1021/acs.est.3c02727 (PMC10413946; doi:10.1021/acs.est.3c02727)
Supplement: Supplementary file 1 — es3c02727_si_001.pdf [file es3c02727_si_001.pdf]

Supplementary Materials for

Policy analysis for CO_2 capture and sequestration with anaerobic digestion for transportation fuel production

Branden E. Leonhardt, Ryan J. Tyson, Eric Taw, Marjorie S. Went, Daniel L. Sanchez

Table of Contents

1. Dairy manure (DM) and food waste (FW) near Fresno 3

2. Waste compositions and densities 4

3. AD section PFD and process description 5

4. Anaerobic digestion kinetics 8

5. AD VLE and solubility calculations...11

6. Heat transfer analysis for anaerobic digesters 13

7. Pump power requirements...18

8. Chiller power and economic calculations...19

9. Gas blower power and economic calculations 21

10. Activated carbon bed calculations 23

11. Silica gel bed calculations 24

12. Three-stage countercurrent hollow fiber membranes separation: simulations and data regression 25

13. Transportation of CNG and liquefied CO_2: cost assumptions...31

14. Cooling water temperature and makeup 32

15. CO_2 liquefaction 33

16. CH_4 compression to CNG 38

17. CI score calculations and adjustments...40

18. List of key economic and process variables, purchased costs for major equipment, installation factors, OSBL, D&E, contingency, and total CAPEX for each design 41

19. Revenues and OPEX for all designs, and NPVs for facility designs with 100%FW and 0%FW waste compositions...46

20. Diversion of power to satisfy plant demands parasitically - Biogas to EV (Parasitic).........50

21. Discussion of calculated revenues from RFS and LCFS...52

22. Cash flow analysis (NPV)...53

23. Effect of Energy Economy Ratio (EER) on NPV 54

24. Breakdown of incremental costs and revenues from CCS as a function of scale 56
25. Sensitivity analysis of parameters that govern CCS feasibility with wet AD designs..........57
26. Current facilities in CA that can perform profitable CO_2 sequestration 59
27. Opportunities for CCS with wet AD designs outside of CA...60
28. Plotting functions 61
References...62

1. Dairy manure (DM) and food waste (FW) near Fresno

Recent livestock and crop reports show breeding stocks of nearly 100,000 head of cattle in the area; feeder stock, calves, and cull stock contribute an additional 120,000 head [1]. An average manure production rate of 100 lb/day per cow amounts to 1,790,000 tons of dairy manure (DM) per year. As a starting point, we assume our facility receives 5% of the manure produced in the area, nearly 200,000 wet tons per year. In Fresno County, FW consists of high-moisture solids from municipal solid waste, and its generation fluctuates seasonally, with lows in February (~88 ktonne/yr, dry basis) to highs in August during almond production season (~1750 ktonnes/yr, dry basis, mostly almond culls) [2]. Because moisture content in wastes varies, we input waste flowrates on a bone-dry basis using average moisture contents for both wastes (see Table S2.1).

2. Waste compositions and densities

We determined average total solids (TS) and volatile solids (VS) contents for food waste (FW) and dairy manure (DM) from multiple literature sources (Table S2.1). We assume the balance for each waste is water.

Table S2.1: Total and volatile solid compositions across the literature

Reference	Waste type	%TS	%VS
**[3]**			
UK – Eastleigh	FW	25.89	24.00
Finland – Forssa	FW	27.02	24.91
Italy – Treviso	FW	27.47	23.60
USA – San Francisco	FW	30.90	26.35
China – Beijing	FW	23.10	21.00
South Korea – Yongin	FW	18.10	17.10
**[4]**	FW	13.87	12.03
**[5]**	FW	29.40	28.02
**[6]**	FW	30.90	26.35
**[7]**	FW	24.30	22.50
**[4]**	DM	38.50	28.80
**[8]**	DM	12.25	10.97
**[9]**	DM	10.20	8.52
**[10]**	DM	12.40	10.21
**[11]**[1]			
Lactating cow	DM	28.04	9.93
Dry cow	DM	16.46	12.47
Young cow	DM	21.35	2.24
Average	FW	24.46	22.09
Standard Dev.	FW	5.66	4.88
Average	DM	19.89	13.33
Standard Dev.	DM	10.3	6.57

[1]values below for unbolded entries are within the bolded reference

Average total solids contents for FW streams tend to be higher on average than for DM, while the variability reflected in the standard deviations is more significant for DM.

3. AD section PFD and process description

In the wet anaerobic digestion section (Figure S3), food and dairy waste are received and stored in stainless-steel buffer tanks. FW is pretreated in an industrial shredder unit (Franklin Miller [12]), then mixed with water and DM using a stainless-steel helical ribbon agitator in a stainless-steel homogenization tank. The mixture is pumped with two submersible grinder pumps (Pentair Myers, VS50 [13], maximum trim size) through a heat exchanger that uses hot water from an on-site CHP unit. The pre-heat temperature required to maintain the mesophilic digestion temperature, 37 °C at 1.05 bar, is determined from a detailed energy balance on the AD tanks that utilizes local weather data and calculates heat transfer through the sides, tops, ground, to the biogas phase above the liquid, radiation from the sun and emission from the tank, and heat of reaction and shaft work terms (see SI Section 6).

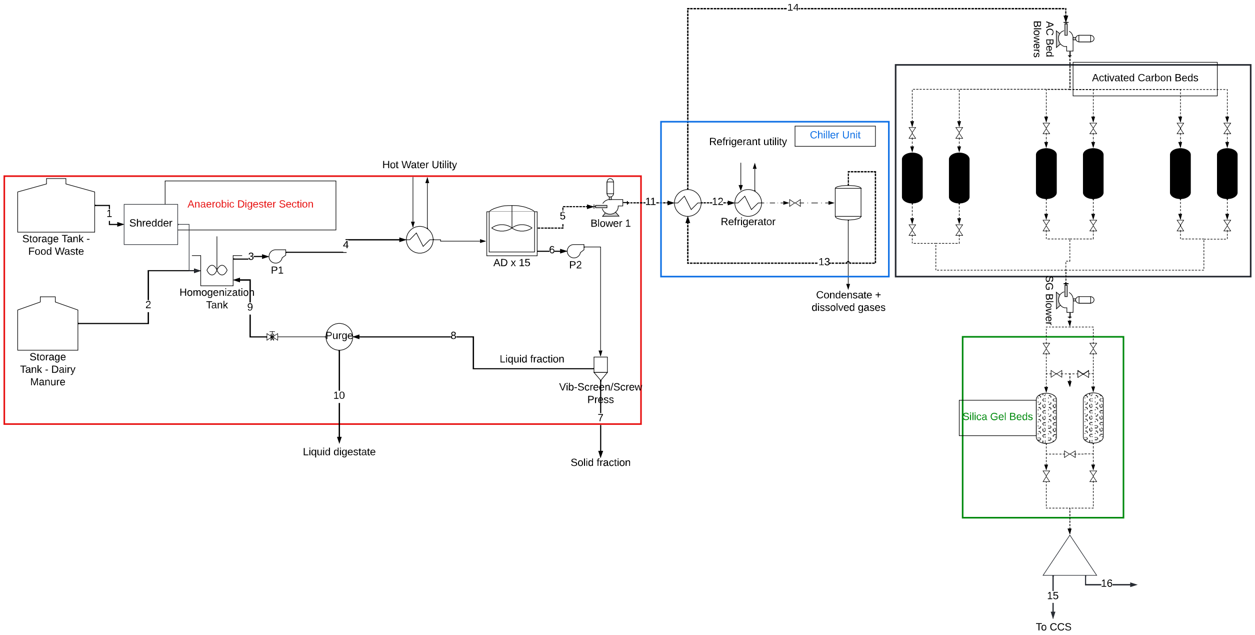

Figure S3: Process flow diagram of the AD and biogas cleaning sections for all designs in this work. For the Biogas to CNG design, a portion of biogas (stream 16) is diverted to an on-site fired heater to generate hot water for the "hot water utility". Otherwise, stream 15 is sent to the multi-stage membrane system for CO_2 separation.

We set the hydraulic retention time (HRT) to 20 days based on experimental work by Zhang et al. [14], who report that 87-95% of 30-day biogas yields of FW and DM mixtures can be achieved in a mesophilic reactor in that time. The total solids content of the influent to the digester typical for continuously stirred tank reactors (CSTRs) is around 8 wt% [15]. Other anaerobic digestion reactors, such as covered lagoons, plug-flow digesters, and dry digesters, may have lower capital costs, but CSTRs (also known as Complete Mix Digesters) are common, take up less land than lagoons and plug-flow digesters, are generally easier to clean, and maintain, and are compatible with co-digestion [15]. As such, we employ CSTRs with a maximum volume of 2000 m^3 with a 10 m height; this is the same height as, but nearly five times smaller (in volume) than tanks employed by East Bay Municipal Utility District and about 2-4 times larger than tanks at the UC Davis READ project [16], [17]. Due to the corrosive nature of waste treatment, the tanks should be glass-lined

or constructed from stainless steel. Glass-lined tanks must be carefully built to prevent glass fracture during the installation of tank panels; here, we assume the digesters are made of stainless steel [16].

We assume first-order FW and DM degradation kinetics and determine that the digestion of 50% FW at the base case will require fifteen 2000 m^3 continuously stirred tanks; we include one spare to allow for maintenance without a significant drop in production. The apparent density for FW, 240 kg/m^3, represents restaurant food waste measured in a 1997 EPA report [18]. This value is used to size the storage tank for FW. For DM and DM/FW/water mixtures, the apparent density was calculated utilizing correlations from Wang based on the average total solids (TS) percentage [19]. These densities were used to calculate the required number of AD tanks based on our 20-day HRT and 2000m^3 max volume.

Under these conditions, the organic load rate (OLR) is approximately 2-3 g VS/L/day. The cumulative methane yield and the laboratory analyses of FW and DM determine many other aspects of the digestion process, such as the biogas composition (including CO_2, H_2S, N_2, O_2, and NH_3 gases), total biogas flowrate and %VS destruction (details in SI Sections 1 and 2).

A centrifugal pump moves digested waste to a vibratory screen and screw press unit (PTS E-16 model Agripress [20]) to remove undigested solid waste from the digestate stream at the discharge solids content listed in the Agripress specification (37 wt% solid). A portion of the liquid fraction is purged and disposed of to prevent the overaccumulation of nitrogen, dead cells, and other salts and nutrients. At the same time, the remaining liquid is recycled with fresh feed in the homogenization tank. The homogenization tank is sized for a residence time of 1 hour, with a stirring power requirement equal to 0.1 kW/m^3 [21]. Because of the high moisture contents in the ground wastes (see SI Section 1), adding fresh makeup water is unnecessary in our process. We assume a liquid disposal fee consistent with values provided from industrial sources ($0.06/gallon, [16]).

We calculated dissolved gas concentrations in the exiting digestate in Aspen Plus using a flash drum module and the ELEC-NRTL equation-of-state, which accounts for deviations from pure component solubilities arising from ionic interactions [49], [50]. We assumed vapor-liquid equilibrium (VLE) at 37 °C and 1.05 bar (see SI Section 5). We also compared VLE concentrations using pure component solubilities in pure water via a Rachford-Rice approach [24]. Differences were found for concentrations of dissolved NH_3, H_2S, and CO_2 (Figure S5.2), while predictions for dissolved CH_4, O_2, and N_2 were very consistent. The exiting digestate has a pH of 7-8 (within the optimal range for AD [25]) and contains almost all the NH_3 generated from the reaction. For easy implementation in Excel, we calculated dissolved gas concentrations using the Rachford-Rice method and adjusted them to Aspen Plus results using linear regression (see SI Figure S5.2).

Following the digestion, the biogas is cleaned and purified: a chiller removes water and a significant fraction of the remaining NH_3, activated carbon beds remove H_2S and other sulfur-containing contaminants, and silica gel packed beds adsorb remaining water and impurities

(Details concerning the chiller, blower, activated carbon beds, silica gel beds, and cooling water utility assumptions can be found in SI Sections 7 – 11,14).

4. Anaerobic digestion kinetics

We stratified wastes by volatile fatty acids (VFAs), proteins, lipids, cellulose, hemicellulose, non-structural carbohydrates, and a component containing Sulphur (from which H_2S is derived) based on experimental analyses (Table S4.1). We did not consider lignin a part of VS in this work because it is not easily digestible. We derived food and dairy waste compositions from the four that also reported digestion kinetics (see below). When a component percentage was not reported, we assumed the value to be zero. For simplicity, we assumed the "Sulphur-containing compound" is diethyl sulfide and contributes 0.5 wt% to the waste – analogous to the elemental Sulphur characterization of FW in San Francisco reported by the IEA [3]. For values reported in this work from Li et al., we assumed FW composition was an average of their reported "kitchen waste" and "fruit and vegetable waste" categories with 55% allotted to the latter, as was characterized by the IEA [1].

Table S4.1: Weight fractions of the volatile solids component of wastes studied

Reference	Waste type	VFA	Proteins	Lipids	Cellulose	Hemicellulose	Non-Structural Carbs	Sulphur compound
[2]	FW	0.098	0.231	0.199	0.165	0.091	0.211	0.005
[5]	FW	-	0.183	0.198	0.614	-	-	0.005
[26]	FW	-	0.283	0.372	0.340	-	-	0.005
[27]	FW	-	0.203	0.139	0.653	-	-	0.005
[8]	DM	0.041	0.171	0.078	0.705	-	-	0.005
[28]/[2]	DM	0.002	0.296	-	0.335	0.265	0.098	0.005
[9]	DM	-	0.162	0.012	0.821	-	-	0.005
[10]	DM	0.041	0.061	0.187	0.111	0.378	0.191	0.005
Average	FW	0.025	0.225	0.227	0.443	0.023	0.053	0.005
Standard Dev.	FW	0.049	0.043	0.101	0.232	0.046	0.106	-
Average	DM	0.021	0.173	0.069	0.493	0.161	0.072	0.005
Standard Dev.	DM	0.023	0.096	0.086	0.328	0.191	0.092	-

We then adopted a model compound to represent each component of the waste (Table S4.2) and converted each model compound to biogas via the Buswell equation[29]:

$$C_cH_hO_oN_nS_s + yH_2O \rightarrow xCH_4 + (c-x)CO_2 + nNH_3 + sH_2S$$

We calculated the heats of reaction from the heats of formation for reactants and products, taken from NIST, at 25 °C, also listed in Table S4.2.

Table S4.2: Model compounds used for each waste component

Component	Name	Chemical Formula	ΔH_{rxn}[1]
VFA	Acetic Acid	$C_2H_4O_2$	-35.4
Proteins	Alanine	$C_3H_7NO_2$	64.9
Lipids	Palmitic Acid	$C_{16}H_{32}O_2$	18.8
Cellulose	Alpha-D-Glucose	$C_6H_{12}O_6$	-44.1
Hemicellulose	Alpha-D-Glucose	$C_6H_{12}O_6$	-44.1
Non-Structural Carbohydrates	Alpha-D-Glucose	$C_6H_{12}O_6$	-44.1
Sulphur component	Diethyl sulfide	$C_4H_{10}S$	12.3

[1]Calculated from the Buswell equation in kJ/mol of CH_4 produced

As can be seen from the magnitudes of the ΔH_{rxn}, wastes that contain large amounts of carbohydrates and VFAs are exothermic when digested, while ΔH_{rxn} is endothermic for the digestion of proteins and lipids.

We calculate the conversion of waste (also known as %VS destruction) from the theoretical methane potential (mL CH_4/gVS, assuming 100% conversion for each component) using the Buswell equation and leveraging methane production rates reported for each waste. Because we assume the waste is composed of only model compounds that react with equivalent conversion to give biogas, the %conversion of waste can be estimated from the methane production rate:

$$\%\,conversion\,(X) = \frac{Observed\,methane\,production\,rate(\frac{mL\,CH_4}{gVS})}{Theoretical\,methane\,potential\,(\frac{mL\,CH_4}{gVS})} * 100\%$$

This approach implicitly assumes that all model compounds undergo the same extent of reaction to products. The biogas composition can be computed directly from this approach using the Buswell equation and the average composition of each waste. We assume air introduced to the system from entrained air in the fresh waste or leaks contributes 2.5 mol% to the biogas composition.

We compute biogas production rates using first-order kinetics:

$$B(t) = B_o * (1 - \exp\,(-kt))$$

where B(t) represents the cumulative methane yield (mL CH_4/gVS), B_o is the saturation cumulative methane yield (mL CH_4/gVS), t is digestion time in days, and k is a first-order rate constant derived from literature averages in days^{-1}. Values for B_o for DM and FW are presented in Table S4.3, as well as experimentally measured first-order rate constants (k).

Table S4.3: Biogas compositions from Buswell equation, saturation cumulative methane yield (B_o) and first-order rate constants (k)

			Mole fractions of biogas produced[2]								
Reference	Waste type	ΔH_{rxn}[1]	CH_4	CO_2	NH_3	H_2S	N_2	O_2	X[3]	B_o (mL CH_4/gVS)[4]	k (day^{-1})[5]
[2]	FW	0.64	0.5219	0.3935	0.0599	0.0014	0.0183	0.0049	76.7%	389	0.234
[3]	FW	-3.38	0.5278	0.3980	0.0496	0.0013	0.0183	0.0049	92.3%	462	0.234
[26]	FW	13.57	0.5605	0.3502	0.0650	0.0011	0.0184	0.0049	82.4%	503	0.234
[27]	FW	-6.36	0.5091	0.4107	0.0556	0.0013	0.0183	0.0049	84.1%	392	0.234
[8]	DM	-25.16	0.4770	0.4565	0.0416	0.0017	0.0183	0.0049	37.9%	148	0.34
[12]/[2]	DM	-11.23	0.4456	0.4418	0.0875	0.0019	0.0183	0.0049	45.1%	170	0.34
[9]	DM	-23.78	0.4681	0.4562	0.0510	0.0015	0.0183	0.0049	60.7%	233	0.19
[10]	DM	-13.43	0.5438	0.4128	0.0186	0.0016	0.0183	0.0049	42.1%	209	0.1

[1]Calculated from the Buswell equation in kJ/mol of CH_4 produced for waste mixture
[2]Calculated from Buswell equation, reported waste composition, and model compounds
[3]Calculated assuming a residence time of 20 days
[4]reported in reference
[5]for all FW, the first-order rate constant comes from Browne et al., for DM the reported value from reference is used

We calculated %VS destruction to be nearly double for FW than for DM, consistent with experimental observations from the references surveyed (Table S4.3). Biogas compositions are all within known ranges[30]. Taken together, we estimated biogas production rates and heats of reaction based on input waste flowrates and % FW composition. The variability in B_o for DM and FW is 20% and 30%, respectively, and we used this range in a sensitivity analysis on biogas production.

5. AD VLE and solubility calculations

We determined the dissolved gas concentrations in the exiting digestate of the AD in two ways. First, we performed a simple Rachford-Rice type flash calculation using ideal solubilities for K-values [24]. For a more accurate estimate, we used the flash drum module in Aspen Plus with the ELECNRTL thermodynamic property method to account for significant deviations in the activity coefficients for dissolved ionic species as a function of pH (Figure S5.1) [22], [23]. The “REAC” stream contains the biogas generated from the anaerobic digestion reaction at a flowrate equal to the reaction rate. The “Waste” stream contains only water equivalent to the moisture content of the fresh waste. The water purge rate was varied until the makeup stream was zero. The results of the simple Rachford-Rice calculation and the Aspen Plus simulation are compared in Figure S5.2. For non-ionizing species, such as O_2, N_2, and CH_4, the Rachford-Rice calculation was consistent with Aspen Plus. However, for species able to form ions, such as CO_2, H_2S, and NH_3, the agreement was poor, especially for NH_3. The presence of bisulfide, sulfide, and carbonates enhances the solubility of NH_3 in solution. We corrected for differences between the Rachford-Rice calculation and the Aspen Plus simulation by linear regression. We implemented the equation in the Excel process model to allow for calculations independent of Aspen Plus.

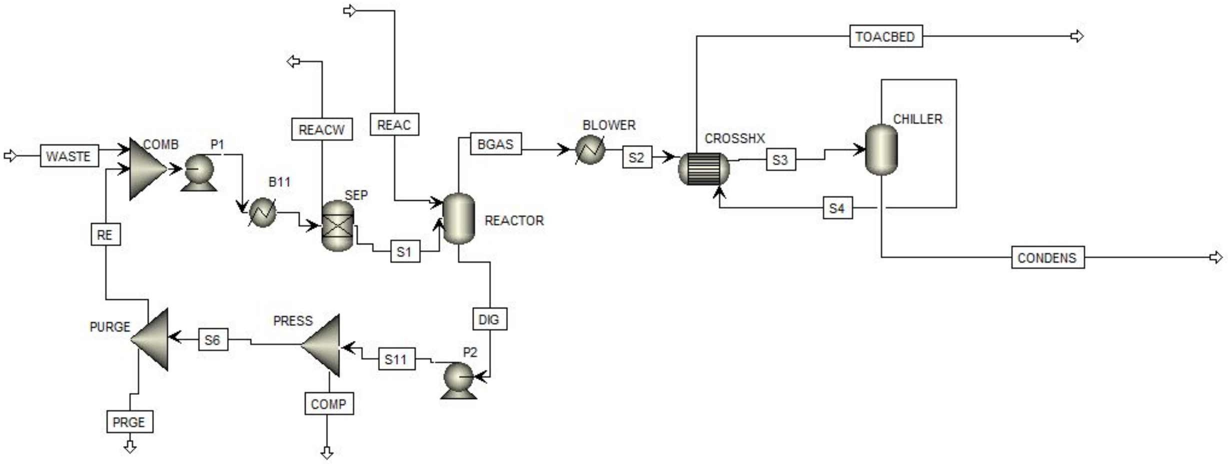

Figure S5.1: Aspen PFD for AD section (including chiller)

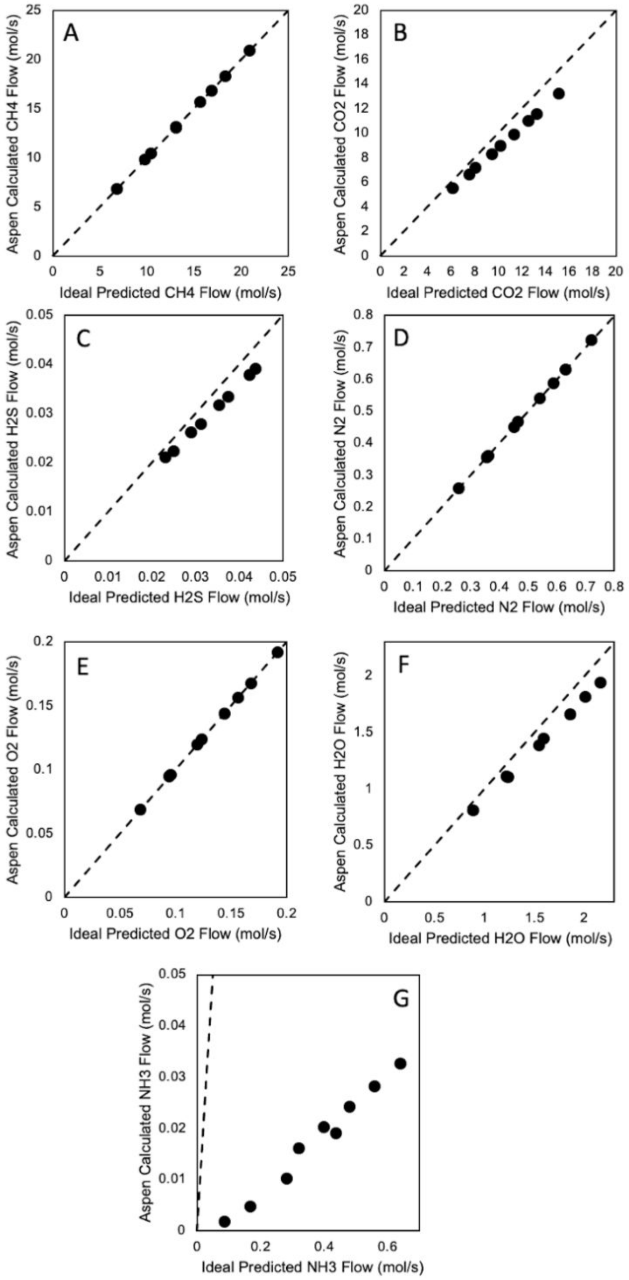

Figure S5.2: Parity plots for all biogas components calculated using Aspen Plus and based on pure component solubilities (Ideal Predicted).

6. Heat transfer analysis for anaerobic digesters

Estimating heat demands for the anaerobic digester (AD) requires an energy balance on the tank. In the absence of detailed AD tank information, we choose to make simplifications to our energy analysis. The work of Hreiz and coworkers loosely inspired the heat transfer model employed here with several major differences [31]. We assume steady state and we define the digesters as insulated stainless-steel cylinders, each containing four isothermal sections: the digestate phase, the biogas phase, the inner wall, and the outer wall (Figure S6.1). We used the one-foot method, a standard used by the American Petroleum Institute [32], to determine the wall thickness. The spray foam insulation thickness was based on the UC Davis READ digester [16].

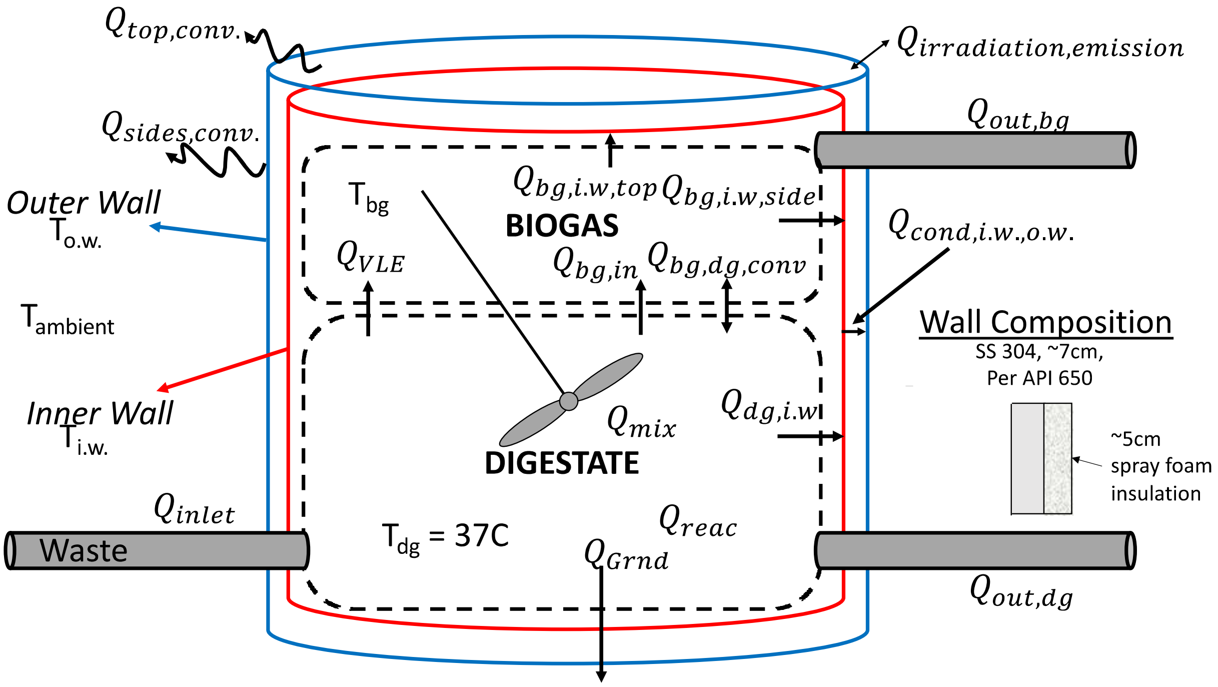

Figure S6.1: Heat transfer zones and phenomena modeled

The steady state energy balances on each section are given below.
Digestate phase:

$$0 = Q_{VLE} + Q_{mix} + Q_{reac} + Q_{dg,i.w.} + Q_{inlet} + Q_{grnd} + Q_{bg,dg,conv} + Q_{out,dg}$$

Biogas phase:

$$0 = -Q_{bg,dg,conv} + Q_{bg,i.w.,top} + Q_{bg,i.w.,side} + Q_{out,bg} + Q_{bg,in}$$

Inner wall:

$$0 = Q_{cond,i.w.,o.w.} - Q_{dg,i.w.} - Q_{bg,i.w.,side} - Q_{bg,i.w.,top}$$

Outer wall:

$$0 = -Q_{cond,i.w.,o.w.} + Q_{sides,conv} + Q_{top,conv} + Q_{emission} + Q_{irradiation}$$

Each term is described in more detail below:

a. Q_{VLE} estimates the heat of evaporation of water and heats of mixing of biogas components with the digestate phase that were calculated using Aspen Plus and the ELECNRTL thermodynamic property package assuming both phases equilibrate at 37 °C.

b. Q_{mix} includes the heat generated from stirring. The EPA has recommended stirring digesters at a rate of 5-8 $\frac{W}{m^3}$, so our base case includes heat effects due to mixing set at 5 $\frac{W}{m^3}$ [33].

c. Q_{reac} includes heat effects from the conversion of food and dairy waste from their base molecules into biogas components (See SI Section 4).

d. $Q_{dg,i.w.}$ is a convection term that describes the heat transfer from the digestate phase to the inner wall. This term is modeled as free convection analogous to a "hot fluid on a cold plate" and takes on the form:

$$Q_{dg,i.w.} = h_{dg,i.w.} * A_{dg,i.w.}\Delta T_{dg,i.w.}$$

Where h, A, and ΔT are the heat transfer coefficients, heat transfer area, and temperature difference of the digestate and inner wall phases. We used a Nusselt number correlation to determine h [34]. We used pure water parameters for the constant pressure heat capacity, dynamic viscosity, and thermal conductivity, which is generally valid for wet AD processes. The density of the fluid was assumed to be 997 $\frac{kg}{m^3}$, assuming the density of digestate does not vary significantly over the range of total solids studied here.

e. Q_{inlet} represents the calculated heat added to the digestate phase by the addition of fresh feed at the pre-heated temperature required to maintain the digestate phase at 37 °C.

$$Q_{inlet} = \dot{m}_{inlet}C_{p,dig}(T_{inlet} - T_{ref})$$

Where $\dot{m}_{inlet}$ represents the inlet mass flowrate of feed (entering through the pipe labeled "Waste" in Figure S6.1) and $C_{p,dig}$ is the constant pressure heat capacity of the inlet waste.

f. Q_{Grnd} represents heat transfer from the digestate phase through the bottom of the tank to the soil below. As a first approximation, this term takes the form:

$$Q_{Grnd} = U_{Grnd} * A_{grnd}(T_{digestate} - T_{Grnd})$$

We assume that T_{Grnd} is equal to the ambient temperature ($T_{ambient}$) and that the overall heat transfer coefficient (U_{Grnd}) is equivalent to literature-estimated values for heat loss of mesophilic wet AD's through 300mm concrete floors in contact with dry earth [35].

g. $Q_{bg,dg,conv}$ represents the heat transfer due to free convection between the biogas phase and the digestate phase. This phenomenon was modeled as a "cold fluid on a hot horizontal plate facing up" heat transfer problem. The functional form for $Q_{bg,dg,conv}$ is equivalent to $Q_{dg,i.w.}$ We used the Nusselt number correlation in ref.[34] on page 300 to determine h.

h. $Q_{bg,i.w.,top}$ represents the heat transfer from the biogas phase to the roof of the digester from free convection and has a form equivalent to $Q_{bg,dg,conv}$ and $Q_{dg,i.w.}$ This term is modeled as a "Cold fluid under a hot horizontal plate" heat transfer problem. The Nusselt number correlation used for determining h is in ref.[34] on page 300.

i. $Q_{bg,i.w.,side}$ represents the heat transfer from free convection due to the biogas phase to the sides of the digester. This term has an equivalent form as $Q_{bg,dg,conv}$ and $Q_{dg,i.w.}$ This term is modelled as a "Cold fluid on a vertical cold plate" heat transfer problem. The Nusselt number correlation used for determining h is in ref.[34] on page 299.

j. $Q_{out,bg}$ represents the heat leaving with the biogas that exits the AD and its form is equivalent to that for Q_{inlet}. For simplicity, we assume the heat capacity of the biogas phase is a mole fraction average of the major components (CH_4 and CO_2).

k. $Q_{bg,in}$ represents the heat entering the biogas phase when the biogas enters the headspace. This form for this term is equivalent to Q_{inlet}. For simplicity, the heat capacity of the biogas phase is assumed to be a mole fraction average of the major components (CH_4 and CO_2).

l. $Q_{cond,i.w.,o.w.}$ represents conductive heat transfer from the inner wall to the outer walls of the digester.

m. $Q_{sides,conv}$ represents the convective heat losses from the outer wall of the digester to the ambient air passing around the digester. This term is modeled as a "flow past a cylinder in cross-flow" heat transfer problem. This term has an equivalent form as $Q_{bg,dg,conv}$ and $Q_{dg,i.w}$ and the Nusselt number correlation used for determining h is in ref.[34] on page 313.

n. $Q_{sides,top}$ represents the convective heat losses of the outer wall of the digester to the ambient air passing on top of the digester. This term is modeled as a "flow past a flat plate" heat transfer problem. This term has an equivalent form as $Q_{bg,dg,conv}$ and $Q_{dg,i.w.}$ The Nusselt number correlation used for determining h is in ref.[34] on page 311.

o. $Q_{emission}$ and $Q_{irradiation}$represents radiative heat loss from the outer wall to the ambient air and heat gain from irradiation of the tank by the sun respectively. These terms were calculated following a similar structure as in Hreiz et al. [31]

The thermodynamic reference temperature (T_{ref}) was set to the mesophilic AD standard temperature of 37 °C. We further assume the digestate phase is isothermal at 37 °C. Therefore, the $Q_{out,dg}$ term is zero at the thermodynamic reference temperature. We solve for the temperatures of each section such that the energy balance equations for each phase are satisfied. To run the model, the inputs required are wind speed, solar irradiance, and ambient temperature. In the Fresno area, these are 5.2 m/s, 5.54 kWh/m^2/day, and 18 °C [36],[37]. The ambient temperature was changed for winter scenarios to the lowest monthly average temperature (8 °C). Hot digestate is recycled to the homogenization tank after solid and liquid waste removal. We assume a temperature loss of 2 °C for the recycled stream before it is mixed with fresh feed in the homogenization tank. We further assume that the fresh feed is equilibrated at the ambient temperature ($T_{ambient}$) before mixing with recycled digestate. We calculate the temperature of the mixed fresh feed and recycled digestate using a standard energy balance and constant heat capacities for both streams. The temperature of this mixture is not high enough to maintain the digestate phase at 37 °C; therefore, heating to T_{inlet} is provided by hot water from the CHP unit.

The temperatures of the inner wall, digestate, and biogas phases are nearly the same at steady state, even during winter conditions (Table S6.1). Generally, we find that the most significant heat demand comes from preheating the feed from the temperature exiting the homogenization tank up to the pre-heat temperature before the digester (Figure S6.2), suggesting that our tank design is sufficiently well-insulated. However, heat loss from the digestate phase in both weather scenarios is dominated by transfer to the inner wall and the ground.

The overall heat requirements (supplied by CHP) ranged from 0.04-0.055 kW/m^2 of digester surface area depending on the waste composition (and at 18°C). For Hreiz et al.'s more rigorous analysis of a semi-buried concrete digester, a value of 0.045 kW/m^2 was calculated [31]. This indicates our calculations are in the correct range.

Table S6.1: Ambient temperature and steady state AD temperatures solved for Fresno average weather conditions and during winter

Variable	Fresno average (base case)	Fresno winter
$T_{ambient}$	18 °C	8 °C
T_{dg}	37 °C	37 °C
T_{bg}	36.8 °C	36.7 °C
$T_{i.w.}$	36.9 °C	36.8 °C
$T_{o.w.}$	20.6 °C	10.9 °C
$T_{inlet.}$	38.2 °C	40.1 °C

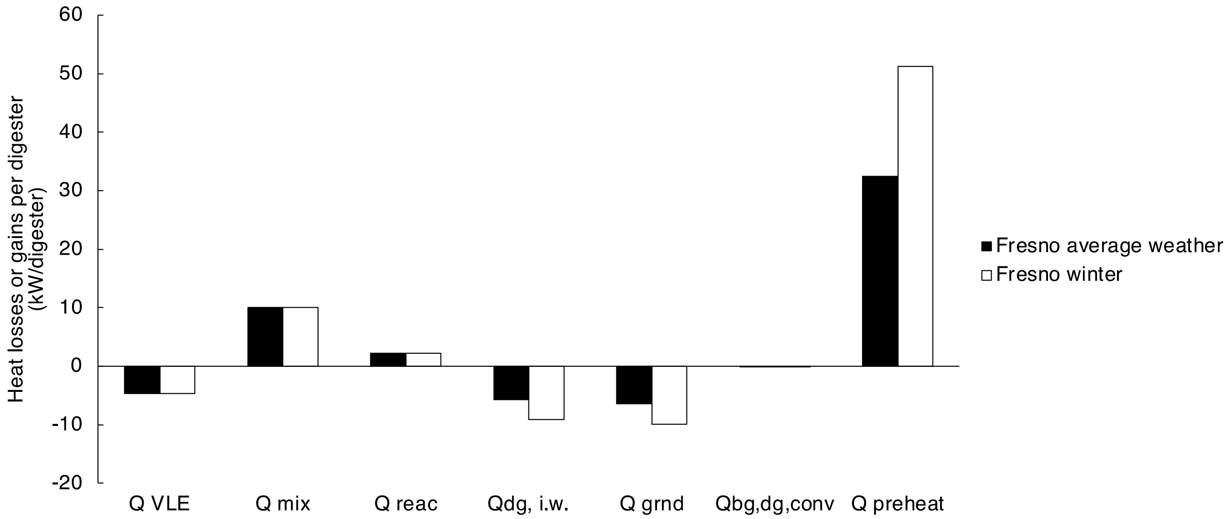

Figure S6.2: Heat losses (negative) and gains (positive) from the digestate phase (Q_{VLE} through $Q_{bg,dg,conv}$) and the heat required to pre-heat the feed from the temperature after the homogenization tank to the preheat temperature to maintain the digestate phase at 37 °C ($Q_{preheat}$)

We assume that most of the heat transfer resistance through the digester wall occurs in the spray-foam insulation layer. As a quick test of that assumption, the heat transfer resistance through the stainless-steel section of the tank can be defined as:

$$R_{Stainless} = \frac{L_{Stainless}}{k_{Stainless}A_{Stainless}}$$

Where L, k, and A is the heat transfer characteristic length, thermal conductivity, and area of heat transfer respectively. For a spray-foam insulation layer 5 cm thick and a standard thermal conductivity of 0.035 $\frac{W}{m^2K}$, and a stainless-steel section 7 cm thick with a thermal conductivity of 15 $\frac{W}{m^2K}$, the ratio of heat transfer resistances is calculated as:

$$\frac{R_{stainless}}{R_{Sprayfoam}} = \frac{L_{Stainless} * k_{Sprayfoam}}{k_{Stainless}L_{Sprayfoam}} = \frac{0.07m * 0.035}{0.05m * 15} = 0.0032 * 100\% = 0.032\%$$

This indicates that the stainless-steel section of the digester contributes <1% to the total heat transfer resistance of the walls, so we only include the spray-foam insulation heat transfer resistance in further modelling efforts.

7. Pump power requirements

Pump power requirements were estimated using standard methods from industry sources, Towler and Sinnott, and SSWL [13], [21], [38].

a. **Pentair Myers submersible grinder pumps, VS50 [13]**
   Submersible grinder pumps are used to grind waste and simultaneously pump it to the anaerobic digester tanks. We assume a head of 45 ft (13.7m) for each pump. According to the specifications listed in the brochure, a flow of 132 gallons (0.5 m^3) per minute (GPM) is achievable at this head for the maximum trim size. At full load, the power draw is 8 kW. If the total flowrate to be pumped exceeds 132 GPM, then the flow is split into two (or more) pumps and the power requirements are scaled proportionally based on the flowrate.

b. **Recirculation pumps for anaerobic digestion recycle**
   Two centrifugal pump sections were used to pump digestate to the screw press and after the screw press to meet with fresh feed to the digester. The head assumed was 15m, which corresponds to a change in liquid pressure of 1.5 bar (assumed to be pure water). Pump efficiency was 67%, and the power calculated by

$$Power = \frac{\Delta P * Flow}{\eta}$$

   Where ΔP is the change in pressure (Pa), the flowrate is in m^3/s and the efficiency is 0.67 [21].

c. **Cooling water pumps**
   We calculated cooling water pump power requirements and costs using the methods outlined in SSLW [38]. The head required was assumed to be 15m, with a corresponding ΔP of 1.5 bar. The SSLW method individually determines the pump efficiency and electrical motor driver efficiency based on flowrate and brake horsepower respectively. The total efficiency calculated was close to 67%, the suggested value in Towler and Sinnott [21].

8. Chiller power and economic calculations

A blower moves the raw biogas from 1.05 bar to 1.5 bar into a chiller unit (Zorg Biogas/Enerdryer [39]) that cools the biogas to roughly 8 °C and removes 85% of the water vapor and 97.5% of the gas-phase NH_3. The biogas stream pre-cools the feed in a heat exchanger to save on refrigeration utility requirements and exits at ~30 °C and 25% relative humidity. We model the chiller unit VLE in Aspen Plus assuming the temperature reaches 8 °C during cooling.

Chiller costs and power requirements are assessed using a combination of publicly available data from Zorg Biogas and Enerdryer, two reputable European biogas equipment suppliers, and calculations in Aspen Plus. Zorg Biogas provides equipment costs for variable biogas flowrates (100-2000 Nm^3/hr) and Enerdryer provides the power consumption of the compressor used in the refrigeration cycle (for system flowrates of 220-1400 Nm^3/hr) in their brochure. This information is plotted in Figures S8.1 and S8.2. Zorg equipment prices do not include the additional cost of a heat recovery cross exchanger (used to pre-cool feed biogas with chilled biogas) and they report chilled biogas outlet temperatures of 10 °C. Enerdryer, however, reports outlet temperatures of near ambient (25 °C), and notes that this system includes a cross-exchanger. Therefore, the power consumption provided by Enerdryer is for a system with a cross-exchanger. We estimate the cross-exchanger cost from the heat exchanger surface area calculated assuming an overall heat transfer coefficient U = 50 W/m^2K [21], typical for gas-gas heat exchangers, and add it to the Zorg biogas cost. The calculations for the Vapor-Liquid Equilibrium of streams entering and exiting the chiller unit (dried biogas and condensate with dissolved gases) were calculated in Aspen Plus.

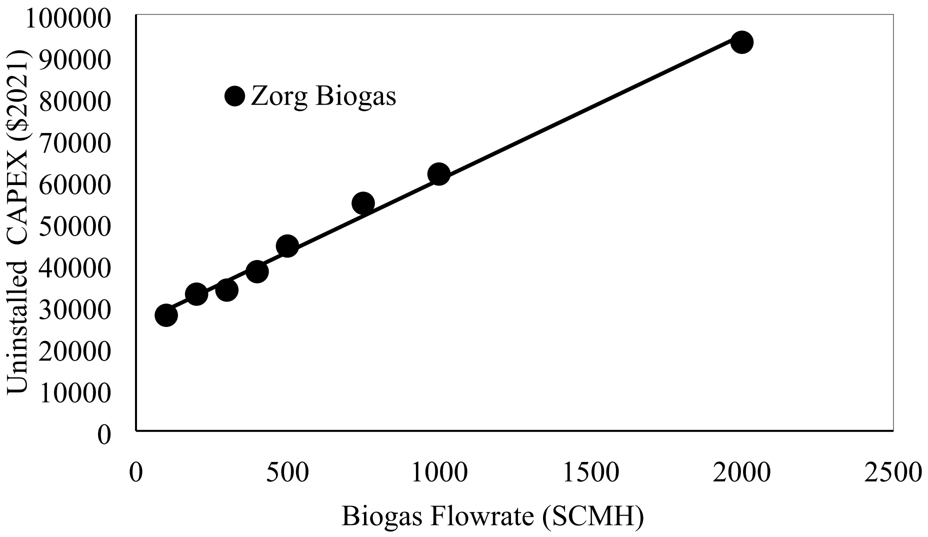

Figure S8.1: Equipment cost for the Zorg biogas chiller vs. biogas throughput

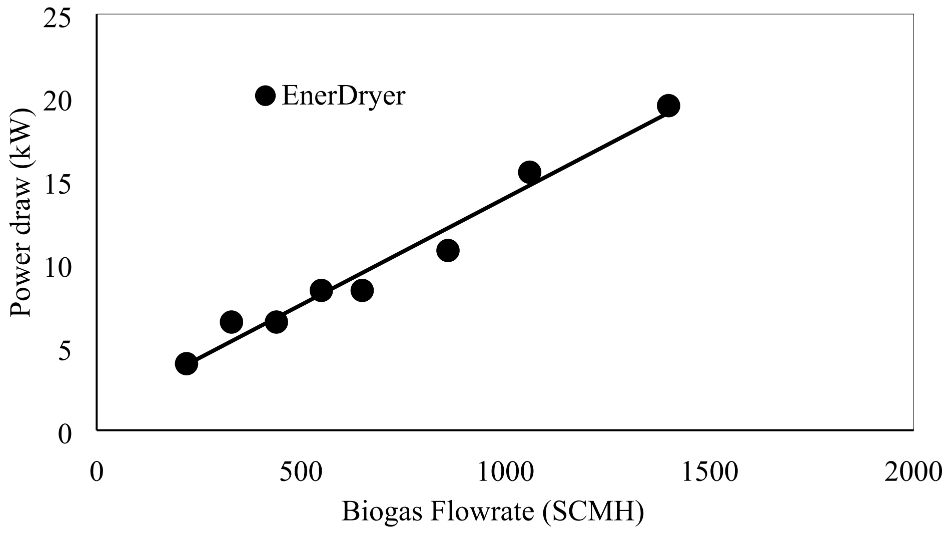

Figure S8.2: Power draw for the gas chiller vs. biogas throughput

9. Gas blower power and economic calculations

We estimated the blower capital cost and power requirements using standard chemical engineering methods in design textbooks [21], [38], supplemented by publicly available data from a reputable European vendor [40]. Blower capital costs are derived directly from their brake horsepower, so we calculate the power required to compress an ideal gas isentropically with a constant heat capacity ratio, using:

$$P_B = 0.00436 * \left(\frac{k}{k-1}\right) * \frac{Q_{inlet}P_{inlet}}{\eta_B}\left(\left(\frac{P_{outlet}}{P_{inlet}}\right)^{\frac{k-1}{k}} - 1\right)$$

Where P_B is the blower brake horsepower, Q_{inlet} is the actual volumetric flowrate (cfm), P_{inlet} is the inlet pressure in psi, P_{outlet} is the outlet pressure in psi, k is the ratio of the ideal gas heat capacities (C_p/C_v), and η_B is the blower efficiency (0.75 is used, as recommended [38]). We convert the brake horsepower to consumed power via an electric motor efficiency factor (η_m) using:

$$\eta_M = 0.80 + 0.0319(\ln(P_B)) - 0.00182 * (\ln(P_B))^2$$

With P_B in horsepower and

$$P_c = \frac{P_B}{\eta_M}$$

Where P_c is the power consumed (hp).

The purchase price of the equipment is determined in two ways. Towler and Sinnott [21] utilize only the volumetric flowrate to make an estimate, while SSLW [38] uses the blower power and provide the choice of a rotary or centrifugal blower. Zorg Biogas provides centrifugal biogas blowers for a range of biogas volumetric flowrates; they report the power consumption for their motors, with average pressure ratios of 1.44. We compare the power estimates over the range of volumetric flowrates available from Zorg Biogas with estimates from either Towler and Sinnott using volumetric flows or SSLW using the pressure ratios of 1.44 for centrifugal blowers (Figure S9.2). Because Zorg blowers are made of aluminum we calculate the purchase cost of the equipment by multiplying the relevant cost of capital equations by a materials factor of 0.6 [38]. Towler and Sinnott overestimate the capex of blowers, likely because they do not consider the size requirements of the motor, which can drive overall blower cost. The predicted power draws using the SSLW methods are quite close to what Zorg Biogas reports for their equipment. Therefore, we utilize SSLW methods for all blower calculations in our analysis.

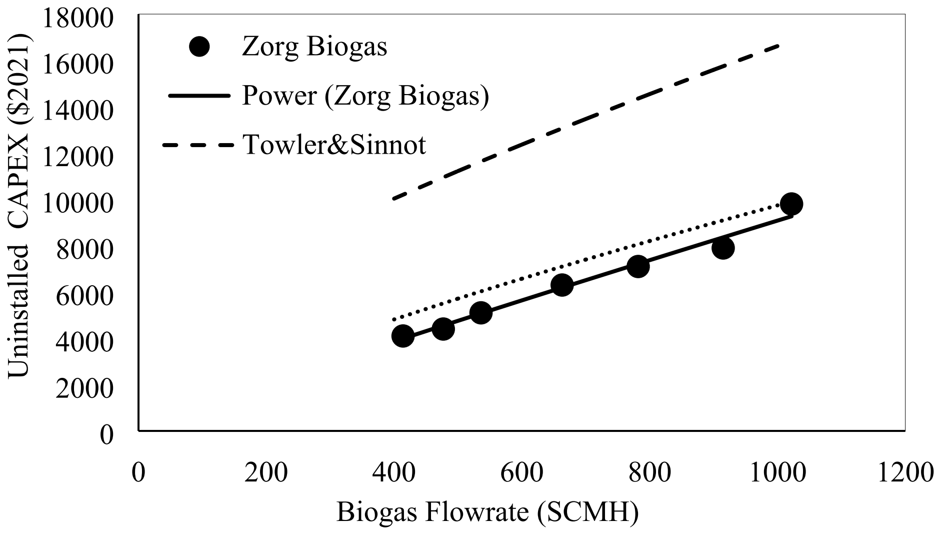

Figure S9.1: Equipment cost for the Zorg biogas blower vs. biogas throughput

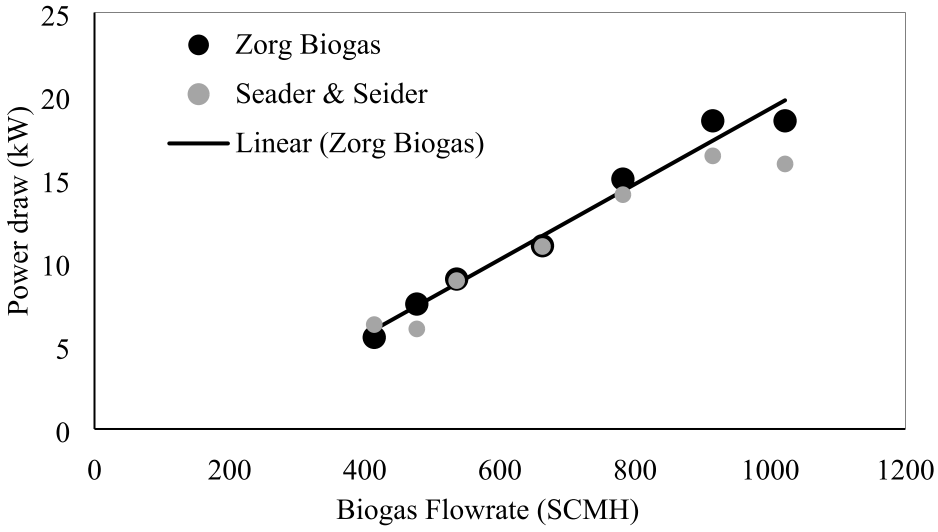

Figure S9.2: Power draw for the gas blower vs. biogas throughput

10. Activated carbon bed calculations

The blower that precedes the chiller unit operates at a pressure ratio of 1.4. This allows the biogas to move from the chiller and through a set of activated carbon beds, assumed to be in a lead-lag configuration to remove H_2S. We utilize the H_2S capacity given in the specifications by General Carbon [41] and model the adsorption column using arithmetic techniques in Towler and Sinnott [21]. We estimate the pressure drop across the bed using the integrated Ergun equation [34]. Our model incorporates periodic removal and replacement of saturated beds every 2 weeks. Other technologies for H_2S scavenging are available, such as the Minicat [42] and Schlumberger Sulfatreat water scrubbing plus iron sponge (used at dry AD plant near San Diego, CA) [43]. Table S10.1 lists assumptions used in modeling the Activated Carbon bed system.

Table S10.1: Bed sizing parameters for Activated Carbon bed

Parameter	Value
Particle size[1]	4mm
Void fraction	0.35
Superficial Velocity	0.2 m/s
Adsorbent Density[1]	500 kg/m^3
Working capacity[1]	0.5 kg H_2S/kg AC
Length/Diameter	1.5

[1]Given by General Carbon specifications

11. Silica gel bed calculations

A blower carries the H_2S scrubbed biogas through a small silica gel Temperature Swing Adsorption (TSA) unit to remove most of the remaining water. We utilize experimental adsorption isotherms for water on type 3A silica gel from Ng et al. [44] and model the system using arithmetic methods from Towler and Sinnott [21]. Hot water from the CHP unit supplies heat for regeneration at 90 °C [45], with a working capacity ~95% of the saturated isotherm value as shown by Ng et al. We estimate the pressure drop across the bed using the integrated form of the Ergun equation [34]. We further assume that the bed active region is only 90% of the total adsorbent to account for the mass transfer-induced adsorption front on the resulting breakthrough curves [21].
Table S11.1 lists assumptions used in modeling the silica gel bed system.

Table S11.1: Bed sizing parameters for silica gel bed

Parameter	Value
Particle size	5mm
Void fraction	0.35
Superficial Velocity	0.2 m/s
Adsorbent Density	730 kg/m^3
Working capacity[1]	0.121 kg H_2O/kg SiO_2
Length/Diameter	1.5

[1]calculated from isotherm data in Ng. Et al. [44]

12. Three-stage countercurrent hollow fiber membranes separation: simulations and data regression

In membrane systems for carbon capture, separation is achieved by the faster permeation of CO_2 through the membranes relative to CH_4. The energy requirement is in the form of compression work rather than heat, but pressurization across membranes is integral to the need for ultimately compressing both product streams. The advantages of these systems are their modularity, flexibility, and relatively small footprints. In addition, membrane systems partly separate oxygen from the RNG product stream [46]. Commercially available membrane systems for biogas upgrading applications are available [47].

We model the separation of CO_2 from CH_4 with a counter-current three-stage hollow fiber membrane system in MATLAB using a modified mathematical procedure as described by Chu et al. [48]. Biogas from the silica gel desiccator is first compressed in two-stages to 16 bar and then fed to a first purification stage that uses highly permeable polymethylpentene membranes (Figure S12.1). A CH_4-rich stream (the retentate) exits Stage 1 and enters Stage 2 where it is further purified to 95% CH_4 using polyimide membranes. The CO_2-rich stream (the permeate) exits Stage 1 at 1 bar and is recompressed to 16 bar and further purified to 96% CO_2 in Stage 3, also using polyimide membranes, as in Esposito et al. [49]. The retentate of Stage 3 is recycled back to Stage 1 to improve methane recovery. The total membrane surface area required to process ~2000 Nm^3/hr is about 2500 m^2. Overall, this process consumes 385 kW of power, or 0.20 kWh/Nm^3 biogas feed.

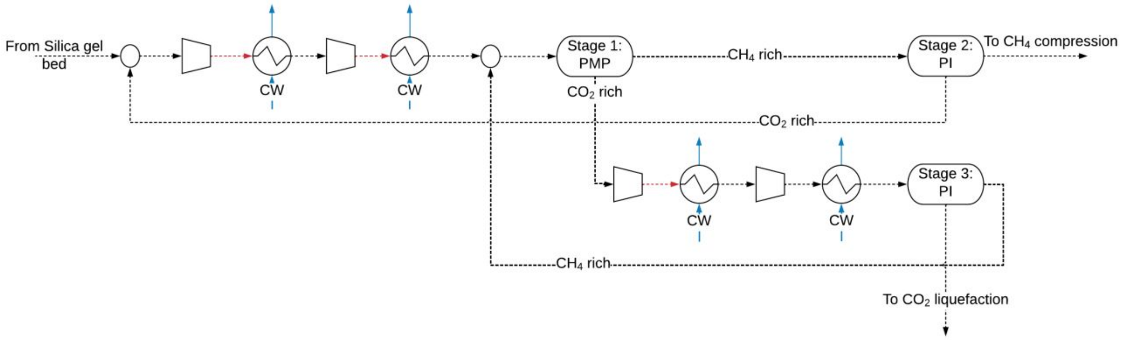

Figure S12.1: Process flow diagram of 3-stage countercurrent hollow fiber membranes carbon capture system utilized in all AD designs (with CCS) in this work.

We wrote MATLAB code to solve the counter-current hollow fiber membrane boundary value problem for a three-stage membrane separation. For the remainder of this section, we define the retentate and the permeate as the streams exiting the membrane on the feed side and the combined stream that is permeating the membrane, respectively. The retentate is always the CH_4-rich stream here. We use many of the mathematical assumptions presented by Chu et al. [48], the most important of which are:

- All streams behave as ideal gases
- Flow across membranes is countercurrent

- No sweep gas is used
- The feed occurs on the shell side
- We neglect edge effects at the inlet and outlet of each module.
- The membranes are isothermal
- Pressure drop is accounted for
- Permeabilities are constant

Additionally, we assume laminar flow in the shell (feed/retentate) and bore (permeate) side of each membrane and assume a square-pitched packing configuration for the hollow fibers within the membrane module [50]. We account for pressure drop using the Hagen-Poiseuille expression [34].

The equations governing the mass transfer across the membrane boundary are presented in detail by Chu et al. [48] and more fundamental details can be found in standard separations texts [24]. In our approach, we adopt a simple shooting method to solve the boundary value problem [51]. The shooting method requires accurate initial guesses that are close to the actual solution to converge; we used an analytical crossflow model as introduced by Coker et al. to furnish reasonable initial guesses [52].

Before modeling the three-stage cascade system (Figure S12.1), we simulated a single membrane stage using equivalent specific parameters for membrane geometry, feed conditions, and permeance values as in "Scenario 1" and "Scenario 3" reported in Chu et al. [48] to validate our methodology (Table S12.1). The major difference between Scenarios 1 and 3 is the higher packing density of hollow fibers in Scenario 3, which influences the pressure drop on the shell side significantly. The agreement between our simulation approach and Chu et al.'s is excellent for both scenarios. Because Chu et al. [48] compared their simulation approach directly with experimental values and found errors less than 3% for permeate and retentate flows and purities, we presume our simulation is also well-validated against experiments.

Table S12.1: Comparison between simulated results for a single membrane in this work and Chu et al.

Source[1]	Permeate Flowrate (mol/s)	Permeate purity (% CO_2)	Retentate Flowrate (mol/s)	Retentate Purity (% CH_4)
Chu et al. – "Scenario 1"	0.0298	59.54	0.3202	94.60
This work – Scenario 1	0.0298	59.8	0.3202	94.64
Chu et al. – "Scenario 3"	0.0181	56.04	0.3319	92.52
This work – Scenario 3	0.0184	56.13	0.3316	92.57

[1]Specific geometrical assumptions for the membrane and feed conditions can be found in Table 2 of Chu et al.

We adopt the more complex three-stage system from Esposito et al. [49]. Table S12.2 shows the geometrical assumptions and permeabilities, with corresponding sources, for the membrane modules.

Table S12.2: Geometrical assumptions for membrane modules and permeabilities of membranes employed

Feature	Value	Reference
**Number of Fibers**	80000	[33]
**Length of Fibers**	0.6 m	[33]
**Outer Fiber Diameter**	250 micrometers	[33]
**Inner Fiber Diameter**	200 micrometers	[33]
**Membrane Thickness**	$1*10^{-7}$ m	[33]
**Inner Diameter of Module**	0.1 m	[33],[47]
**Packing Density**	6400 m^2/m^3	[24]
**Packing Ratio**	0.5	[50]
**Permeabilities (barrer)**[1]		[53]
CH_4 - Polyimide	0.25	
CO_2 - Polyimide	10.7	
CH_4 – Polymethylpentene	14.9	
CO_2 - Polymethylpentene	84.6	

[1]values below for unbolded entries are within the bolded reference

An example output displaying the CH_4 and CO_2 mole fractions in the retentate, as well as pressure drop along the shell side (retentate, CH_4-rich) and bore side (permeate, CO_2-rich) is shown in Figure S12.1 for the first membrane stage (polymethylpentene) for the base case scenario (50% FW, 40 dry kton waste per year). We determined the power requirements for all compressors in the membranes system assuming isentropic compression with an efficiency of 85% [21].

In this analysis, we neglect any temperature effects due to Joule-Thompson (JT) expansion. For a pure stream of CO_2, at 35 °C, the JT coefficient is about 10 K/MPa [54]. As seen in Figure S12.1, the pressure drop is greatest, ΔP = 2.2 bar, or 0.12MPa along the length of the module, in the CO_2-rich bore side. The temperature change associated with an isenthalpic expansion of pure CO_2 with this ΔP is:

$$(0.12\ MPa) * \left(\frac{10K}{MPa}\right) = 1.2K$$

As this is about 0.4% of the assumed temperature, 308.15 K we justify neglecting temperature effects.

We generated multiple linear regression functions against our MATLAB-simulated data. We require that the CH_4-rich stream exits the three-stage cascade at a purity of 95% to meet CNG specifications for transportation fuel [55]. We simulated feed flowrates ranging from 12 to 85 mol/s and purities from 52 to 61.6% CH_4. Outputs for the regression model include compressor power requirements, intercooler areas, cooling water flowrates, number of modules required and total membrane area (Figure S12.3). In all cases, the multiple linear regression model was acceptable to describe simulated data. The fitting parameters can be found in Table S12.3.

Multiple linear regressions against simulated data take on the form:

$$Predicted\ value = C_1 + C_2 * Inlet\ total\ flowrate\ \left(\frac{mol}{s}\right) + C_3 * Inlet\ CH_4\ Purity\ (x_{CH_4})$$

Where C's are fit parameters. Except for the predicted CH_4-Rich stream flowrate. We found that the following form performed better for predicting the CH_4-rich stream flow rate and thus use it as our regression model:

$$Predicted\ value = C_1 + C_2 * Inlet\ CH_4\ flowrate\left(\frac{mol}{s}\right) + C_3 * Inlet\ CH_4\ Purity\ (x_{CH_4})$$

We utilized the regression functions in our Excel anaerobic digestion facility model to greatly simplify further calculations.

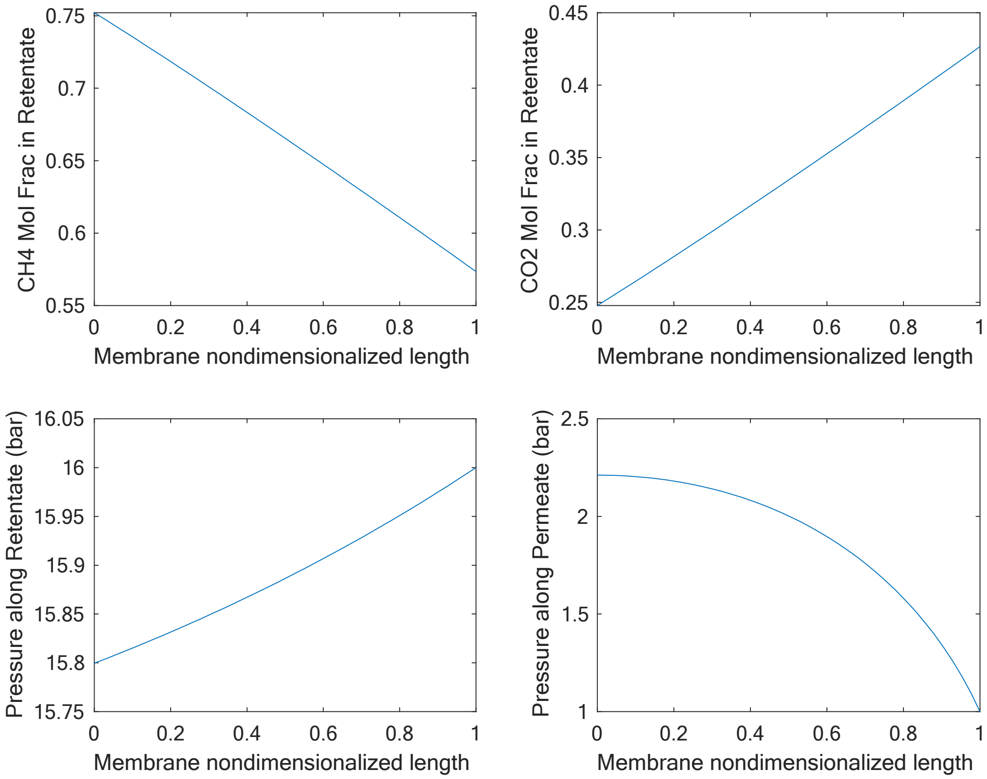

Figure S12.2: Pressure and mole fraction profiles along the length of the polymethylpentene (stage 1) membrane employed in the 3-stage cascade for the base case (50% FW, 40 dry kton waste per year). The membrane geometry was nondimensionalized as in Chu et al. – the non-dimensionalized length of 1 corresponds to the feed inlet (feed on shell side) and the outlet of the CO_2 rich stream (bore side).

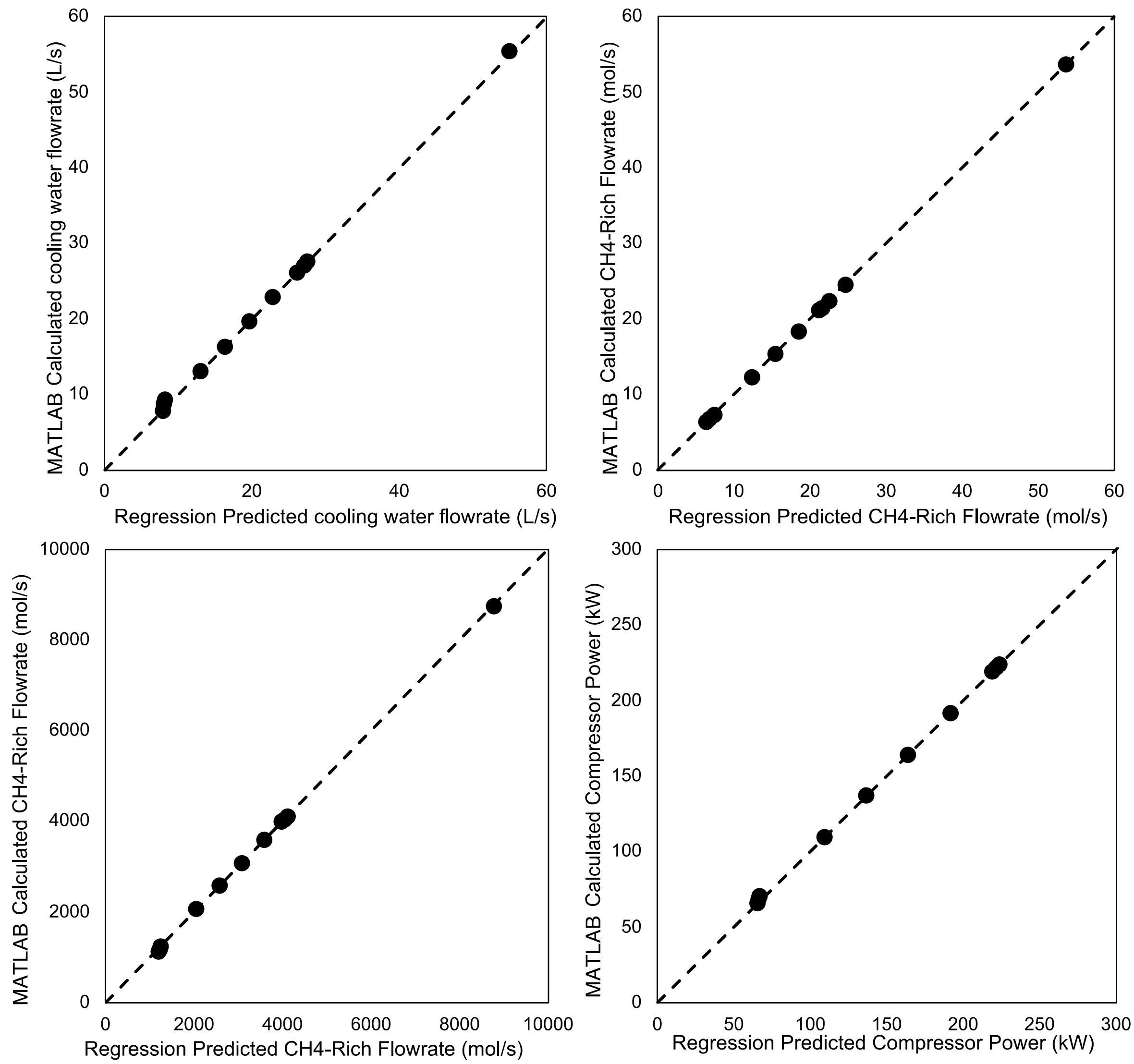

Figure S12.3: Parity plots comparing MATLAB simulation results for the 3-stage membranes system and the multiple linear regression formulae employed in Excel

Table S12.3: Fitting parameters for multiple linear regression functions used to fit simulated data for 3-stage membranes system

Parameter	C1	C2	C3	Units
Stage 1 power	35.07	5.478	-58.51	kW
Stage 2 power	89.50	2.430	-149.2	kW
Total membrane area	-908.1	102.6	1512	m^2
Total cooling water flow	10.63	0.6555	-17.73	L/s
Intercooler 1 area	7.908	1.055	-13.20	m^2
Intercooler 2 area	16.87	0.4577	-28.11	m^2
RNG flowrate[1]	0.0000	-0.0265	1.067	mol/s
CO_2 purity	0.9976	0.0000	-0.0683	-

[1]independent variables are CH_4 inlet flowrate and CH_4 inlet purity

We calculate the total CAPEX for the membranes system to be $3.75MM for a raw biogas production rate of 2150 Nm^3/hr (true for the 50% FW design producing CNG or electricity). Nearly 94% of the CAPEX comes from the multi-stage screw compressor equipment. The biogas upgrading cost correlation from Smith and Scown [56], originally derived from Ong [30] and Urban [57] for PSA, water scrubbing, and amine scrubbing biogas upgrading systems, predicts a CAPEX of $3.8MM while Parker et al. [58] predict a total CAPEX of $7.9MM at this scale. Bauer et al. estimate a cost of $5-$6.5 MM for any biogas upgrading technology "in their most basic form" at this flowrate based on a survey of manufacturers in the EU [59]. Thus, our estimates are within reported ranges for comparable systems and scales. Additional economic information is shown in section 18 of the SI.

Power requirements and performance determined from our simulations are comparable to other literature references (Table S12.4) that employ similar designs.

Table S12.4: Comparison of 3-stage membranes system modeled in this work vs. literature

	**Total power required[1]**	**Purity[2] (CH4/CO2)**	**Recovery (CH4/CO2)**	**Notes**
**Thermodynamic minimum work for separation**	**0.069**	**95%/96%**	**97.5%**	**60% CH4; Outlet CH4 16 bar; Outlet CO2 1 bar**
**This study (PMP, PI)**	**0.20**	**95%/96%**	**~97%**	**Three-stage system**
[30]	0.18-0.35			Review on biogas upgrading technologies
[47]	<0.20	up to 99%/N.R	>98%/N.R.	Sepuran® Green, recovery represents the lower bound for 2-stage process
[49]	0.27-0.29	96%/98%	~99%/94%	Recoveries estimated
[59]	0.20-0.30	98%/N.R.	~99%/N.R.	Outlet pressures unknown, CH_4 recovery is for a 3-stage design

[1]All in kWh/Nm^3 biogas feed normalized using isentropic compression (85% efficiency) to biogas feed at 1 bar, CH_4 product stream 16 bar, CO_2 product stream at 1 bar; purities and recoveries were calculated from mass balances if not reported directly
[2]N.R. = Not reported by reference

13. Transportation of CNG and liquefied CO_2: cost assumptions

We derived the OPEX for liquified CO_2 tube trucks and CNG trucks from Baker et al. [60]. We acquired the CAPEX for CNG trucks from personal correspondence with a sales representative at ANGI ENERGY [61]. An interview between the authors and an industrial representative from Husky Energy provided the CAPEX for liquified CO_2 tube trucks [62]. The road distance between Fresno and Mendota, CA is about 30 miles, so we use this as our liquefied CO_2 transport distance. As for CNG, there are multiple CNG fueling stations all within the greater Fresno area in the nearby towns of Reedley and Caruthers, CA [63]. Since these are all similar in distance from Fresno as Mendota, CA we also assume a 30-mile transport distance for CNG. Table S13.1 summarizes the cost assumptions used in this work.

Table S13.1: Cost assumptions for truck transport of CNG and liquid CO_2

Parameter	Value	Units	Source
Truck operation	1.93	$/truck-mile	[60]
Truck fuel	0.88	$/truck-mile	[60]
Liquefied CO_2 tube truck CAPEX	500,000	$/truck	[62]
CNG tube truck CAPEX	560,000	$/truck	[61]

14. Cooling water temperature and makeup

In the Fresno area, the highest average wet bulb temperature is 18.3 °C during the summer [37]. Assuming a 3 °C approach temperature for the cooling tower, cooling water is available at 21.3 °C during the summer, and this will be lower at other times of the year. We restrict the cooling water return temperature to $\leq$ 40 °C to limit the size of the cooling tower. We assume that every 5.5 °C of cooling in the tower results in 1% evaporative losses. This assumption comes directly from the ratio of the enthalpy of vaporization of water (2,260 kJ/kg) and the heat capacity of water (4.182 kJ/kg/°C) and implicitly assumes the dominant mode of heat transfer is through evaporative cooling. From the cooling water flowrates calculated for each exchanger in our facility, we estimate the water makeup to be 3.4% of the total recirculating cooling water in the facility. We note this result comes directly from the ambient wet bulb temperature, the assumed approach temperature, the assumed return temperature, and the assumption of dominant evaporative cooling. Therefore, the 3.4% makeup stream extends to all other modeled systems in this work regardless of required cooling water flowrates.

15. CO_2 liquefaction

Baker et al. and correspondence between the authors and Husky Energy led us to choose CO_2 liquefaction over compression to bring the CO_2 to approximately –30 °C and 17 bar, standard for truck transportation. [60], [62]. The trailer trucks used for storage and transportation are vacuum layer insulated, with small leak-off rates (<0.1% per day) to keep the CO_2 in the liquid state [62]. The CO_2 trucks can transport 22 tons of CO_2[60], [61].

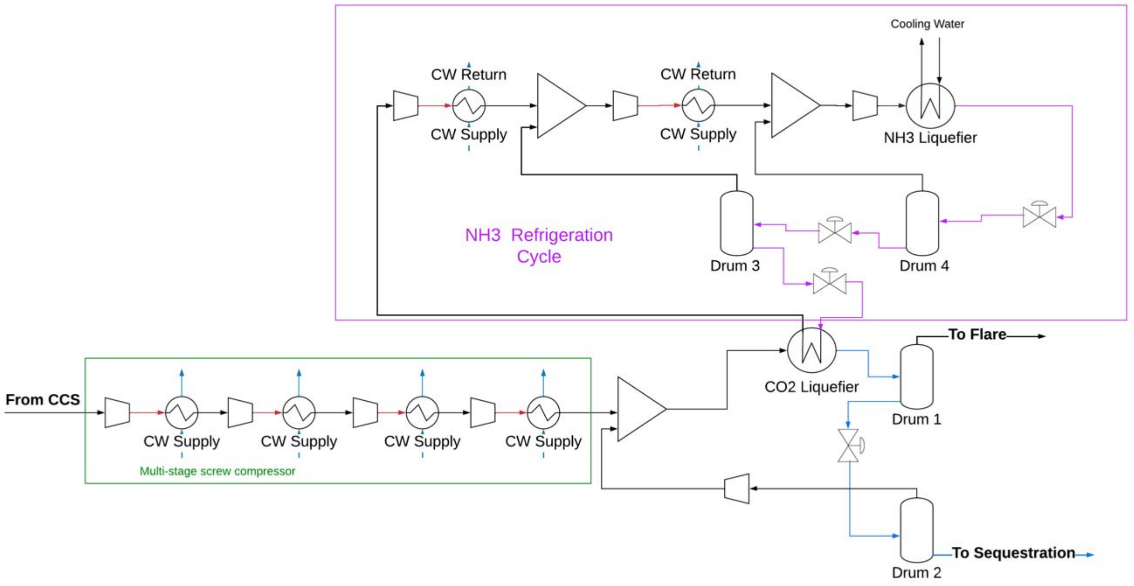

Figure S15.1: PFD for the CO_2 liquefaction process using and R-717 (NH_3) refrigeration cycle

We modeled the CO_2 liquefaction process shown in Figure S15.1 in Aspen Plus using the REFPROP thermodynamic property method with isentropic compressor efficiencies set at 80% [64]. The process uses an ammonia (R-717) refrigeration cycle to liquefy CO_2 at high pressure, like the process described by Jackson and Deng et al. [65], [66]. The CO_2 is first compressed in a multi-stage screw compressor with water intercooling from 1 bar to 21 bar. The compressed CO_2 stream is mixed with a CO_2 recycle stream and pumped to the CO_2 liquefier. The CO_2 liquefier is modeled in three zones, each with overall heat transfer coefficients that depend on the phases present. In the first zone, CO_2 and ammonia are both vapors, and the overall heat transfer coefficient assumed is 25 W/m^{2}K. In the 2nd zone, CO_2 is condensing and ammonia is boiling, which permits the use of a much higher overall heat transfer coefficient of 700 W/m^{2}K. In the final zone where CO_2 is a liquid and NH_3 is boiling, the overall heat transfer coefficient is assumed to be also 700 W/m^{2}K [21]. Liquefied CO_2 exiting at -27 °C and 21 bar is sent to a knockout drum ("Drum 1") to release any non-condensable impurities. We flare the gas stream, while the liquid is sent across a valve to lower the pressure to 17 bar and Joule-Thompson cooling lowers the temperature to -30 °C. The liquid is allowed to equilibrate in "Drum 2" and we recycle the gas stream to the liquefier, while the liquid is sent to CO_2 tube truck trailers for storage, transport, and sequestration.

We assume the refrigeration cycle uses pure R-717. The ammonia exits the CO_2 liquefier as a vapor at 1 bar and is compressed to 2.6 bar in the first stage. We mix recycled ammonia vapor with this stream and compress it further to 6 bar. We mix again and compress to 14 bar. We model the ammonia liquefier in three zones, analogous to the CO_2 liquefier. The cooling utility is facility cooling water (see SI Section 14) that remains in a liquid phase. The ammonia condenses from vapor to liquid. The three overall heat transfer coefficients used are 150, 700, and 250 W/m^2K for the three zones respectively [21]. A series of valves lower the liquid ammonia temperature to -32 °C. We separate the gas released in "Drum 3" and "Drum 4" and recycle it back into the refrigeration cycle.

Similarly, as for the other units, we ran the Aspen Plus model with a selection of input conditions and then used linear regressions of the outputs to model the process easily in Excel. The purity of the incoming CO_2 stream from the carbon capture units is not the same across all scenarios. Therefore, we varied the CO_2 stream composition from 90% to 97.5% CO_2 (with the remainder of the biogas stream CH_4) and applied linear regression to predict the simulation outputs at other CO_2 purities within this range. The CO_2 compressor train properties did not differ significantly across the range of CO_2 purities, so we scaled the outputs linearly assuming 95% CO_2 at 1 mol/s based on the input flowrate for other scenarios (Table S15.1). For example, the function used for scaling the stage 1 CO_2 compressor power is:

$$CO_2\ Stage\ 1\ Power\ (kW) = (Inlet\ CO_2\ Rich\ stream\ flowrate\ from\ CCS) * 2.66\ kW$$

This function is repeated for all stages to determine the power requirements, intercooler heat duties, intercooler heat transfer areas, and CW flowrates for the CO_2 compressor train only.

Table S15.1: CO_2 Compressor train simulation results for a 1 mol/s feed and 95% purity

Parameter	Value	Units
Inlet flow	1	mol/s
xCH_4	0.05	
xCO_2	0.95	
Initial pressure	1	bar
Inlet temperature	35	°C
**Stage 1**		
Power	2.66	kW
Outlet T	103.45	°C
Outlet P	2.15	bar
Heat duty	-2.70	kW
Overall U	150.00	W/m^2K
LMDT	25.78	°C
Area	0.70	m^2
CW Flow	1.97	mol/s
**Stage 2**		
Power	2.65	kW
Outlet T	103.55	°C
Outlet P	4.62	bar
Heat duty	-2.74	kW
Overall U	150.00	W/m^2K
LMDT	25.81	°C
Area	0.71	m^2

CW Flow	2.00	mol/s
**Stage 3**		
Power	2.62	kW
Outlet T	103.75	°C
Outlet P	9.93	bar
Heat duty	-2.82	kW
Overall U	150.00	W/m^2K
LMDT	25.88	°C
Area	0.73	m^2
CW Flow	2.06	mol/s
**Stage 4**		
Power	2.55	kW
Outlet T	104.15	°C
Outlet P	21.33	bar
Heat duty	-3.01	kW
Overall U	150.00	W/m^2K
LMDT	26.02	°C
Area	0.77	m^2
CW Flow	2.20	mol/s

Model outputs that depend on CO_2 purity include the ammonia compressor power, intercooler areas, cooling water flowrates, CO_2 and ammonia liquefier areas, ammonia liquefier cooling water flowrates, and drum sizes. For all these except drum sizes, the regression takes the form:

$$Value\ of\ property\ (i.e.\ NH_3\ liquefier\ area) = (Inlet\ CO_2\ Rich\ stream\ flowrate\ from\ CCS) * (x_{CO_2,from\ CCS} * C_1 + C_2)$$

Where C_1 and C_2 are fit parameters across each CO_2 purity simulation for an inlet CO_2 rich stream flowrate of 1 mol/s.

Drum sizes were sized as pressure vessels using standard methods [21] and relevant information from Aspen Plus simulations, such as the vapor and liquid flowrates and densities exiting the drums. For pressure vessel sizing, we assumed A285 carbon steel construction, with a 2mm corrosion allowance, and double welded butt joints (with no radiographic inspection) [21]. Flowrates were scaled linearly (for each purity), and drums were resized appropriately using standard methods [21]. Shell masses were estimated from the size of each drum and correlated to the inlet CO_2 rich stream flowrate and purity from the carbon capture units. In total, we sized 24 drums to sample across 6 CO_2 inlet flowrates (at 90%, 92.5%, 95%, and 97.5% CO_2).

We used multiple linear regression as follows:

$$Predicted\ value = C_1 + C_2 * Inlet\ CO_2\ rich\ flowrate\ from\ CCS\left(\frac{mol}{s}\right) + C_3 * Inlet\ CO_2\ Purity\ (x_{CH_4})$$

The parity plots in Figure S15.2 show that the agreement between this function and the true shell masses are reasonable.

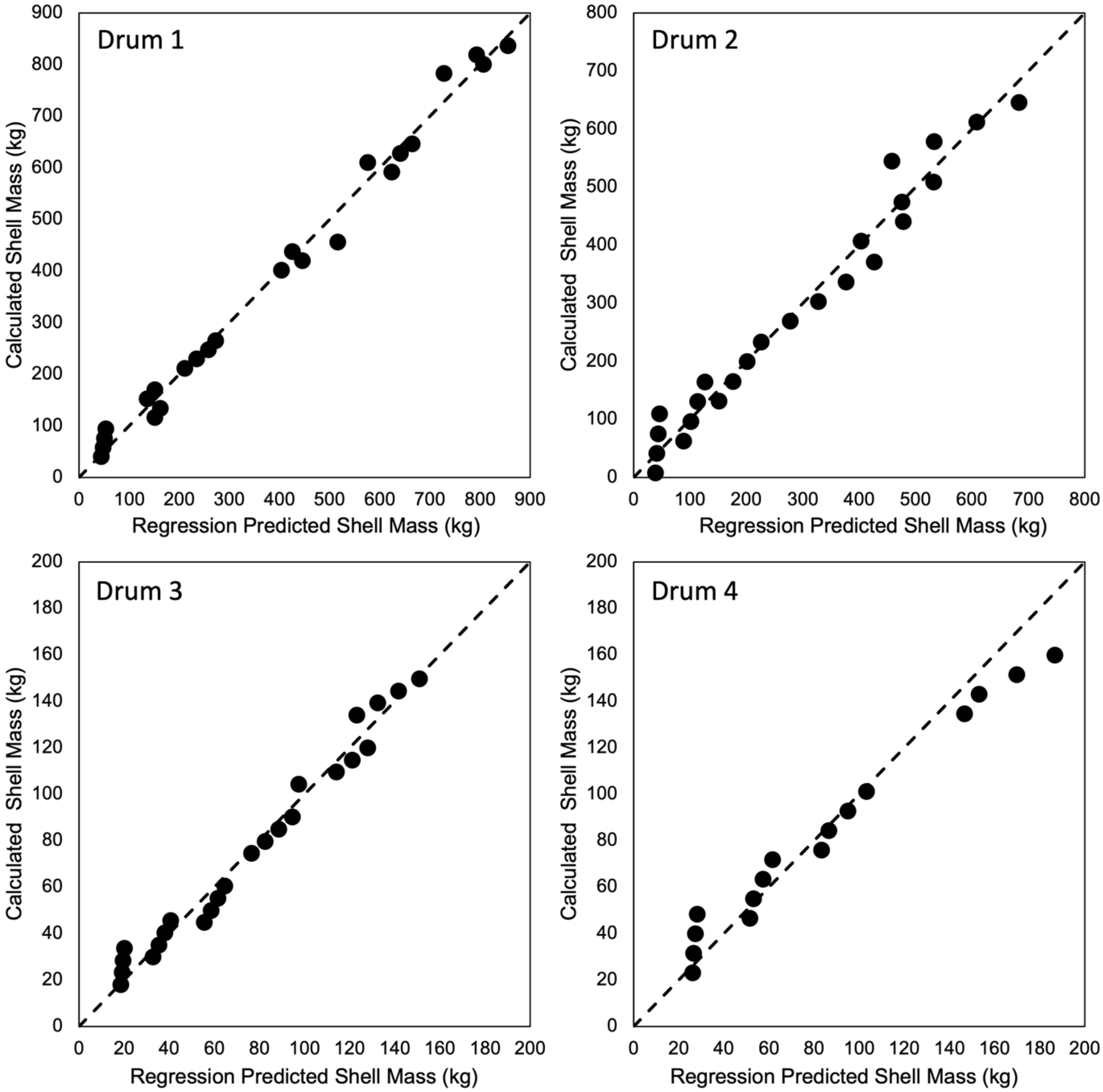

Figure S15.2: Parity plots comparing pressure vessel sizing calculations for the CO_2 liquefaction drums and the multiple linear regression formulae employed in Excel

The liquefaction to CO_2 at –30 °C and 17 bar consumes about 102 kWh/ton of CO_2 product. Jackson et al. report the specific work for this system to be 102 kWh/ton at outlet conditions of –28 °C and 15 bar, while Soo et al. (referenced within Jackson et al.) report a value of 101 kWh/ton for a similar system [70]. Chen et al. performed an energetic and economic analysis on a system that processes nearly 300 times the amount of CO_2 studied here from 2 bar to –28 °C and 15 bar and found the specific energy consumption to be only 69 kWh/ton [67]. However, they used a slightly higher isentropic efficiency assumption (85%) and the additional compression from 1 bar to 17 bar requires nearly 20 kWh/ton, resulting in a comparable total power consumption of 95 kWh/ton.

We calculate that the CO_2 liquefaction unit and vacuum-insulated liquid CO_2 trucks have installed costs of $2.5MM and accommodate 28 tonnes CO_2/day. McKaskle et al. calculated an installed cost of $5.2MM for a 68 tonnes CO_2/day liquefaction process with storage for the Illinois State Geological Survey, which included some off-site infrastructure [68]. Scaling our liquefaction unit to this flowrate and including installation and a 40% additional offsite factor yields a similar estimate of $5.6MM. McCaskle et al. validated their estimates with industrial sources, so we believe our estimates are reasonable [68].

16. CH_4 compression to CNG

The effluent streams from the membrane process are high-purity CH_4 (95 mol%) and CO_2. The CH_4 is further compressed to 283 bar in a three-stage reciprocating compressor with interstage cooling (costs modeled in [21], but validated by vendor ANGI energy, [61]) and stored in CNG tube truck trailers (ANGI energy, [61]). The CNG trucks can transport 450000 SCF of CNG [60], [61].

We conducted CH_4 compression simulations in Aspen Plus utilizing the REFPROP property method and the isentropic compression function with an efficiency of 85% [64]. Because the purity is set by standards for CNG in natural gas vehicles at 95% CH_4 and the pressure of the CH_4 rich streams exiting the carbon capture units are all 16 bar, we only needed to run a single simulation with a basis flowrate (1 mol/s) at 95% CH_4 purity with 5% CO_2 and then use linear scaling to match any given flowrate. Our inputs are the inlet flowrate, composition, pressure, and temperature. We tabulate the outputs in Table S16.1: power requirement for each compressor stage, the outlet temperatures before intercooling, outlet pressures, and the heat duty requirements for each intercooler. We then calculate the log-mean temperature difference (LMTD) and the heat exchanger area based on an assumed heat transfer coefficient of 150 W/m^2K, standard for gas-cooling water heat exchangers [21]. We calculate the cooling water (CW) flowrate as described in SI Section 14. The power, heat duty for the intercoolers, cooling water flowrates, and intercooler areas are all scaled linearly based on the inlet flowrate, which is a function of the scale of the facility and the effectiveness of the carbon capture technology. For example, the function used for scaling the stage 1 CH_4 compressor power is:

$$CH_4\ Stage\ 1\ Power\ (kW) = (Inlet\ CH_4\ Rich\ stream\ flowrate\ from\ CCS) * 3.08\ kW$$

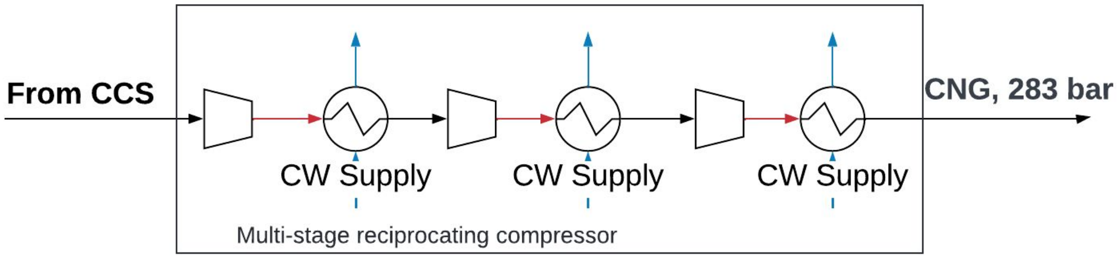

Figure S16.1: PFD of the 3-stage reciprocating compressor with water intercooling to compress high-purity CH_4 into compressed natural gas (CNG)

Table S16.1: CH_4 Compressor train simulation results for a 1 mol/s feed and 95% purity

Parameter	Value	Units
Inlet flow	1	mol/s
xCH_4	0.95	
xCO_2	0.05	
Initial pressure	16	bar
Inlet temperature	35	°C
**Stage 1**		
Power	3.08	kW
Outlet T	114.61	°C
Outlet P	41.69	bar

Heat duty	-3.26	kW
Overall U	150	W/m²K
LMDT	29.58	°C
Area	0.74	m²
CW Flow	2.32	mol/s
**Stage 2**		
Power	3.06	kW
Outlet T	123.64	°C
Outlet P	108.62	bar
Heat duty	-4.12	kW
Overall U	150	W/m²K
LMDT	32.51	°C
Area	0.84	m²
CW Flow	2.93	mol/s
**Stage 3**		
Power	2.97	kW
Outlet T	122.11	°C
Outlet P	283	bar
Heat duty	-4.66	kW
Overall U	150	W/m²K
LMDT	32.03	°C
Area	0.971	m²
CW Flow	3.31	mol/s

When the total compressor power requirement was greater than 75 kW, a reciprocating compressor was assumed and costed as in [21]. Screw compressors are used below this threshold and are costed as in [38].

17. CI score calculations and adjustments

The California Air Resources Board (CARB) provides a database with CI scores for facilities that digest DM or FW in California [69]. One of the largest DM digester developers in CA, California Bioenergy (CalBio), holds 19 pathways to produce Bio-CNG. The average CI score is -325 gCO_2-eq/MJ across this data set, with a moderate standard deviation (61 gCO_2-eq/MJ). The LCFS Tier 2 pathway applications we assessed did not mention an onsite CHP unit. For FW, this table only provides one entry at -79 gCO_2-eq/MJ for the South San Francisco Scavenger Company. A recent application by the Napa Zero Waste Energy (ZWE) facility that performs dry AD of food waste calculated a CI score of –165 gCO_2-eq/MJ utilizing CARB's Tier1 simplified GREET model [70]. ZWE generates 320,000 diesel gallon equivalents of RNG per year and 570 kW of electricity to power onsite operations and sell to the grid. Using a 36% electrical efficiency for the CHP unit, we calculate that ZWE diverts roughly half of its biogas to CHP. Our estimates using the Tier1 simplified GREET model for biomethane production from AD, without accounting for the diversion of biogas to CHP, furnish a value of –87.6 gCO_2-eq/MJ for a 100% FW scenario. However, significant differences in the heat and power demands of the facility necessitate varying degrees of biogas diversion and subsequently different CI scores for the saleable CNG. We adjust the CI score for each waste based on the biogas diversion to CHP using the following expression:

$$CI_{adj} = \frac{CI_{nodiv}}{(1 - X_{div})}$$

where CI_{nodiv} = -87.6 gCO_2-eq/MJ for FW and -325 for DM, X_{div} is the fractional diversion of biogas to CHP, and CI_{adj} is the CHP-adjusted CI score. We do not adjust the CI score by the CO_2 emissions of the CHP unit which are already accounted for in the CI_{nodiv} term because the end use of the CNG is combustion to CO_2 in a natural gas vehicle [71]. Using this methodology for the ZWE facility's biogas diversion, we calculate a $CI_{nodiv,FW}$ of -82.5 gCO_2-eq/MJ, very similar to our calculation using the Tier 1 simplified GREET model assuming no diversion to CHP. We do not form a composite CI score to calculate LCFS credits for mixtures of FW and DM. We simply calculate the LCFS credit generation independently for each waste using the fraction of biogas produced. In the event purchased electricity is used, the CI scores are adjusted using a CA grid CI score of 64.6 gCO_2/MJ of power [71]. If CO_2 sequestration is performed, we adjust the CI score of the CNG produced based on the sequestration rate.

For electricity-producing designs, we use an Energy Equivalence Ratio (EER) of 3.4 to adjust the CI score for each waste *and* apply an engine efficiency adjustment factor as described in a guidance document released by CARB [72], [73]. As above, we do not adjust the CI score by the CO_2 emissions of the reciprocating engine as this is already captured in the CI_{nodiv} term. If CO_2 sequestration is performed, we adjust the CI score of the electricity produced based on the sequestration rate for that design.

18. List of key economic and process variables, purchased costs for major equipment, installation factors, OSBL, D&E, contingency, and total CAPEX for each design

Here, we tabulate (Table S18.1) a list of key economic and process variables used in the base case facility (50% FW).

Table S18.1: List of key economic and process variables used in the base case

Economic variables	Base case values	Units	Reference
Electricity selling price	0.04	\$/kWh	[74]
CNG base price	3.34	\$/MMBTU	[75]
Tipping fee	30.70	\$/ton	[76]
Facility footprint	9	acres	[77]
Solid disposal fee	30.70	\$/ton	[76]
Liquid disposal fee	0.06	\$/gal	[16]
Cooling water	0.32	\$/m^3	[78]
Electricity utility charge	0.16	\$/kWh	[79]
Grid interconnection fee	200,000	\$	[56]
LCFS credit price[1]	110	\$/tonne	[80]
$CI_{standard}$ – Gasoline 2021[2]	90.74	gCO_{2e}/MJ	[81]
D3 RIN (for DM)[3]	3.11	\$/RIN	[82]
D5 RIN (for FW)[3]	1.55	\$/RIN	[82]
45Q tax credit threshold	12.5	ktonne/year	[83]
45Q tax credit value	85	\$/tonne	[83]
CO_2 IMRV[4]	11	\$/tonne	[84]
Inflation Reduction Act investment tax credit	30	% of qualified property	[85]
**Process variables**	**Base case values**	**Units**	**Reference**
Waste input	36	dry ktonne/yr	SI Section 1
Waste composition	50	% FW (wet)	Basis
Total solids in AD (TS)	8	wt%	[15]
Organic load rate (OLR)	2.6	g VS/L/day	Calculated based on feed flow, HRT, and TS concentration
Hydraulic retention time (HRT)	20	days	[86]
AD tank size	2000	m^3	SI Section 3
AD tank height	10	m	SI Section 3
AD stir rate	5	W/m^3	[33]
Liquefied CO_2 tube truck capacity	22	tonnes/truck	[60]
CNG tube truck capacity	450000	SCF/truck	[61]
Liquefied CO_2 tube truck average roundtrip distance to well	30	miles	Road distance between Fresno, CA and Mendota, CA
CNG tube truck average roundtrip distance	30	miles	[63]
FW biogas production rate	850	mL biogas/gVS	Calculated from kinetics (SI section 2)
DM biogas production rate	409	mL biogas/gVS	Calculated from kinetics (SI section 2)

[1]LCFS = Low Carbon Fuel Standard

[2]Carbon Intensity benchmark for gasoline in the year 2021; for simplicity, we assume this value is constant in time

[3]RIN = Renewable identification number as in the Renewable Fuel Standard (RFS)

[4]IMRV = Injection, monitoring, reporting, and verification

Quotes for equipment were used whenever possible. In cases where industrial quotes were not available, we estimated equipment costs via correlations presented in Towler and Sinnott or SSLW [21], [38]. We calculated the yearly land lease by estimating the required footprint of the

facility in acres, rounding up to the nearest integer, from the correlation presented in Angelonidi et al. [77], and taking typical land lease annual costs (I.e., $/acre/year) for the Fresno area from realty sources [87].

Table S18.2 summarizes the reference employed for the base equipment cost, followed by the factors used for installation to form the ISBL cost. Installation costs that are 3.68 include installation and a stainless-steel correction, while 3.2 is for carbon steel. An installation factor of 2.5 corresponds to solids handling installation [21]. We assume costs associated with cooling water pumps and towers are included in the OSBL cost. We further assume the installation costs of adsorbents, solvents, and trucks are zero. We include spares for all pumps. All drums and columns were sized as pressure vessels and costed based on shell mass. All costs are in 2021$ on a West Coast USA basis. We used the Chemical Engineering Plant Cost Index (CEPCI) to scale costs for equipment provided in different years.

Table S18.2: Equipment list, uninstalled costs, installation factors, and calculation of total CAPEX for Biogas to CNG (CCS) design

Equipment	Cost source	Uninstalled cost per unit[1]	# of units	Installation factor	Total installed cost	Notes
**Anaerobic digestion section**						
Anaerobic digesters	[21]	$491,470	16	3.68	$32,700,281.30	Costed as sum of cone roof tank and spiral ribbon mixer
FW storage tank	[21]	$169,949	1	3.68	$625,411	Cone roof tank
DM storage tank	[21]	$52,378	1	3.68	$192,753	Cone roof tank
Homogenization tank	[21]	$51,728	1	3.68	$190,359	Cone roof tank
Homogenization tank agitator	[21]	$49,641	1	3.68	$182,678	spiral ribbon mixer
FW shredder	[12]	$148,018	1	2.5	$370,045	
Grinder pumps	[13]	$7,185	8	3.2	$183,936	
Screw press	[20]	$135,500	3	3.2	$1,300,800	Includes 60” vibratory screen, control panel, and flowmeter
Feed pre-heat heat exchanger	[21]	$45,467	1	3.68	$167,320	U-tube shell and tube
Centrifugal pumps	[21]	$15,449	4	3.68	$227,405	See SI Section 9 for detailed information
**Chiller section**						
Chiller	[39]	$100,047	1	3.2	$320,149	See SI Section 8 for detailed information
Chiller cross exchanger	[21]	$47,169	1	3.2	$150,940	
Chiller gas blowers	[38]	$19,215	11	3.2	$61,489	See SI Section 9 for detailed information
**Activated carbon bed section**						
H_2S adsorption column	[21]	$32,381	1	3.68	$119,163	
Activated carbon adsorbent	Quote – General Carbon	$24,762	1	1	$24,762	$2.54/kg, initial charge of adsorbent
**Silica gel bed section**						
Silica gel adsorption column	[21]	$46,687	1	3.2	$149,399	
Silica gel adsorbent	[88]	$43,147.10	1	1	$43,147.10	$5.40/kg, initial charge of adsorbent

Silica gel gas blower	[38]	$13,927.75	1	3.2	$44,568.81	See SI Section 8 for detailed information
**3-stage membranes section**						
Membranes	[46]	$24,928		3.2	$79,771.05	$10/m^2$, replace every 5 years
Compressor train pre-stage 1	[38]	$374,446.19	1	3.2	$1,198,227.82	Screw type
Compressor train pre-stage 3	[38]	$207,502.64	1	3.2	$664,008.45	Screw type
Compressor train pre-stage 1 intercoolers	[38]	$3,515	2	3.2	$22,494	Double-pipe
Compressor train pre-stage intercoolers	[38]	$3,515	2	3.2	$19,676	Double-pipe
**CNG compression section (Biogas to CNG only)**						
Reciprocating compressor train	[21]	$542,639	1	3.2	$1,766,658	Costed as one compressor with multiple stages, includes water intercoolers. Cost estimation verified with correspondence from ANGI energy
CNG trucks	[61]	$560,000	3	1	$1,680,000	
**CO_2 liquefaction section**						
Liquefied CO_2 trucks	[60]	$500,000	2	1	$1,000,000	
Compressor train for CO_2	[38]	$197,520.80	1	3.2	$632,066.57	Screw type, includes water intercoolers
Compressor train for NH_3 cycle	[38]	$111,312.41	1	3.2	$356,199.72	Screw type, includes water intercoolers
NH_3 liquefier	[21]	$44,589.17	1	3.2	$142,685.35	U-tube shell and tube
CO_2 liquefier	[21]	$49,965.57	1	3.2	$159,889.82	U-tube shell and tube
Drum 1	[21]	$21,645.82	1	3.2	$69,266.61	
Drum 2	[21]	$18,625.00	1	3.2	$59,600.00	
Drum 3	[21]	$22,418.77	1	3.2	$71,740.05	
Drum 4	[21]	$21,645.82	1	3.2	$69,266.61	
**On-site hot water heater**						
Fired heater for hot water	[38]	$124,803	1	2.19	$273,317.99	
Total ISBL Cost					$45,322,099.09	Sum of Installed equipment
OSBL	[21]				$18,128,839.64	OSBL is 40% of ISBL
Design and engineering	[21]				$15,862,734.68	25% of OSBL+ISBL
Contingency	[21]				$6,345,093.87	10% of OSBL+ISBL
Total CAPEX	[21]				$85,658,767.28	

[1]all on a carbon steel basis - the installation factor accounts for stainless-steel correction

After applying the appropriate installation factor for each piece of equipment, the sum of the costs is the total ISBL cost. The OSBL cost was assumed to be 40% of ISBL, Design and Engineering costs were 25% of OSBL+ISBL, and contingency costs were 10% of OSBL+ISBL [21]. The sum of these components furnishes the "Total CAPEX" as presented in Table S18.3 for all designs.

Table S18.3: Total capital expenses (MM$) for wet AD designs considered in this work

Waste composition	Biogas to EV	Biogas to EV (CCS)	Biogas to CNG	Biogas to CNG (CCS)
**0% FW**	74.2	80.8	70.6	74.8

**50% FW**	87.1	95.7	80.8	85.7
**100% FW**	92.6	102.6	83.2	88.5

We generated a simple graphic of the percent contribution of each section to the total CAPEX for the **Biogas to CNG (CCS)** design (Figure S18), clearly demonstrating that CAPEX is dominated by the AD section.

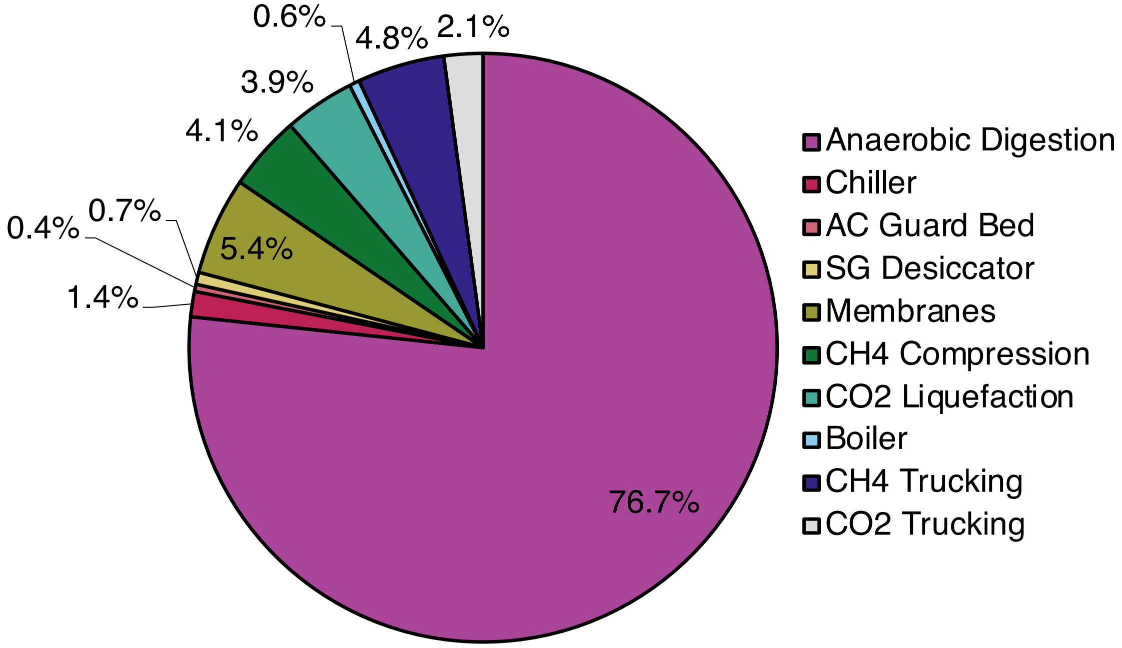

Figure S18: Capital cost breakdown for Biogas to CNG (CCS) wet AD design when digesting 50%FW. AC = activated carbon, SG = silica gel.

The ISBL costs for the CHP unit are displayed in Table S18.4. The reported costs from EPA included OSBL equipment, thus the values in Table S18.4 were calculated by dividing the EPA values by 1.4 (OSBL factor) and correcting 2013$ to 2021$.

Table S18.4: ISBL capital expense (MM$) for reciprocating engine CHP units in this work

Waste composition	Biogas to EV	Biogas to EV (CCS)
**0% FW**	4.85	4.83
**50% FW**	8.94	8.92
**100% FW**	11.87	11.85

19. Revenues and OPEX for all designs, and NPVs for facility designs with 100%FW and 0%FW waste compositions

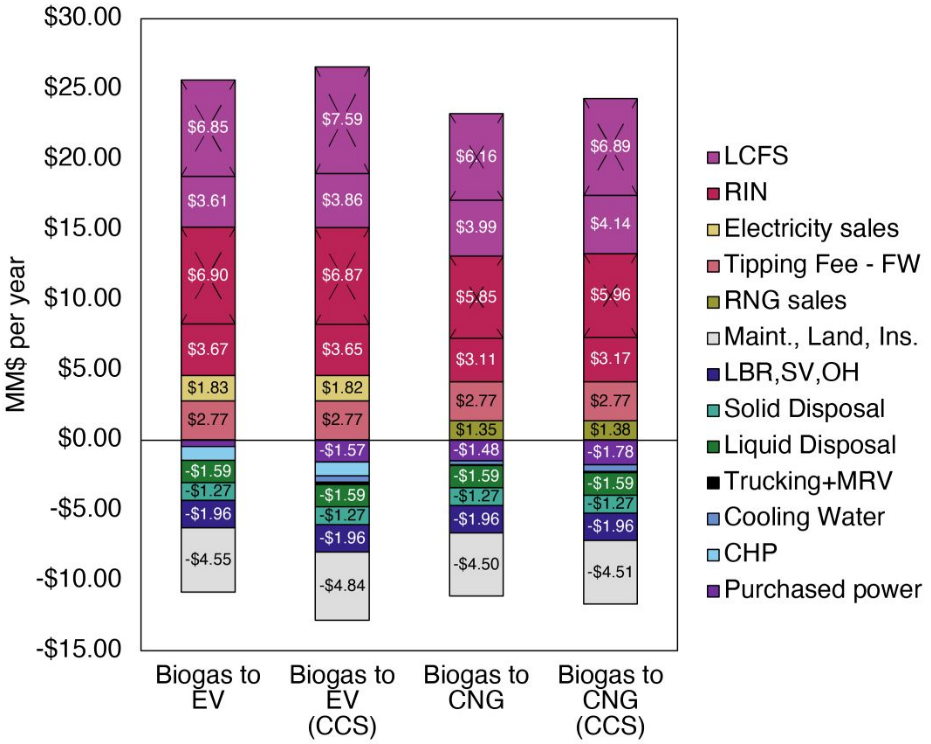

Figure S19.1: Revenues and OPEX during year 4 (after the initial startup period, see SI Section 22 for cash-flow assumptions) for each design in this work. All cash flows less than $1MM were not labeled for clarity. LCFS and RFS (RINs) contributions from FW are marked with a dashed "X".

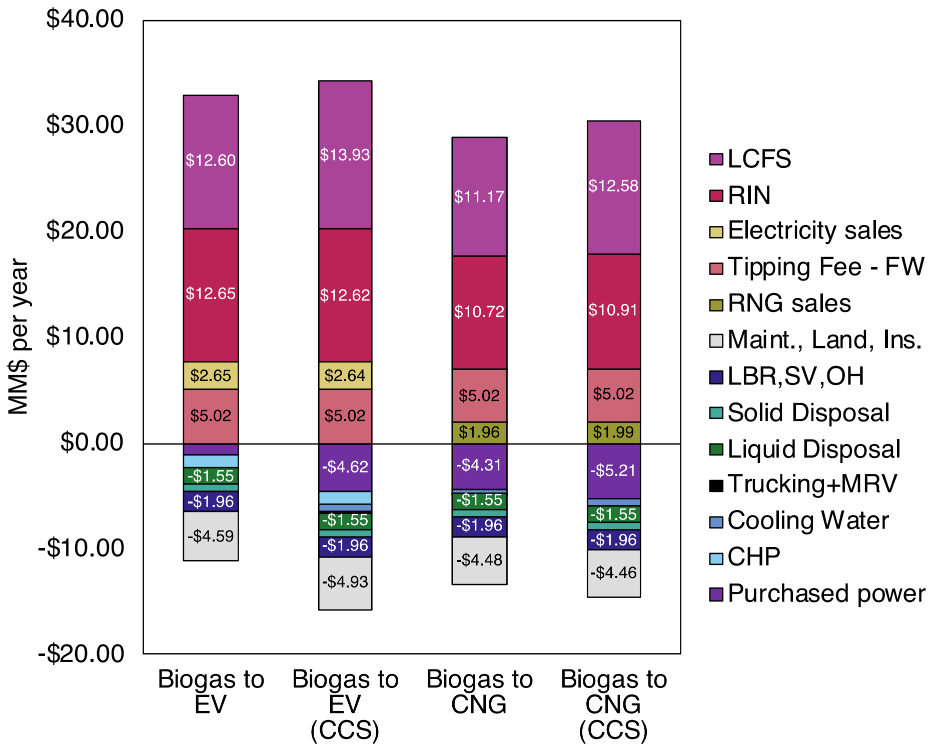

Figure S19.2: Revenues and OPEX for 100%FW designs considered in this work under base policy landscape

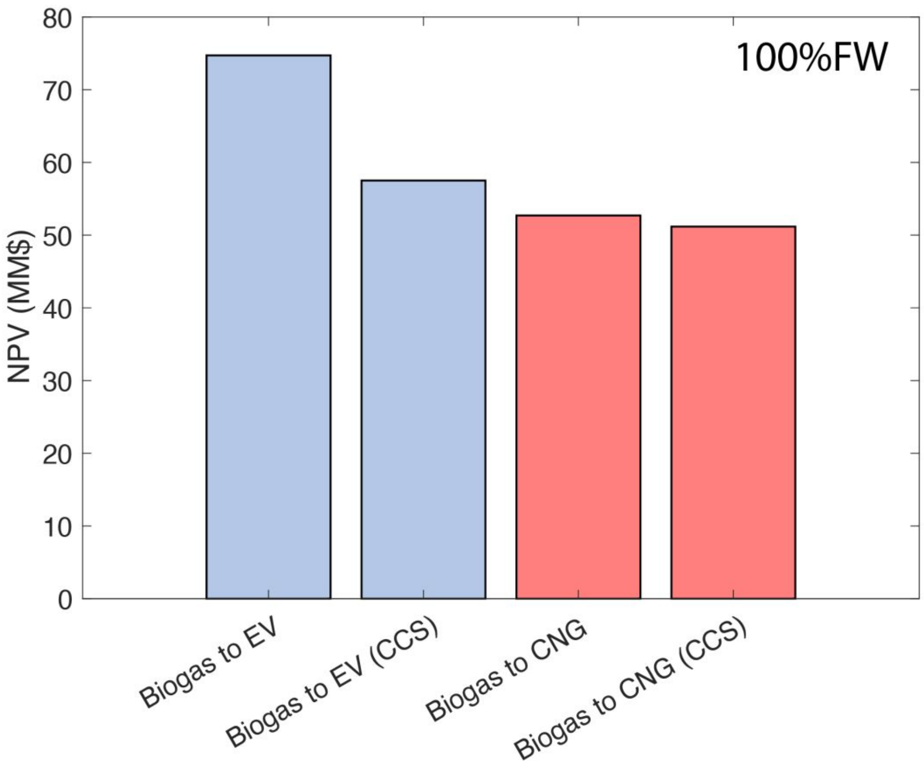

Figure S19.3: Net present values (MM$) of wet AD designs (100%FW) under base policy landscape.

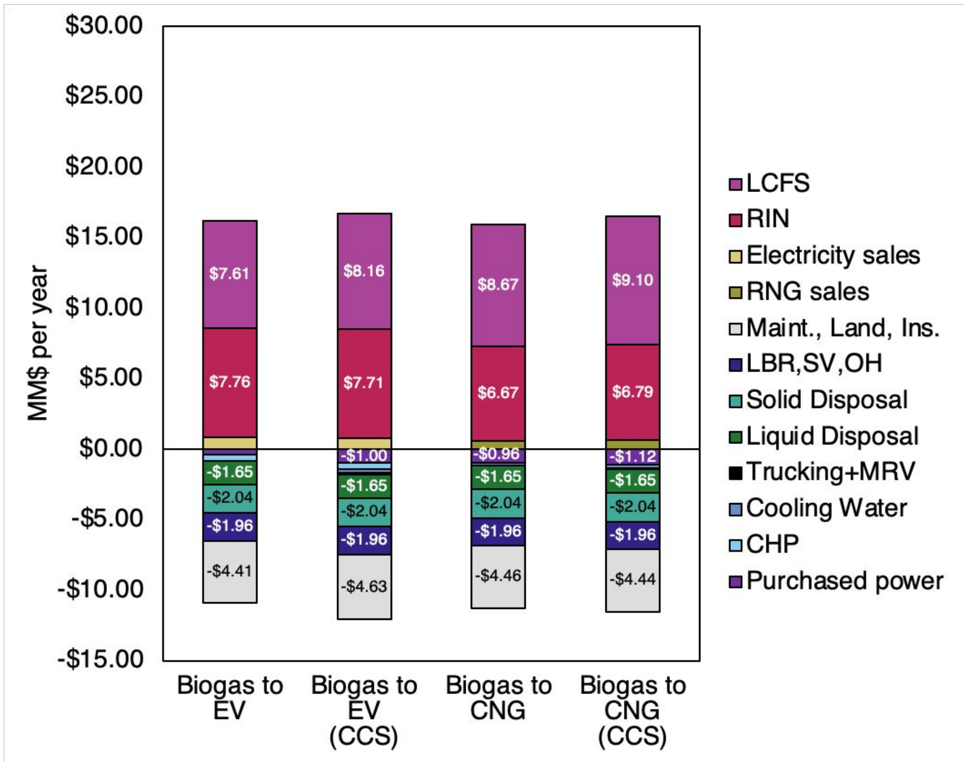

Figure S19.4: Revenues and OPEX for 100%DM designs considered in this work under base policy landscape

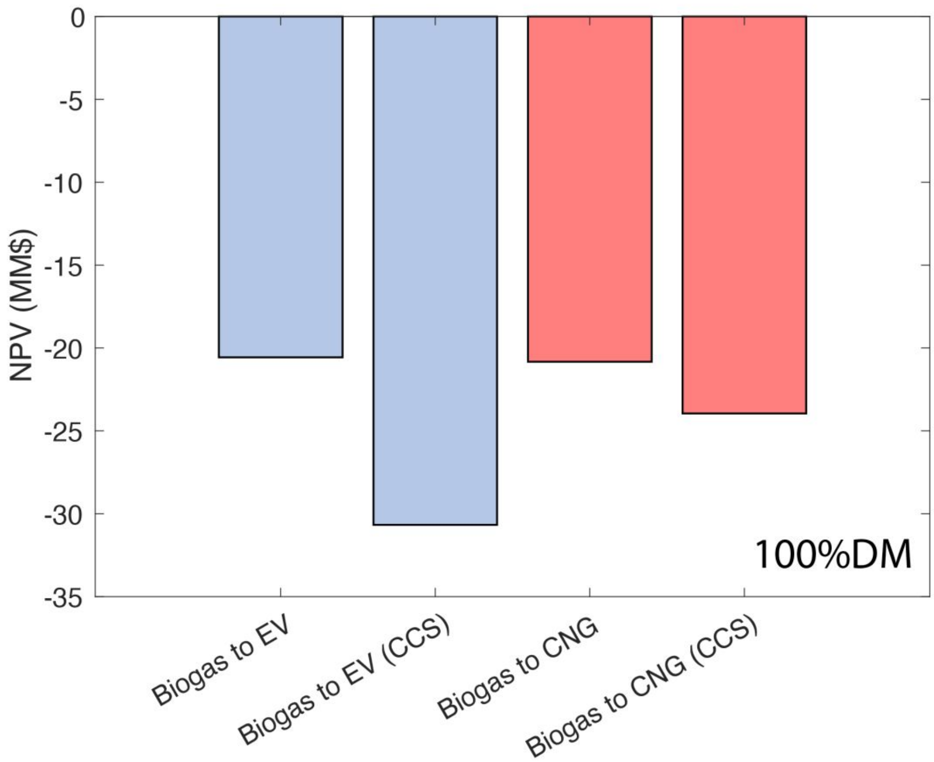

Figure S19.5: Net present values (MM$) of wet AD designs (100%DM) under base policy landscape.

20. Diversion of power to satisfy plant demands parasitically - Biogas to EV (Parasitic)

In this alternate design (**Biogas to EV (Parasitic)**), a portion of the power generated from the CHP unit is diverted to meet on-site demands (such as compression, stirring, and blending power requirements). As a result, this design no longer needs to purchase power from the grid. We have adjusted the CI score to account for product diversion and removed the penalty associated with purchased grid power. 6.3% of power is needed to satisfy plant demands which is in agreement with existing wet AD facilities [77].

The total revenues of **Biogas to EV (Parasitic)** are $1MM/year smaller than for **Biogas to EV.** However, costs are also lower since this design no longer purchases power. However, the NPV is still higher for **Biogas to EV** (Figure S20.2).

This trend will be dependent on the local electricity rate. As electricity prices increase, it may favor a utility configuration in which power is generated on-site instead of purchased. Therefore, we varied the electricity price to equalize the NPVs for both utility configurations. When purchased power is $0.36/kWh, both projects' NPVs are equivalent. This is 225% greater than the assumed electricity rate and is much higher than the ranges seen over multiple sectors in CA [89]. Thus, diverting power parasitically is not economically attractive relative to purchased grid power when transportation fuel credits are being received.

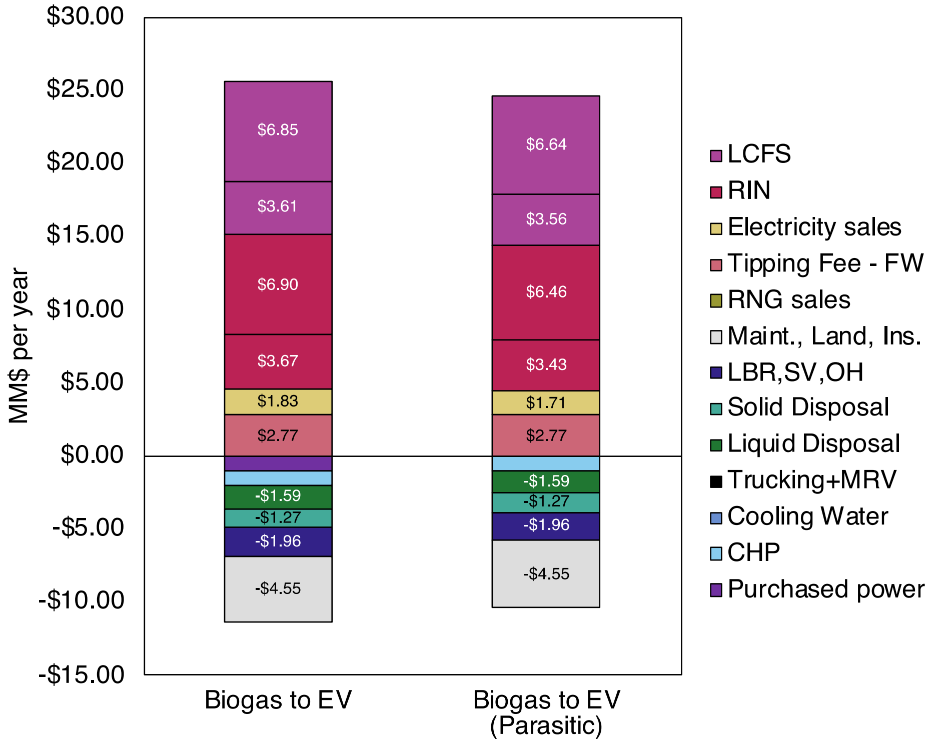

Figure S20.1: Revenues and OPEX for Biogas to EV design (50%FW) when power is diverted parasitically to satisfy plant demands (instead of purchasing grid power). Biogas to EV design with purchased power is shown for reference. The designs shown do not include CCS. For LCFS and RFS revenues, the top bar is the contribution from biogas generated by FW, while the bottom bar is the contribution from DM.

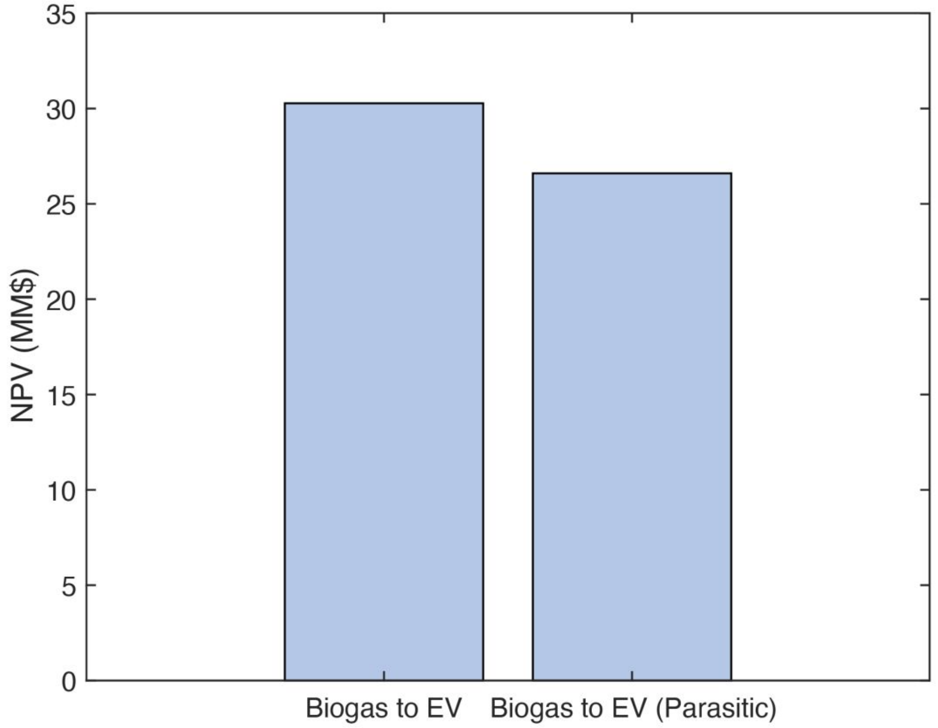

Figure S20.2: NPV comparison between Biogas to EV facilities (without CCS) when power is purchased vs. when a portion of power is diverted to meet facility demands parasitically (parasitic) when digesting 50%FW. Purchased power costs are $0.16/kWh.

21. Discussion of calculated revenues from RFS and LCFS

The RFS uses different equivalence values to calculate RIN generation. The equivalence value for electricity (6.5 kWh of electric power/RIN) was derived using an assumed engine efficiency (28.8%). Thus, more efficient engines will always generate more revenue per MJ of fuel through RINs than from selling the fuel directly. Our estimated engine efficiency is 40% based on existing CHP engines at this scale [45], so eRIN generation is always greater than RIN generation for the same quantity of biogas. This explains why RFS credit revenues are higher for **Biogas to EV**.

For LCFS credits generated from electricity, the California Air Resources Board (CARB) requires multiplying the electricity CI Score by an adjustment factor, derived by taking the actual engine efficiency divided by 50% (the so-called "benchmark efficiency") [72], [73], [90]. However, to convert from gCO_{2e} per MJ of gaseous fuel to per MJ of electricity we divide the CI score by the actual engine efficiency, so the actual engine efficiency cancels out. Then, the adjusted CI score is divided by an Energy Equivalence Ratio (EER) to consider the relative efficiency of an internal combustion engine to an electricity-driven motor. The EER value (3.4) applies to light-duty battery electric vehicles replacing gasoline-powered cars. The culmination of terms results in roughly equivalent LCFS revenues for **Biogas to EV** and **Biogas to CNG**, but this equivalence is mainly fortuitous.

Changing the use case to heavy-duty CNG (EER = 1) or electrically powered trucks and buses (EER = 5) does not alter **Biogas to CNG** LCFS revenues. However, it enhances LCFS revenues for electricity from **Biogas to EV** by 25% ($10.5MM/yr → $13.2MM/yr). Thus, **Biogas to EV** facilities that target vehicles with high EER values can generate more significant LCFS revenues than **Biogas to CNG** for the same quantity of biogas input. The actual engine efficiency and CCS unit performance (CH_4 recovery) would also significantly affect the relative economics between **Biogas to EV** and **Biogas to CNG**. However, we need not modify these parameters here because we explicitly model a specific CCS technology and CHP unit.

22. Cash flow analysis (NPV)

We determined Net Present Value using standard cash flow analyses that can be found in many introductory chemical engineering design texts [21], [38]. Table S22.1 displays the economic assumptions used in the cash flow analysis, as well as other economic assumptions that were held constant across all scenarios such as labor costs, insurance, overhead, etc. The combined federal and CA state tax and depreciation schedule is listed, but they were not utilized because expenses always exceeded taxable revenues.

Table S22.1: Economic assumptions used in cash-flow analysis or otherwise held constant across all modeled scenarios

Parameter	Value	Unit	Reference
Tax Rate	30	%	CA+Federal
Depreciation Schedule	7-year MACRS		[31]
Project lifetime	20	years	
Working Capital	1/1 in year 0/1, recover in year 20	MM$	[31]
Construction Schedule	70%/30% year 0/1		[31]
Revenue Schedule	90%/100% year2/year3+		[31]
Maintenance costs	5	% of ISBL[1]	[21]
Labor	90,000	$/shift-yr	[31]
Supervision	25	% of labor	[31]
Overhead	45	% of labor and supervision	[31]
Location Factor	1.07		[31]
Insurance	1	% of (ISBL+OSBL)	[31]
Land lease	3.36	$/ft^2/year	[87]
Chemical Engineering Plant Cost Index 2021	735		[91]
Discount Rate	12.5	%	Assumed hurdle rate

[1]ISBL = Inside Battery Limit (Includes installation costs of all equipment)

23. Effect of Energy Economy Ratio (EER) on NPV

While the trends in figure 3 of the main text hold when CNG and electricity replace gasoline in light-duty vehicles (assumed here), they may change when the vehicle/fuel combination is modified. Across all replaced fuels (diesel, gasoline, or jet fuel), **Biogas to CNG** NPVs only vary by ±$0.31MM (±1.7%). **Biogas to EV** NPVs are, at worst, lowered by $8MM (-26%) when replacing diesel fuel with electric ocean-going vehicles (EER = 2.6). For this vehicle/fuel combination, our uncertainty in CHP CAPEX (Figure 3 error bars) now lies within the **Biogas to CNG** design, so we can no longer determine which design is optimal. When EERs are 2.8 or greater, **Biogas to EV** designs remain more profitable than **Biogas to CNG** designs within our estimated uncertainties (Figure S23). Lastly, we note that profits are maximized for a vehicle/fuel combination of electric BEV/PHEV trucks/buses (EER = 5, NPV = $47MM) that replace conventional buses running on diesel fuel.

Energy economy ratios (EERs) developed by the California Air Resources Board compares the energy used by an alternative fueled vehicle to a comparable conventional diesel or gasoline vehicle. We report the effect of the EER on the NPV for **Biogas to EV** and **Biogas to CNG** designs in Figure S23. In Table S23, we display the EERs of well-known vehicle/fuel combinations as reported by CARB.

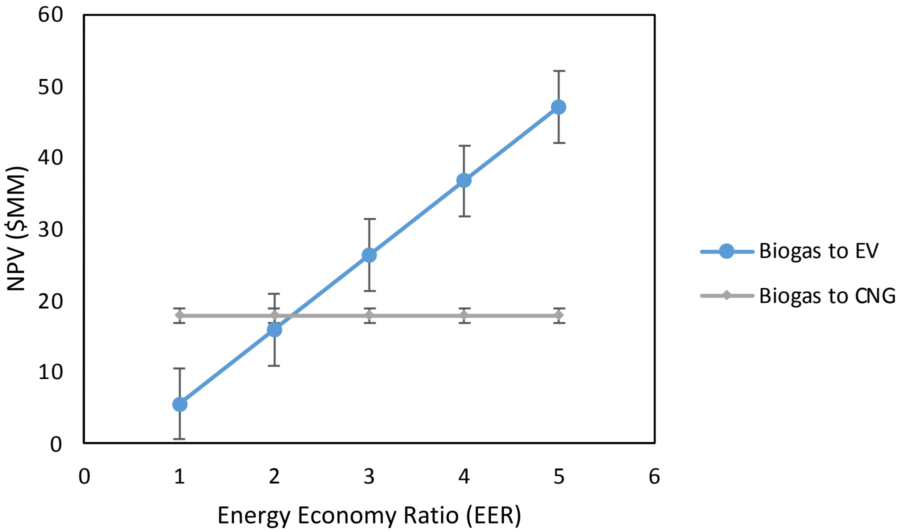

Figure S23: Effect of EER on NPV for Biogas to EV and Biogas to CNG (50%FW) when replacing diesel fuel. Error bars are ±$5MM for Biogas to EV and ±$1MM for Biogas to CNG based on estimated uncertainties in CHP and CCS CAPEX.

Table S23: EERs of common vehicle/fuel types (not exhaustive) taken from [72]

Fuel replaced	Vehicle/Fuel Combination	EER
	CNG	1
**Gasoline**	Electricity BEV/PHEV[1]	3.4
	On-Road Electric Motorcycle	4.4
	Electricity BEV/PHEV Truck or Bus	5.0
	Electric Heavy Rail	4.6
**Diesel**	Electric Light Rail	3.3
	Electric Trolley/Cable Car/Street Car	3.1
	Electric Forklift	3.8

	Electric ocean-going Vessel (eOGV)	2.6

[1]BEV = Battery Electric Vehicle, PHEV = Plug-in Hybrid Electric Vehicle

24. Breakdown of incremental costs and revenues from CCS as a function of scale

We investigated the impact of facility scale on the incremental costs/revenues for CCS on **Biogas to CNG** designs (Figure S24). We found that scale only impacts the CAPEX component, while other components remain essentially unchanged. Although only shown for **Biogas to CNG**, this is also true for **Biogas to EV.**

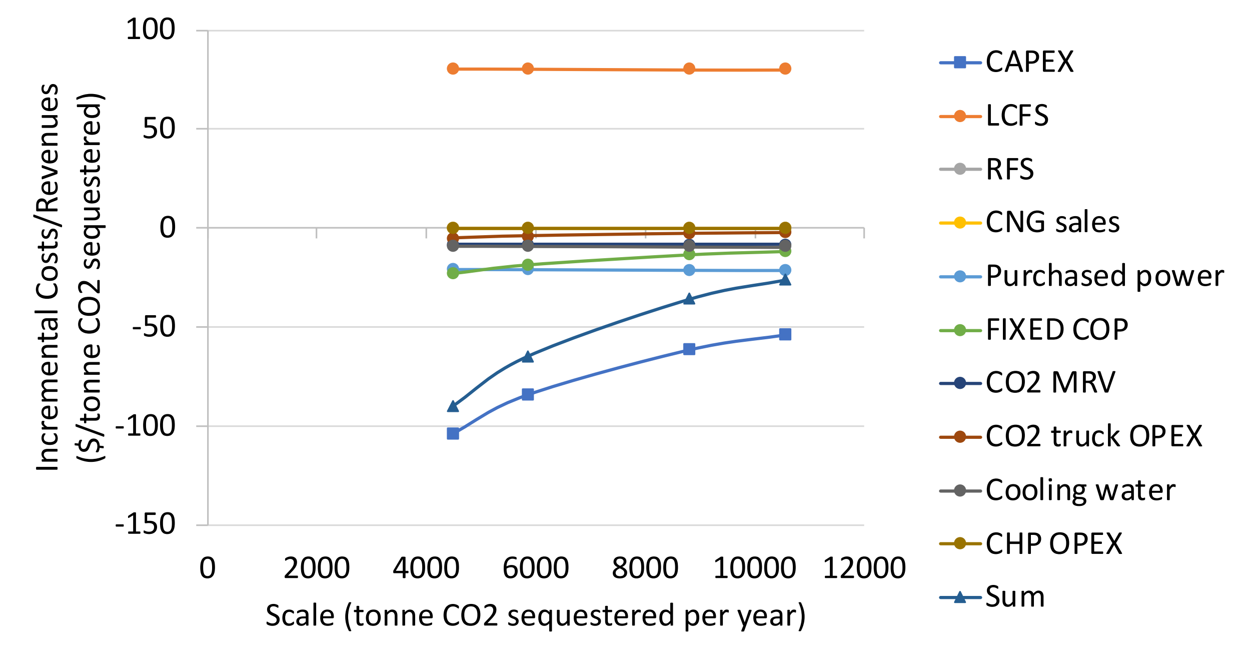

Figure S24: Effect of scale on incremental costs/revenues for adding CCS to Biogas to CNG designs. Scales were modified by changing waste compositions and input waste flowrates. These plots were constructed for 40kton/year dry waste input with waste compositions ranging from 0%FW to 100%FW. The smallest scale was computed for 0%FW at 30kton/year dry waste input.

25. Sensitivity analysis of parameters that govern CCS feasibility with wet AD designs

Because biogas facilities produce CO_2 at relatively small flowrates [92], we calculated the minimum credit as a function of facility scale (Figure S25.1). This plot was constructed by varying waste compositions from 0%FW to 100%FW (which affords a 5% change in CO_2 mole percent) and waste input (see caption). Since all lines fall on a curve with minimal scatter, the minimum required credit is not strongly dependent on biogas composition, only CO_2 flowrate. The steep rise in specific costs at scales less than 6000 tonnes/year originates from steeply increasing CAPEX of the CO_2 liquefaction unit and tube trucks (Figure S24). In our analysis, a few **Biogas to CNG** facilities surpass the minimum threshold and are profitable with the 45Q (green zone), while none are for **Biogas to EV**. Many **Biogas to CNG** facilities could be profitable but are ineligible (yellow zone). Based on these results, several existing AD facilities (CalBio, Aemetis) could profitably perform CCS if the threshold were lowered – we discuss these opportunities in SI Section 26.

We note that the minimum CO_2 sequestration credit is a weak function of the initial CI score of the fuel, RFS credit values (both D3 and D5), waste tipping and disposal fees, waste composition (for a fixed CO_2 flowrate), CO_2 mole fraction in the biogas, CNG and electricity sale prices, CHP engine electrical efficiency, and vehicle/fuel combination (EER value).

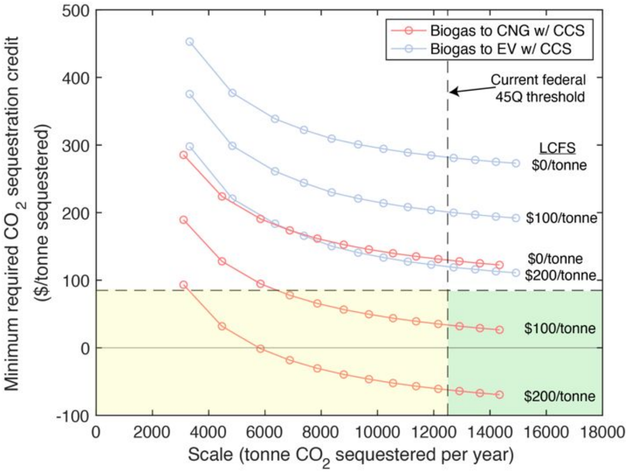

Figure S25.1 Effects of CO_2 production rates on the minimum required CO_2 sequestration (right). Different LCFS credit values are considered parametrically. The green zone indicates that CCS is profitable and meets the federal threshold requirements to earn the 45Q. Yellow means that CCS is profitable, but the threshold is not met. The horizontal dashed line marks the 45Q credit value, $85/tonne, and white indicates that CCS is not profitable. These plots were constructed for 40kton/year dry waste input with waste compositions ranging from 0%FW to 100%FW. The two smallest scales were computed for 0%FW at 20kton/year and 30kton/year dry waste input.

We could expect an uncertainty of ±30% for the CAPEX correlations employed in this work [21]. According to the US Energy Information Administration, average electric utility rates range from ±25% across end-use sectors (residential, industrial, commercial, and transportation) [89]. We use this broad range to test the sensitivity of the electricity rate.

The minimum CO_2 sequestration credit uncertainty for the **Biogas to EV** design is double that of **Biogas to CNG** (Figure S25.2). Even given this uncertainty, at our base scale and LCFS credit value ($110/tonne), we would still conclude that CCS is not feasible for **Biogas to EV** facilities. This can be readily seen by comparing the best-case CO_2 sequestration credit value for **Biogas to EV** (Left bar of "Total uncertainty", ~$135/tonne) to the worst-case for **Biogas to CNG** (Right bar, ~$70/tonne).

For **Biogas to EV**, the significant uncertainties arise mostly from electricity prices and CO_2 liquefaction CAPEX. For **Biogas to CNG**, CO_2 liquefaction CAPEX dominates. There is no uncertainty in membranes unit CAPEX for **Biogas to CNG** because membranes are installed regardless of the choice to perform CCS.

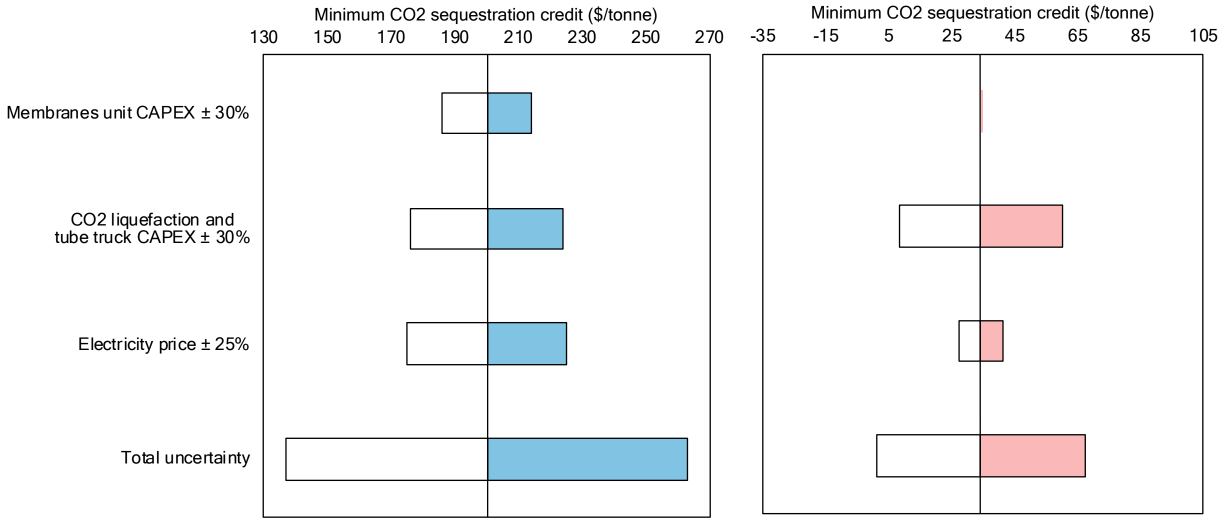

Figure S25.2: Uncertainty in significant parameters that govern minimum CO_2 sequestration credit requirements for Biogas to EV (blue, left) and Biogas to CNG (red, right) designs, for 50%FW scenarios.

26. Current facilities in CA that can perform profitable CO_2 sequestration

As shown in Figure S25.1, wet AD facilities that generate greater than 6,000 tonnes/year of CO_2 can be profitable with current 45Q incentives ($85/tonne) when the LCFS trades above $100/tonne. 6,000 tonnes of CO_2 per year corresponds to about 900 Nm^3/hr of raw biogas. For strictly DM operations, this amount of biogas could be generated by a farm with 9000 cows (assuming 100 lbs of wet manure daily per cow). Many dairy digesters in CA are smaller than this, so dairies need to cluster [12], as others have found when considering the production of CNG alone [21]. The Kern Cluster in CA by California Bioenergy (CalBio), where biogas from eight digesters is exported to a centralized upgrading facility, produces 900,000 MMBTU/yr (~5000 Nm^3/hr biogas) of RNG, is eligible to receive 45Q tax credits and could profitably implement a CO_2 sequestration package provided a Class VI well is nearby [40]. An adjacent AD dairy cluster in Buttonwillow (~1500 Nm^3/yr, also CalBio) is not eligible but could combine with the Kern Cluster at a centralized CO_2 liquefaction facility, further reducing specific costs.

Moreover, recent applications for Class VI sequestration wells in Kern County are underway by the California Resources Corporation and, if approved, should furnish a reliable, vicinal CO_2 sink [90]. Near Sacramento, Aemetis Inc. expects to produce 450,000 MMBTU/yr of RNG (~2500 Nm^3/hr biogas) from a cluster of 17 dairies for use in RNG-fueled trucks and power generation at their ethanol production facilities [41]. At this scale, Aemetis could profitably perform CO_2 liquefaction, transportation, and injection with the 45Q at $85/tonne. In 2024, they plan to acquire a Class VI permit for a well to sequester 400 ktonne/yr from their operations, consistent with our conclusions [91].

27. Opportunities for CCS with wet AD designs outside of CA

Our analysis has relied heavily on the LCFS in CA, but similar programs are available in Oregon (OCFP), Washington (WCFS), and under active consideration in several midwestern US states [93], [94]. We note again that without the LCFS, all NPVs are negative, indicating that these designs are not profitable with only federal incentives (the RFS and 45Q). In the last few months, the CA LCFS credit value has dropped to ~$60/tonne (negative NPVs for all designs), but the OCFP credit value has remained above $110/tonne in the last three years [95]. This presents an opportunity for facility managers to selectively mitigate risk by selling CNG or electricity into state-level marketplaces as credit values change, under certain restrictions. This optimizes NPVs dynamically and enhances CCS since all programs mentioned are based on CI scores.

Outside the United States, programs like the CA LCFS exist in Canada and Brazil [96], [97]. We expect more states in the USA to adopt similar programs to the CA LCFS over time.

28. Plotting functions

We created stacked bar plots in MATLAB using the plotBarStackGroups function written by Evan [98].

References

[1] "2020 Fresno County Crop Report," Department of Agriculture and Weights and Measures, The County of Fresno, 2020. Accessed: Sep. 27, 2022. [Online]. Available: https://www.co.fresno.ca.us/departments/agricultural-commissioner/fresno-county-crop-report-dmi

[2] H. M. Breunig, L. Jin, A. Robinson, and C. D. Scown, "Bioenergy Potential from Food Waste in California," *Environ. Sci. Technol.*, vol. 51, no. 3, pp. 1120–1128, Feb. 2017, doi: 10.1021/acs.est.6b04591.

[3] C. Banks, S. Heaven, Y. Zhang, and U. Baier, *Food waste digestion: anaerobic digestion of food waste for a circular economy*. Great Britain? IEA Bioenergy, 2018.

[4] Y. Li, R. Zhang, G. Liu, C. Chen, Y. He, and X. Liu, "Comparison of methane production potential, biodegradability, and kinetics of different organic substrates," *Bioresource Technology*, vol. 149, pp. 565–569, Dec. 2013, doi: 10.1016/j.biortech.2013.09.063.

[5] J. D. Browne and J. D. Murphy, "Assessment of the resource associated with biomethane from food waste," *Applied Energy*, vol. 104, pp. 170–177, Apr. 2013, doi: 10.1016/j.apenergy.2012.11.017.

[6] R. Zhang *et al.*, "Characterization of food waste as feedstock for anaerobic digestion," *Bioresource Technology*, vol. 98, no. 4, pp. 929–935, Mar. 2007, doi: 10.1016/j.biortech.2006.02.039.

[7] C. Liu, W. Wang, N. Anwar, Z. Ma, G. Liu, and R. Zhang, "Effect of Organic Loading Rate on Anaerobic Digestion of Food Waste under Mesophilic and Thermophilic Conditions," *Energy Fuels*, vol. 31, no. 3, pp. 2976–2984, Mar. 2017, doi: 10.1021/acs.energyfuels.7b00018.

[8] H. B. Møller, S. G. Sommer, and B. K. Ahring, "Methane productivity of manure, straw and solid fractions of manure," *Biomass and Bioenergy*, vol. 26, no. 5, pp. 485–495, May 2004, doi: 10.1016/j.biombioe.2003.08.008.

[9] J. H. Ebner, R. A. Labatut, J. S. Lodge, A. A. Williamson, and T. A. Trabold, "Anaerobic co-digestion of commercial food waste and dairy manure: Characterizing biochemical parameters and synergistic effects," *Waste Management*, vol. 52, pp. 286–294, Jun. 2016, doi: 10.1016/j.wasman.2016.03.046.

[10] R. A. Labatut, L. T. Angenent, and N. R. Scott, "Biochemical methane potential and biodegradability of complex organic substrates," *Bioresource Technology*, vol. 102, no. 3, pp. 2255–2264, Feb. 2011, doi: 10.1016/j.biortech.2010.10.035.

[11] M. Adghim, M. Abdallah, S. Saad, A. Shanableh, and M. Sartaj, "Assessment of the biochemical methane potential of mono- and co-digested dairy farm wastes," *Waste Manag Res*, vol. 38, no. 1, pp. 88–99, Jan. 2020, doi: 10.1177/0734242X19871999.

[12] Franklin Miller, "Quote for Franklin Miller Food Waste Shredder model:TM3054." Accessed: Apr. 12, 2022. [Online]. Available: https://franklinmiller.com/shredders/

[13] Pentair Myers, "Quote for Pentair Myers Submersible Grinder Pump VS50." Accessed: Dec. 21, 2021. [Online]. Available: https://www.pentair.com/en-us/products/business-industry/water-disposal-pumps/grinder-pumps/myers-vsx30-vsx50-centrifugal-grinder-pumps.html

[14] H. M. El-Mashad and R. Zhang, "Biogas production from co-digestion of dairy manure and food waste," *Bioresource Technology*, vol. 101, no. 11, pp. 4021–4028, Jun. 2010, doi: 10.1016/j.biortech.2010.01.027.

[15] U.S. Environmental Protection Agency, “AgSTAR Project Development Handbook.” Apr. 2020. Accessed: Apr. 22, 2022. [Online]. Available: https://www.epa.gov/agstar/agstar-project-development-handbook
[16] Engineer at UC Davis Renewable Energy Anaerobic Digester, “Interview with Engineer at UC Davis Renewable Energy Anaerobic Digester,” Mar. 22, 2022.
[17] M. Goodson, “Technical Memorandum 1: EBMUD Digester Repairs and Improvement Project.” Dec. 10, 2001. Accessed: Oct. 04, 2022. [Online]. Available: https://www.ebmud.com/index.php/download_file/force/4647/1201/?Alden_Lane_Nursery2017.pdf
[18] U.S. Environmental Protection Agency, “Volume to Weight Conversion Factors.” Office of Resource Conservation and Recovery, Apr. 2016. Accessed: Oct. 04, 2022. [Online]. Available: https://www.epa.gov/sites/default/files/2016-04/documents/volume_to_weight_conversion_factors_memorandum_04192016_508fnl.pdf
[19] H. Wang, H. A. Aguirre-Villegas, R. A. Larson, and A. Alkan-Ozkaynak, “Physical Properties of Dairy Manure Pre- and Post-Anaerobic Digestion,” *Applied Sciences*, vol. 9, no. 13, p. 2703, Jul. 2019, doi: 10.3390/app9132703.
[20] PRESS TECHNOLOGY & MFG., INC., “Quote for E-16 Agripress, V60 Vibratory screen, flowmeter, and control panel.” [Online]. Available: www.presstechnology.com
[21] G. P. Towler and R. K. Sinnott, *Chemical engineering design: principles, practice, and economics of plant and process design*, 2nd ed. Boston, MA: Butterworth-Heinemann, 2013 p.1-1303
[22] H. Renon and J. M. Prausnitz, “Local compositions in thermodynamic excess functions for liquid mixtures,” *AIChE J.*, vol. 14, no. 1, pp. 135–144, Jan. 1968, doi: 10.1002/aic.690140124.
[23] B. Mock, L. B. Evans, and C.-C. Chen, “Thermodynamic representation of phase equilibria of mixed-solvent electrolyte systems,” *AIChE J.*, vol. 32, no. 10, pp. 1655–1664, Oct. 1986, doi: 10.1002/aic.690321009.
[24] J. D. Seader, E. J. Henley, and D. K. Roper, *Separation process principles with applications using process simulators*, 4th ed. Hoboken: Wiley, 2016 p.1-728
[25] C. Mao, Y. Feng, X. Wang, and G. Ren, “Review on research achievements of biogas from anaerobic digestion,” *Renewable and Sustainable Energy Reviews*, vol. 45, pp. 540–555, May 2015, doi: 10.1016/j.rser.2015.02.032.
[26] F. O. Agyeman and W. Tao, “Anaerobic co-digestion of food waste and dairy manure: Effects of food waste particle size and organic loading rate,” *Journal of Environmental Management*, vol. 133, pp. 268–274, Jan. 2014, doi: 10.1016/j.jenvman.2013.12.016.
[27] L. Zhang, Y.-W. Lee, and D. Jahng, “Anaerobic co-digestion of food waste and piggery wastewater: Focusing on the role of trace elements,” *Bioresource Technology*, vol. 102, no. 8, pp. 5048–5059, Apr. 2011, doi: 10.1016/j.biortech.2011.01.082.
[28] F. J. Andriamanohiarisoamanana *et al.*, “Anaerobic co-digestion of dairy manure, meat and bone meal, and crude glycerol under mesophilic conditions: Synergistic effect and kinetic studies,” *Energy for Sustainable Development*, vol. 40, pp. 11–18, Oct. 2017, doi: 10.1016/j.esd.2017.05.008.

[29] A. M. Buswell and H. F. Mueller, “Mechanism of Methane Fermentation,” *Ind. Eng. Chem.*, vol. 44, no. 3, pp. 550–552, Mar. 1952, doi: 10.1021/ie50507a033.
[30] M. D. Ong, R. B. Williams, and S. R. Kaffka, “DRAFTComparativeAssessmentofTechnologyOptionsforBiogasClean-up,” California Energy Commission, CEC-500-11-020, TASK8.
[31] R. Hreiz, N. Adouani, Y. Jannot, and M.-N. Pons, “Modeling and simulation of heat transfer phenomena in a semi-buried anaerobic digester,” *Chemical Engineering Research and Design*, vol. 119, pp. 101–116, Mar. 2017, doi: 10.1016/j.cherd.2017.01.007.
[32] “One Foot Method.” American Petroleum Institute, 2022. [Online]. Available: https://www.api.org/products-and-services/standards/important-standards-announcements/standard650
[33] J. Lindmark, E. Thorin, R. Bel Fdhila, and E. Dahlquist, “Effects of mixing on the result of anaerobic digestion: Review,” *Renewable and Sustainable Energy Reviews*, vol. 40, pp. 1030–1047, Dec. 2014, doi: 10.1016/j.rser.2014.07.182.
[34] J. R. Welty, Ed., *Fundamentals of momentum, heat, and mass transfer*, 5th ed. Danver, MA: Wiley, 2008 p.250-350
[35] Y. Zhang, “Anaerobic digestion system Energy balance,” University of Southampton, Aug. 15, 2013. Accessed: Oct. 04, 2022. [Online]. Available: https://www.valorgas.soton.ac.uk/Pub_docs/JyU%20SS%202013/VALORGAS_JyU_2013_Lecture%2013.pdf
[36] National Renewable energy Laboratory, “National Solar Radiation Database.” Accessed: Oct. 04, 2022. [Online]. Available: https://nsrdb.nrel.gov/data-viewer/
[37] “National Weather Service.” Accessed: Oct. 04, 2022. [Online]. Available: https://www.weather.gov/
[38] W. D. Seider, Ed., *Product and process design principles: synthesis, analysis, and evaluation*, 3rd ed. Hoboken, NJ: Wiley, 2009 p.1-728
[39] “Online vendor price for biogas chiller.” Zorg Biogas, 2022. Accessed: Oct. 04, 2022. [Online]. Available: https://zorg-biogas.com/equipment/biogas-dryers
[40] Zorg Biogas, “Online vendor price for biogas blower.” Zorg Biogas. [Online]. Available: https://zorg-biogas.com/equipment/biogas-blowers
[41] I. Benkert, “Quote from General Carbon.” General Carbon, Nov. 04, 2021.
[42] Gas Technology Products, “MINI-CAT Information Brochure.” Accessed: Oct. 04, 2022. [Online]. Available: https://d3pcsg2wjq9izr.cloudfront.net/files/8737/download/16311/mini-cat.pdf
[43] Schlumberger, “Schlumberger Sulfatreat.” 2022. [Online]. Available: https://www.slb.com/well-production/production-chemicals-and-services/sulfatreat-h2s-removal-adsorbents/h2s-removal-from-biogas
[44] K. C. Ng *et al.*, “Experimental investigation of the silica gel–water adsorption isotherm characteristics,” *Applied Thermal Engineering*, vol. 21, no. 16, pp. 1631–1642, Nov. 2001, doi: 10.1016/S1359-4311(01)00039-4.
[45] U.S. Environmental Protection Agency, “Catalogue of CHP Technologies.” Sep. 2017. [Online]. Available: https://www.epa.gov/chp/chp-technologies

[46] F. M. Baena-Moreno, E. le Saché, L. Pastor-Pérez, and T. R. Reina, “Membrane-based technologies for biogas upgrading: a review,” *Environ Chem Lett*, vol. 18, no. 5, pp. 1649–1658, Sep. 2020, doi: 10.1007/s10311-020-01036-3.
[47] Evonik, “Evonik Sepuran Green Brochure.” Accessed: Oct. 10, 2022. [Online]. Available: https://www.membrane-separation.com/en/upgrading-of-biogas-to-biomethane-with-sepuran-green
[48] Y. Chu, A. Lindbråthen, L. Lei, X. He, and M. Hillestad, “Mathematical modeling and process parametric study of CO2 removal from natural gas by hollow fiber membranes,” *Chemical Engineering Research and Design*, vol. 148, pp. 45–55, Aug. 2019, doi: 10.1016/j.cherd.2019.05.054.
[49] E. Esposito, L. Dellamuzia, U. Moretti, A. Fuoco, L. Giorno, and J. C. Jansen, “Simultaneous production of biomethane and food grade CO_2 from biogas: an industrial case study,” *Energy Environ. Sci.*, vol. 12, no. 1, pp. 281–289, 2019, doi: 10.1039/C8EE02897D.
[50] Mazzotti, “Membranes IV Non-Ideal Models,” ETH Zürich. [Online]. Available: https://ethz.ch/content/dam/ethz/special-interest/mavt/process-engineering/separation-processes-laboratory-dam/documents/education/RCS_2020/Membranes/Classes/2020_C3-C4_non_ideal_models.pdf
[51] S. C. Chapra and R. P. Canale, *Numerical methods for engineers*, Seventh edition. New York, NY: McGraw-Hill Education, 2015.
[52] D. T. Coker, R. Prabhakar, and B. D. Freeman, “Tools for Teaching Gas Separation Using Polymers,” Chemical Engineering Education Vol. 37 (1), 2003.
[53] V. Vrbová and K. Ciahotný, “Upgrading Biogas to Biomethane Using Membrane Separation,” *Energy Fuels*, vol. 31, no. 9, pp. 9393–9401, Sep. 2017, doi: 10.1021/acs.energyfuels.7b00120.
[54] P. Linstrom, “NIST Chemistry WebBook, NIST Standard Reference Database 69.” National Institute of Standards and Technology, 1997. doi: 10.18434/T4D303.
[55] *Specifications for Alternative Motor Vehicle Fuels*. Accessed: Oct. 01, 2022. [Online]. Available: https://ww2.arb.ca.gov/resources/documents/alternative-fuels-specifications
[56] S. J. Smith, A. J. Satchwell, T. W. Kirchstetter, and C. D. Scown, “The implications of facility design and enabling policies on the economics of dry anaerobic digestion,” *Waste Management*, vol. 128, pp. 122–131, Jun. 2021, doi: 10.1016/j.wasman.2021.04.048.
[57] W. Urban, K. Girod, H. Lohmann, G. Dachs, and C. Zach, “Technologien und Kosten der Biogasaufbereitung und Einspeisung in das Erdgasnetz,” Fraunhofer Institute, 2009. [Online]. Available: https://publica.fraunhofer.de/entities/publication/fab54542-be60-4747-ac6d-aec4c43ba209/details
[58] N. Parker, R. Williams, R. Dominguez-Faus, and D. Scheitrum, “Renewable natural gas in California: An assessment of the technical and economic potential,” *Energy Policy*, vol. 111, pp. 235–245, Dec. 2017, doi: 10.1016/j.enpol.2017.09.034.
[59] F. Bauer, T. Persson, C. Hulteberg, and D. Tamm, “Biogas upgrading - technology overview, comparison and perspectives for the future,” *Biofuels, Bioprod. Bioref*, vol. 7, no. 5, pp. 499–511, Sep. 2013, doi: 10.1002/bbb.1423.
[60] S. Baker *et al.*, “Getting to Neutral: Options for Negative Carbon Emissions in California,” LLNL-TR--796100, 1597217, 997550, Jan. 2020. doi: 10.2172/1597217.

[61] “Interview with Sales Representative at ANGI ENERGY,” Jul. 10, 2022. [Online]. Available: https://www.angienergy.com/
[62] D. Franks, “Interview with Dean Franks at Husky Energy,” Feb. 2019.
[63] Alternative Fuels Data Center, “Natural Gas Fueling Station Locations.” U.S. Department of Energy. Accessed: Oct. 04, 2022. [Online]. Available: https://afdc.energy.gov/fuels/natural_gas_locations.html#/find/nearest?fuel=CNG
[64] M. Huber, A. Harvey, E. Lemmon, G. Hardin, I. Bell, and M. McLinden, “NIST Reference Fluid Thermodynamic and Transport Properties Database (REFPROP) Version 10 - SRD 23.” National Institute of Standards and Technology, 2018. doi: 10.18434/T4/1502528.
[65] S. Jackson and E. Brodal, “Optimization of the CO2 Liquefaction Process-Performance Study with Varying Ambient Temperature,” *Applied Sciences*, vol. 9, no. 20, p. 4467, Oct. 2019, doi: 10.3390/app9204467.
[66] H. Deng, S. Roussanaly, and G. Skaugen, “Techno-economic analyses of CO2 liquefaction: Impact of product pressure and impurities,” *International Journal of Refrigeration*, vol. 103, pp. 301–315, Jul. 2019, doi: 10.1016/j.ijrefrig.2019.04.011.
[67] F. Chen and T. Morosuk, “Exergetic and Economic Evaluation of CO2 Liquefaction Processes,” *Energies*, vol. 14, no. 21, p. 7174, Nov. 2021, doi: 10.3390/en14217174.
[68] R. McKaskle, K. Fisher, P. Selz, and Y. Lu, “Evaluation of Carbon Dioxide Capture Options from Ethanol Plants,” Illinois State Geological Survey, 2018. Accessed: Oct. 04, 2022. [Online]. Available: http://library.isgs.illinois.edu/Pubs/pdfs/circulars/c595.pdf
[69] “Fuel Pathway Table.” California Air Resources Board, Sep. 29, 2022. Accessed: Sep. 20, 2022. [Online]. Available: https://ww2.arb.ca.gov/resources/documents/lcfs-pathway-certified-carbon-intensities
[70] “Zero Waste Energy LOW CARBON FUEL STANDARD Design-Based Pathway Application.” California Air Resources Board, Jan. 04, 2021. Accessed: Oct. 04, 2022. [Online]. Available: https://ww2.arb.ca.gov/sites/default/files/classic/fuels/lcfs/fuelpathways/comments/tier2/d0014_summary.pdf
[71] “Tier 1 Simplified CI Calculator for Biomethane from Anaerobic Digestion of Organic Waste.” California Air Resources Board, Jan. 04, 2019. Accessed: Mar. 22, 2022. [Online]. Available: https://ww2.arb.ca.gov/resources/documents/lcfs-life-cycle-analysis-models-and-documentation
[72] California Air Resources Board, *Low Carbon Fuel Standard Regulation*, vol. 17. [Online]. Available: https://ww2.arb.ca.gov/sites/default/files/2020-07/2020_lcfs_fro_oal-approved_unofficial_06302020.pdf
[73] California Air Resources Board, “Determining Carbon Intensity of Dairy and Swine Manure Biogas to Electricity Pathways.” Oct. 2019. [Online]. Available: https://ww2.arb.ca.gov/sites/default/files/classic/fuels/lcfs/guidance/lcfsguidance_19-06.pdf
[74] California Public Utilities Commission, “Pacific Gas & Electric Company (PG&E) BioMAT Feed-in Tariff.” Accessed: May 22, 2022. [Online]. Available: https://pgebiomat.accionpower.com/_pgebiomat/home.asp
[75] U.S. Energy Information Administration, “Henry Hub Natural Gas Spot Price.”

[76] “Fresno County Disposal Site.” https://www.co.fresno.ca.us/departments/public-works-planning/divisions-of-public-works-and-planning/resources-and-parks-division/landfill-operations (accessed May 22, 2022).
[77] E. Angelonidi and S. R. Smith, “A comparison of wet and dry anaerobic digestion processes for the treatment of municipal solid waste and food waste: Comparison of wet and dry anaerobic digestion processes,” *Water Environ J*, vol. 29, no. 4, pp. 549–557, Dec. 2015, doi: 10.1111/wej.12130.
[78] “City of Kerman Utility Billing Rates.” Accessed: May 22, 2022. [Online]. Available: https://cityofkerman.net/utility-billingrates/
[79] “Commercial electricity rate in Fresno.” https://www.electricitylocal.com/states/california/fresno/ (accessed May 22, 2022).
[80] “Monthly LCFS credit transfer activity reports, 2022.,” *California 731 Air Resources Board.* https://www.arb.ca.gov/fuels/lcfs/credit/lrtmonthlycreditreports.htm (accessed May 18, 2022).
[81] California Air Resources Board, “LCFS Basics.” Accessed: Oct. 04, 2022. [Online]. Available: https://ww2.arb.ca.gov/sites/default/files/2020-09/basics-notes.pdf
[82] “RIN trades and price information, 2022.,” *U.S. Environmental Protection Agency*.
[83] Cornell Law School, *26 U.S. Code § 45Q - Credit for carbon oxide sequestration*. Accessed: May 18, 2022. [Online]. Available: https://www.law.cornell.edu/uscode/text/26/45Q
[84] Committee on Developing a Research Agenda for Carbon Dioxide Removal and Reliable Sequestration *et al.*, *Negative Emissions Technologies and Reliable Sequestration: A Research Agenda*. Washington, D.C.: National Academies Press, 2019, p. 25259. doi: 10.17226/25259.
[85] J. A. Yarmuth, *Inflation Reduction Act of 2022*. 2022. [Online]. Available: https://www.congress.gov/bill/117th-congress/house-bill/5376
[86] H. M. El-Mashad and R. Zhang, “Biogas production from co-digestion of dairy manure and food waste,” *Bioresource Technology*, vol. 101, no. 11, pp. 4021–4028, Jun. 2010, doi: 10.1016/j.biortech.2010.01.027.
[87] “https://www.loopnet.com/.” https://www.loopnet.com/ (accessed May 22, 2022).
[88] “Global BTX and Hydrocarbon Separating Adsorbent Market,” BIS Research, 2020. [Online]. Available: https://bisresearch.com/industry-report/btx-hydrocarbon-separating-adsorbent-market.html
[89] U.S. Energy Information Administration, “Average Price of Electricity to Ultimate Customers by End-Use Sector,” U.S. Energy Information Administration, Jan. 2023. [Online]. Available: https://www.eia.gov/electricity/annual/html/epa_02_10.html
[90] Environmental Protection Agency, “Renewable Fuel Standard (RFS) Program: Standards for 2023–2025 and Other Changes,” EPA, Nov. 2022. Accessed: Dec. 05, 2022. [Online]. Available: https://www.epa.gov/system/files/documents/2022-12/rfs-set-rule-nprm-2022-11-30.pdf
[91] Chemical Engineering Magazine, “Chemical Engineering Plant Cost Index.” 2021. [Online]. Available: https://www.chemengonline.com/pci-home

[92] P. C. Psarras, S. Comello, P. Bains, P. Charoensawadpong, S. Reichelstein, and J. Wilcox, "Carbon Capture and Utilization in the Industrial Sector," *Environ. Sci. Technol.*, vol. 51, no. 19, pp. 11440–11449, Oct. 2017, doi: 10.1021/acs.est.7b01723.
[93] *WASHINGTON CLEAN FUELS PROGRAM*, vol. 70A.535. [Online]. Available: https://app.leg.wa.gov/RCW/default.aspx?cite=70A.535
[94] *OREGON CLEAN FUELS PROGRAM*, vol. 340–253. [Online]. Available: https://secure.sos.state.or.us/oard/displayDivisionRules.action?selectedDivision=1560
[95] "Monthly Credit Transaction Report," Jan. 22, 2023. https://www.oregon.gov/deq/ghgp/cfp/Pages/Monthly-Data.aspx
[96] *Renewable & Low Carbon Fuels Requirement Regulation*, vol. 394/2008. [Online]. Available: https://alpha.gov.bc.ca/gov/content/industry/electricity-alternative-energy/transportation-energies/renewable-low-carbon-fuels
[97] *RenovaBio Program*. [Online]. Available: https://www.gov.br/mme/pt-br/assuntos/secretarias/petroleo-gas-natural-e-biocombustiveis/renovabio-1/renovabio-ingles
[98] Evan, "Plot of Group Stacked Bars." 2022. [Online]. Available: https://www.mathworks.com/matlabcentral/fileexchange/32884-plot-groups-of-stacked-bars
